# Atherosclerosis: From Pathogenesis to Therapeutic Interventions

**DOI:** 10.1002/mco2.71095

**Published:** 2026-10-29

**Authors:** Enge Zhou, Chenxi Zhan, Yuting Li, Yi Zeng, Renfei Luo, Xin Ouyang, Shixing Liu, Renqiang Yang, Nianlong Yan, Long Jiang

**Affiliations:** ^1^ Department of Cardiovascular Medicine, The Second Affiliated Hospital, Jiangxi Medical College Nanchang University Nanchang Jiangxi China; ^2^ Department of Biochemistry and Molecular Biology, School of Basic Medical Sciences, Jiangxi Medical College Nanchang University Nanchang Jiangxi China; ^3^ Affiliated Rehabilitation Hospital of Nanchang University Nanchang Jiangxi China

**Keywords:** adaptive immunity, atherosclerosis, endothelial dysfunction, immunotherapy, innate immunity, precision management

## Abstract

Atherosclerosis is no longer viewed solely as a lipid‐storage disorder but rather as a complex multisystem inflammatory disease driven by interactions among the vascular, immune, metabolic, and nervous systems. This review integrates recent concepts, including trained immunity, the neuroimmune cardiovascular interface, vascular cell plasticity, and the microbiome–vascular axis, that reshape the understanding of plaque initiation, progression, and instability. We summarize how established lipid‐lowering, antidiabetic, antihypertensive, and revascularization strategies influence atherosclerotic disease and discuss emerging interventions, including RNA therapeutics, anti‐inflammatory agents, gene therapy, and cell‐based approaches. Key ongoing clinical trials that may clarify the cardiovascular benefits and safety of these strategies are also reviewed. Despite therapeutic advances, residual cardiovascular risk persists because of limitations in animal models, sex‐related differences in plaque phenotype, and the lack of clinically applicable biomarkers. Nanotechnology, human microphysiological systems, spatial multiomics, and artificial intelligence may help bridge these translational gaps. Successful clinical implementation will nevertheless require biomarker‐guided patient selection, rational combination therapy, validated surrogate endpoints, long‐term safety assessment, and attention to health economics. In summary, these advances will shape future translational research and clinical trial design, ultimately advancing personalized management of atherosclerosis.

## Graphical abstract

The diagram outlines the core mechanisms driving atherosclerosis, including endothelial dysfunction, inflammatory responses, phenotypic switching of smooth muscle cells, and the gut–vascular axis. It also presents the corresponding therapeutic approaches now under development, such as gene therapies, immunotherapies, and nanotechnology platforms. Together, the illustration highlights the progress of atherosclerosis pathogenesis and new intervention strategies to promote plaque stability and prevent clinical events.

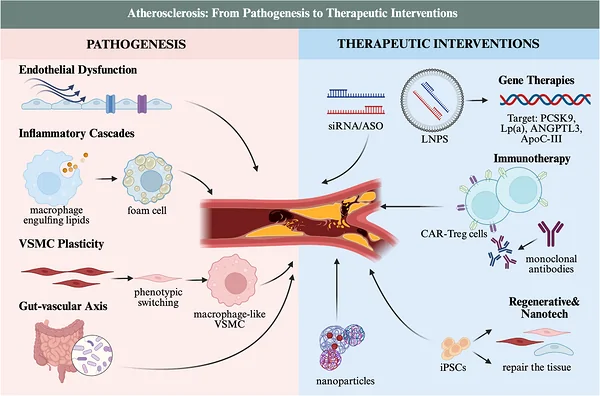

## 1 Introduction

Cardiovascular disease (CVD) remains the leading cause of death worldwide. A recent analysis on the burden of CVD from the Global Burden of Disease (GBD) study showed that the number of patients with CVD in 2023 reached 626 million, with disability‐adjusted life years (DALYs) and deaths reaching 437 million and 19.2 million, respectively [1]. Atherosclerosis (AS) is the main pathophysiological driving factor of clinical CVD, and its formation may be the result of interactions among metabolic, immune and neural physiological systems [2]. Traditional treatment strategies, such as statins and proprotein convertase subtilisin/kexin type 9 (PCSK9) inhibitors, focus mainly on reducing blood lipid levels to control the progression of AS and have greatly advanced the treatment of CVDs. Despite the use of potent lipid‐lowering strategies, including novel drugs to reduce low‐density lipoprotein cholesterol (LDL‐C) to target levels, a certain proportion of patients still experience major adverse cardiovascular events (MACEs), indicating the presence of other residual cardiovascular risks [3]. Although treatments to reduce systemic inflammation have been proven to improve the prognosis of some CVDs, none of the current treatments can directly address the local and multifaceted pathological processes occurring in the arterial wall [4]. This suggests that the progression of AS is not only driven by the complex interactions of endothelial dysfunction (ED), lipid accumulation, and a continuous inflammatory cascade but also affected by nerves, immunity, and the gut microbiota. Against this background, the current treatment methods cannot completely address this residual risk [5]. As the understanding of the mechanism of AS has continued to improve, the current research direction has therefore shifted from traditional lipid‐lowering interventions to more precise multidimensional integration strategies, such as immune metabolic reprogramming, spatial‐level gene expression regulation, neuroimmune interactions, and the microbiome–vascular axis, with the goal of achieving precise intervention and personalized management of AS [6, 7].

The concept of AS has undergone an evolution in the past century, from a disease caused by high‐cholesterol levels to a dynamic, multisystem inflammatory disease. In the early 20th century, Anitschkow reported that feeding rabbits a high‐cholesterol diet could lead to AS, which established the classic cholesterol theory [8]. At the end of the 20th century, Ross integrated these observations and proposed that “AS is an inflammatory disease” [9]. The CANTOS trial (2017) provided the first direct clinical evidence that targeting inflammation reduces cardiovascular events [10]. The past 5 years have brought considerable progress in understanding the mechanisms underlying AS, marked by several conceptual advances. First, the discovery of the neuroimmune–cardiovascular interface (NICI) showed that AS involves not only local vascular inflammation but also that the peripheral nervous system can interact with atherosclerotic intimal plaques through adventitial immune cells [2]. Second, the concept of trained immunity has reshaped the understanding of AS. Innate immune cells can obtain long‐term, highly reactive “memory” through metabolic and epigenetic reprogramming after exposure to atherogenic stimuli such as oxidized low‐density lipoprotein (ox‐LDL). Trained immunity therefore links transient metabolic stimuli to persistent vascular inflammation, which may explain the chronic progression of AS [11]. Third, single‐cell spatial transcriptomics, unlike traditional transcriptomics, resolves the spatial location of cells. In the past 2 years, through the integration of multiplatform analyses of human coronary and carotid plaques, the spatial distribution of immune cells and stromal cells, the cell neighborhood interaction network, and the differentiation trajectory of various cell subtypes in different regions of plaques have been found at the single‐cell level for the first time, providing a complex molecular map for explaining the local immune response and plaque heterogeneity of AS [12, 13]. Fourth, the concept of the oral–gut–vascular axis has improved the understanding of the role of the oral and intestinal microbiome in AS. Studies have shown that the oral microbiome can directly translocate into the vascular wall to affect plaques and that the intestinal microbiota can also regulate the development of AS. On this basis, the oral–gut–vascular axis extends the view of AS beyond the vessel wall and identifies microbial targets for therapy [14, 15]. Fifth, single‐cell technologies have clarified the contribution of adaptive immunity in AS. High‐resolution profiling has revealed clonal expansion, exhaustion, and effector differentiation in lesional T cells, along with the accumulation of age‐associated B cells and adventitial tertiary lymphoid structures, suggesting that adaptive immune circuits are attractive therapeutic targets [16, 17, 18, 19]. Sixth, clonal expansion has prompted a re‐evaluation of vascular smooth muscle cells (VSMCs) involvement in plaque pathogenesis. Lineage‐tracing and single‐cell analyses have shown that a small pool of modulated VSMCs can undergo clonal outgrowth and eventually become a dominant cellular component of the lesion, a finding that challenges the conventional view of uniform phenotypic switching and reveals additional layers of plaque complexity [20, 21]. AS is therefore best viewed as a multidimensional disease involving neuroimmune crosstalk, innate and adaptive immunity, metabolic reprogramming, the microbiome, and VSMC clonal dynamics.

This review focuses on recent mechanistic and conceptual updates in AS, particularly breakthroughs in trained immunity, adaptive immune regulation, VSMC plasticity, and the microbiome–vascular axis. On this basis, we discuss the current treatment strategies, emerging intervention methods and clinical trial progress and then analyze the key challenges and technical solutions involved in translating basic research into clinical research to provide a reference for future research directions in this field.

## 2 Pathogenesis: Canonical Concepts and Recent Advances

AS is a chronic inflammatory disease driven by complex interactions among vascular dysfunction, lipid accumulation, immune activation, and cellular plasticity. The disease begins with ED and subendothelial lipoprotein retention, followed by monocyte recruitment, foam‐cell formation, VSMCs remodeling, and progressive plaque destabilization (Figure 1). Building on this pathological continuum, the following sections discuss the major molecular and cellular mechanisms of AS, with particular emphasis on recent advances in ED, lipid metabolic reprogramming, inflammatory cascades, VSMC plasticity, and the microbiome–vascular axis.

**FIGURE 1:**
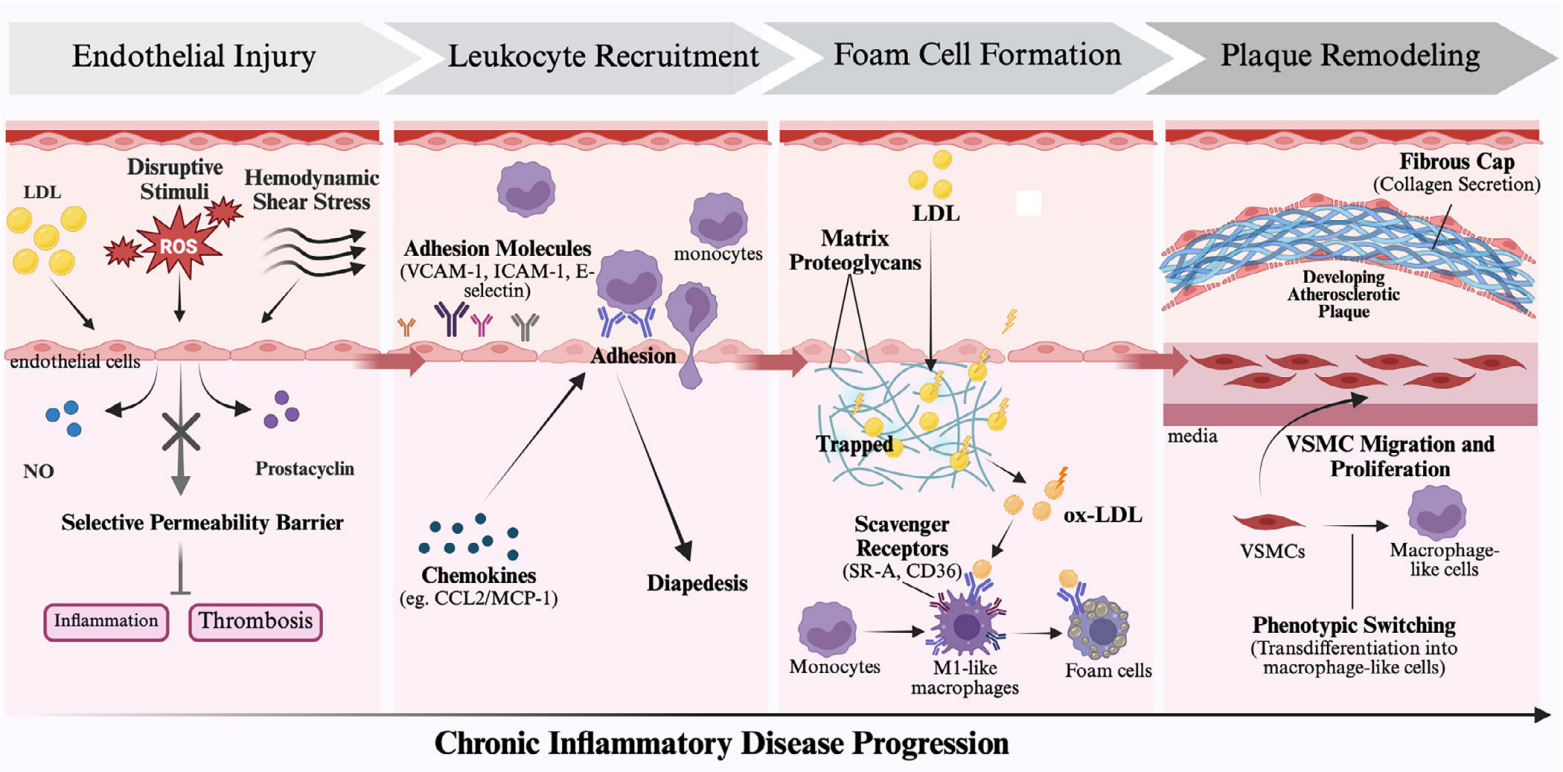
Initiation and progression of the atherogenic continuum. Disruptive stimuli first cause dysfunction in the endothelium, impairing its protective functions. This leads to leukocyte recruitment via adhesion molecules and chemokines. Recruited monocytes differentiate into macrophages, which take up trapped ox‐LDL via scavenger receptors to become foam cells. Finally, plaque remodeling involves VSMC migration, proliferation, and phenotypic switching, contributing to plaque development.

### 2.1 Endothelial Dysfunction

#### 2.1.1 Hemodynamic Determinants and Molecular Pathogenesis

ED is not merely a passive consequence of cardiovascular risk factors but also a central driver of AS, contributing to disease progression from lesion initiation to clinical manifestation [22]. Constitutive nitric oxide (NO) generation is one of several major protective mechanisms used by a healthy endothelium to preserve vascular integrity and control vascular homeostasis at baseline by maintaining vasodilation, preventing platelet aggregation, inhibiting the growth of VSMCs, and preventing the attachment of leukocytes to the vessel wall. This activity is supplemented by the activities of other mediators, such as prostacyclin and tight regulation of the renin–angiotensin system [23]. ED reflects a homeostatic imbalance initiated by injurious stimuli and fueled by a self‐perpetuating cycle of inflammation, oxidative stress, and progressive vascular damage. Hemodynamic forces are among the most important spatial determinants of endothelial injury. Atherosclerotic plaques preferentially develop at arterial sites exposed to disturbed, nonlaminar flow, such as at curvatures, bifurcations, and branch points. In contrast, high, atheroprotective laminar shear stress (LSS) produced by unidirectional laminar flow in straight, unbranched segments promotes a quiescent, anti‐inflammatory endothelial phenotype. Disturbed flow(DF), however, creates low‐magnitude, oscillatory shear stress (OSS) that inhibits endothelial gene expression and function, promoting a proadhesive, proliferative state. Thus, while systemic risk factors create a generalized proatherogenic milieu, the DF acts as a “localizing factor,” priming specific anatomic sites for initial lesion development [24, 25].

Specialized mechanosensors, such as the junctional mechanosensory complex of PECAM‐1, VE‐cadherin, and VEGFR2 [26], along with other critical players, including specific integrins [27], the endothelial glycocalyx [28], various GPCRs [29], and the ion channel PIEZO1 [30], convert distinct shear stress patterns into intracellular biochemical signals on the endothelial cell surface (Figure 2). The role of PIEZO1 is highly context dependent: under protective LSS, it can activate signals that upregulate expression of mitochondrial SIRT4, which helps curb ox‐LDL uptake and oxidative stress [31], whereas under atherogenic OSS, it triggers a distinct Ca^2^^+^/calmodulin/CaMKII‐FAK/Src signaling cascade that leads to the activation and nuclear translocation of Yes‐associated protein (YAP) [32, 33]. YAP, a key effector of the Hippo pathway, acts as a master transcriptional regulator, driving proinflammatory, proliferative, fibrotic, and senescence programs [34, 35]. Its activation is intricately controlled by multiple additional pathways, including the MST1–connexin‐43 axis [36], phase‐separated DDR1 condensates [37], glycocalyx‐mediated CD44 signaling [38], and signaling through the serotonin receptor 5‐HT1B [39]. GPCRs also contribute in a context‐specific manner: for instance, GPR30 (GPER1) activation is considered atheroprotective, partly through the induction of Krüppel‐like factor 2 (KLF2) [40], whereas GPR68 [41] and G protein‐coupled receptor kinase 2 (GRK2) [42] propagate inflammatory signals. Recent additions to the repertoire of known mechanosensors include the redox‐regulated kinase TNIK [43] and the semaphorin receptor Plexin D1 [44].

**FIGURE 2:**
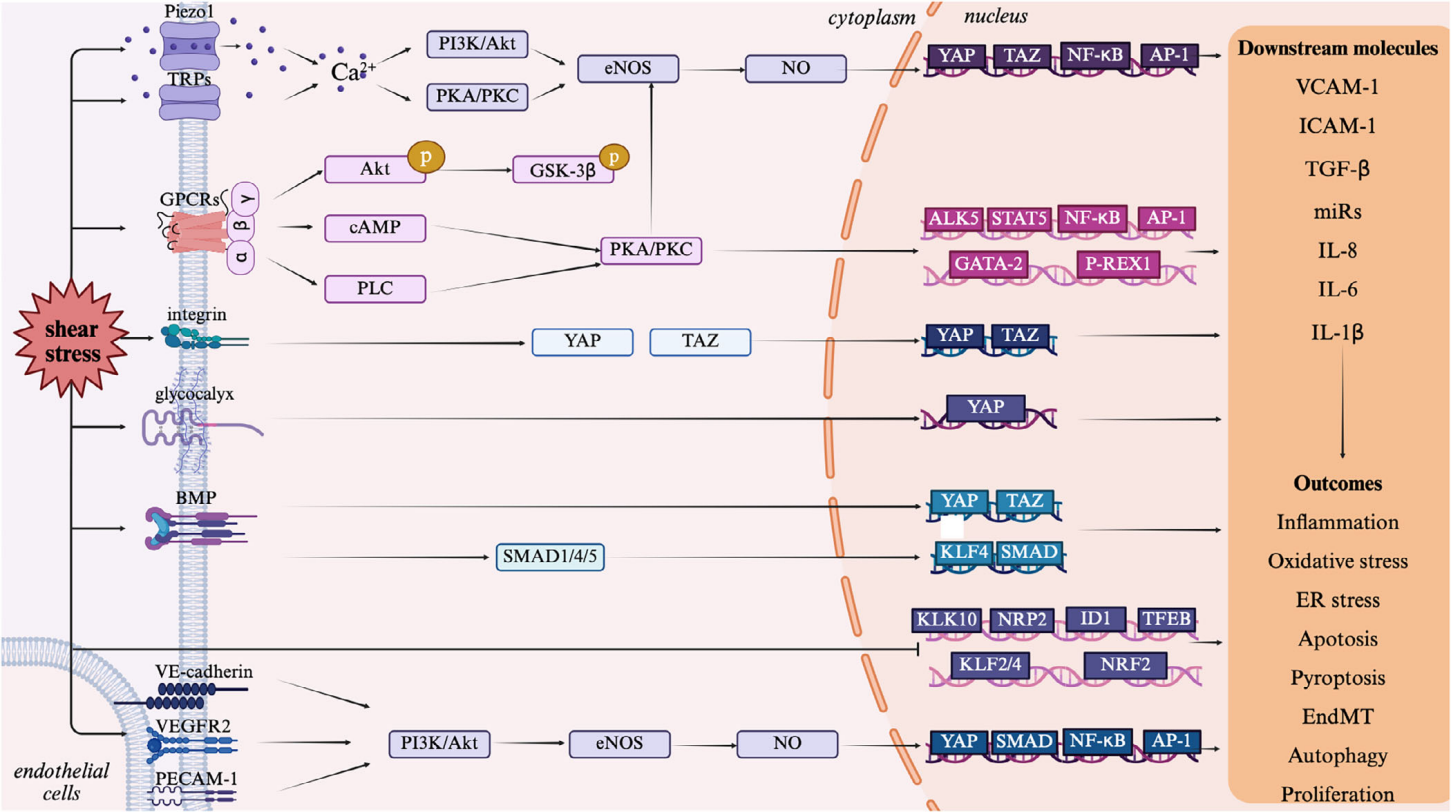
Mechanotransduction pathways and cellular responses to shear stress in endothelial cells. Shear stress is sensed by endothelial membrane mechanosensors, including ion channels, GPCRs, integrins, the glycocalyx, BMP receptors, and the VE‐cadherin/VEGFR2/PECAM‐1 junctional complex. These mechanosensors activate intracellular signaling pathways that regulate nuclear transcription factors and downstream adhesion molecules and inflammatory mediators. The resulting transcriptional responses contribute to inflammation, oxidative stress, ER stress, apoptosis, pyroptosis, EndMT, autophagy, and proliferation, thereby influencing endothelial homeostasis and atherosclerosis progression.

Sustained ED is maintained through two interconnected processes: inflammatory signaling networks that drive acute endothelial activation and epigenetic reprogramming mechanisms that stabilize these pathological states over time. Among the inflammatory pathways, NF‐κB functions as a central hub linking disturbed‐flow signaling to maladaptive endothelial responses. The canonical pathway for NLRP3 inflammasome activation, a key driver of IL‐1β maturation, requires an NF‐κB‐dependent priming step (inducing NLRP3 and pro‐IL‐1β expression) and a second signal (e.g., lysosomal cathepsin B or mitochondrial reactive oxygen species [ROS]) that triggers the assembly of the inflammasome complex, leading to gasdermin D (GSDMD) pore formation, pyroptosis [45], and the release of mature IL‐1β [46]. Intriguingly, a noncanonical NF‐κB pathway (dependent on NIK and RelB) can directly upregulate GSDMD and caspase‐1 expression via interferon regulatory factor 1 (IRF‐1), bypassing the need for the NLRP3 sensor itself [47]. Organelle dysfunction sustains this cycle of inflammation. For example, S‐nitrosylation of the chaperone protein Hsp90 increases oxidative stress by impairing its ability to fold mitochondrial proteins and deal with ROS [48]; monoamine oxidase A (MAO‐A) and mtDNA leakage into the cytosol activate STING–PERK signaling in the endoplasmic reticulum, further integrating stress responses [49, 50]. Furthermore, a subset of CD163^+^ macrophages can induce NF‐κB/Snail‐driven endothelial‐to‐mesenchymal transition (EndMT) in endothelial cells, which promotes the loss of endothelial identity and the acquisition of mesenchymal, profibrotic traits that lead to fibrous cap thinning and plaque vulnerability [51]. Collectively, these inflammatory and stress‐responsive pathways not only drive acute endothelial activation but also establish a permissive environment for more durable phenotypic alterations. In addition to acute inflammatory signaling, DF induces durable epigenetic reprogramming characterized by alterations in chromatin accessibility, histone modifications, DNA methylation, and noncoding RNA networks. Recent studies have demonstrated that AP‐1‐dependent chromatin remodeling [52], EZH2‐mediated H3K27me3 deposition [53], DNMT1‐driven DNA methylation [54], and lncRNA‐guided chromatin regulation [55] establish persistent endothelial phenotypes and provide a mechanistic basis for endothelial memory during atherogenesis. Among these regulatory mechanisms, noncoding RNAs have emerged as important modulators of endothelial phenotypic stability. Endothelial‐enriched microRNAs, particularly miR‐181a2b2 [56] and miR‐126 [57], are vital regulators of endothelial inflammation, apoptosis, and vascular homeostasis, further highlighting the contribution of noncoding RNA networks to endothelial phenotypic stability and atherogenesis.

#### 2.1.2 Pathophysiology in Metabolic and Vascular Disease

AS is a major macrovascular complication of diabetes, and ED serves as a central pathogenic link among hyperglycemia, metabolic dysfunction, and the development of diabetic vascular complications [58, 59]. Youth‐onset type 2 diabetes, in which coronary ED appears early, provides supporting evidence. Plasma‐derived extracellular vesicles from these patients directly induce oxidative stress and endothelial inflammation, supporting their pathogenic role in early vascular dysfunction [60]. Endothelial homeostasis is tightly maintained by a delicate balance of transcriptional and metabolic regulators, which is often disturbed under hyperglycemic conditions. For instance, the transcription factor KLF11 acts as an important gatekeeper, hindering TXNIP‐induced oxidative stress and blocking Notch1/Snail‐mediated EndMT [61]. Hyperglycemia induces persistent alterations in endothelial phenotypes through multilayered epigenetic modifications. In human aortic endothelial cells, high glucose levels upregulate expression of EZH2 to increase repressive H3K27me3 deposition at antioxidant promoters (SOD and JunD), driving oxidative stress and downstream NF‐κB activation [62]. Simultaneously, metabolic lactate accumulation induces protein lactylation; specifically, IDH2 lactylation at K272 alters the Cav1–eNOS interaction to regulate endothelial eNOS activity and angiogenesis under diabetic conditions [63]. These epigenetic changes offer direct therapeutic targets. The bromodomain and extraterminal inhibitor apabetalone reverses pathological epigenetic memory via the BRD4/P300/H3K27ac axis, thereby suppressing NLRP3 inflammasome‐mediated endothelial pyroptosis [64, 65] and restoring ABCA1‐dependent cholesterol metabolism [66]. These alterations are further reinforced at the posttranscriptional level through dysregulated noncoding RNA networks. Under diabetic stress, the downregulated expression of endothelial miR‐126 derepresses SPRED1, dampening MAPK signaling, and eNOS expression [67]. Furthermore, hyperglycemia depletes functional miR‐126 from endothelial microparticles (EMPs), abolishing their cell‐to‐cell reparative capacity, a defect directly confirmed in circulating microparticles of diabetic patients with vascular diseases [68].

Apart from metabolic insults, one of the main causes of ED is hemodynamic stress in the form of hypertension, which accelerates the development of AS. Hypertension‐driven ED is mediated through numerous synergistic pathways. Ang II selectively damages endothelium‐dependent dilation in coronary arteries and suppresses protective prostaglandin biosynthesis [69]. At the same time, organelle homeostasis is disturbed by hypertensive stress: impaired iron export via ABCB8 causes mitochondrial dysfunction, which initiates an iron‐dependent TGF‐β signaling axis that drives oxidative stress and inflammation [70]. Pressure overload activates proinflammatory adhesion molecules by upregulating expression of the endoplasmic reticulum protein Nogo‐B, facilitating ER–mitochondrial coupling via Mfn2 to increase calcium flux and ROS production [71].

### 2.2 Lipid Metabolism Reprogramming

#### 2.2.1 The Concept of Lipid Metabolism Reprogramming

The formation of foam cells is no longer regarded as a simple process of passive lipid overflow due to excessive lipoprotein uptake. It is now clear that plaque macrophages undergo reprogramming of intracellular cholesterol and fatty acid metabolism in response to local microenvironmental cues, thereby acquiring a persistent proinflammatory phenotype [72, 73]. These environmental cues are crucially translated into long‐term metabolic programs by epigenetic regulators. For example, the histone demethylase KDM2A stimulates the Src family kinase FYN, creating a signaling axis that favors sustained lipid accumulation and inflammation [74]; conversely, the dephosphorylation of the nuclear receptor LXRα causes macrophages to switch to an anti‐inflammatory state that enables cholesterol excretion [75]. Lipid metabolites themselves can act as powerful signaling molecules: oxysterol 25‐hydroxycholesterol increases inflammation and TLR4/NF‐κB activation [76]. However, some proresolving lipid mediators, such as resolvin T4, can increase cholesterol efflux through the SR‐BI scavenger receptor, thereby facilitating the resolution of inflammation [77]. Membrane‐level nanoscale organization is equally important: agonism of the receptor TREM2 improves cellular cholesterol handling and collagen deposition, which increases plaque stability [78]. Moreover, the formation of specific cholesterol‐rich membrane microdomains called “inflammarafts” in nonfoamy macrophages provides hyperinflammatory platforms for the assembly and activation of Toll‐like receptors [79]. This metabolic reprogramming may be reversible. For example, in aged mice, blocking the prostaglandin E2 (PGE_2_)–EP2 receptor axis can restore myeloid cell bioenergetics and even reverse age‐associated cognitive decline [80]. Systemic metabolic interference also shows promise, as evidenced by studies demonstrating that hepatocyte‐specific deletion of the nuclear receptor corepressor NCOR1 reroutes bile acid metabolism, increases fecal sterol excretion, and significantly reduces AS in mice [81]. These findings suggest an expanding therapeutic focus that includes reprogramming myeloid cell metabolism and function within plaques alongside lowering circulating lipids.

#### 2.2.2 Cholesterol Metabolism

Through a variety of scavenger receptors, macrophages internalize aggregated lipoprotein particles and modified LDL (SRA, CD36, SR‐BI, LOX‐1, and LRP1) [72]. In addition to receptor‐mediated endocytosis, Nox2‐derived ROS stimulate nonspecific, receptor‐independent fluid‐phase macropinocytosis under hypercholesterolemic conditions, which contributes to the formation of foam cells even in response to native LDL [82]. Posttranslational regulation of scavenger receptors further influences the extent of modified lipid uptake. In particular, Lys63‐linked polyubiquitination of SR‐BI is catalyzed by the E3 ubiquitin ligase RNF128, which prevents lysosomal degradation and promotes recycling to the plasma membrane, thereby maintaining ox‐LDL uptake and inflammatory activation [83]. Once internalized, lipid overload dramatically alters the metabolism and function of macrophages. Downregulation of miR‐369‐3p expression hinders this adaptation in diabetic conditions by derepressing its target, the succinate receptor GPR91. This leads to increased oxidative phosphorylation (OXPHOS), increased inflammasome‐driven inflammation, and worsened succinate signaling [84].

Unlike LDL, which has a well‐established proatherogenic role, high‐density lipoprotein (HDL) exerts context‐dependent effects and may lose its atheroprotective properties under pathological conditions. Importantly, its function can be positively regulated, as exemplified by hepatic ATF3, which increases HDL uptake via SR‐BI to promote reverse cholesterol transport and attenuate AS [85]. Paradoxically, high levels of HDL‐cholesterol (HDL‐C) are linked to increased atherosclerotic cardiovascular disease (ASCVD) risk [86]. This may be because, under such conditions, HDL is more susceptible to oxidative modifications, which severely compromise its antiatherogenic properties [86, 87].

#### 2.2.3 Fatty Acid Metabolism

Fatty acid metabolism is another critical component of metabolic reprogramming in AS. Fatty acid oxidation (FAO) plays a dual role in the disease process by regulating macrophage polarization and NLRP3 inflammasome activation, whereas adenosine 5′‐monophosphate‐activated protein kinase (AMPK) and peroxisome proliferator‐activated receptor (PPAR) signaling are the key nodes that regulate FAO [88]. For example, docosahexaenoic acid (DHA) promotes macrophage polarization toward an anti‐inflammatory M2 phenotype and increases lipid metabolism and cholesterol efflux by activating PPAR signaling, a mechanism consistent with the upregulation of PPARα expression by macrophage angiotensin‐converting enzyme (ACE), which further increases FAO [89, 90].

### 2.3 Inflammatory Cascades

#### 2.3.1 Innate Immunity

Plaque macrophages defy the traditional simple M1 (proinflammatory)/M2 (anti‐inflammatory/reparative) classification. High‐resolution single‐cell transcriptomic profiling has shown a continuous spectrum of distinct subtypes and states, including classical inflammatory cells that produce IL‐1β and TNF, TREM2^+^ lipid‐associated foam cells that, despite a homeostatic signature, can adopt proinflammatory roles through integration with signals such as GRN122, and newly identified populations such as apoptotic macrophages, SLAMF9^+^ macrophages, and FCGBP^+^ macrophages, with the latter two potentially being involved in tissue repair and containment functions [91, 92, 93, 94]. Functional validation is crucial, going beyond simple descriptive cataloging. Specifically, Lp(a)‐associated diacylglycerols promote macrophage priming via the TLR4/NF‐κB pathway and subsequent NLRP3 inflammasome activation, driving robust IL‐1β secretion. This dual‐signal mechanism correlates with the enrichment of distinct CD68^+^CASP1^+^IL‐1B^+^SELL^+^ inflammatory and CD68^+^IL‐18^+^TLR4^+^TREM2^+^ resident macrophage populations within human atherosclerotic plaques [95]. Concurrently, TLR4/MyD88/NF‐κB‐driven NLRP3 activation culminates in multiple forms of inflammatory cell death, including pyroptosis, apoptosis, and necroptosis [96]. Mendelian randomization, combined with direct analyses of human tissues, has identified macrophage‐derived lysosomal acid lipase (LIPA) as a potential causal driver of coronary artery disease (CAD) [97]. Dendritic cells (DCs) comprise several major subsets, including cDC1s, cDC2s, and pDCs, which are professional antigen‐presenting cells that take up arterial‐wall antigens (such as peptides derived from ApoB100) and are essential for initiating and directing the adaptive immune response. Different subsets of DCs frequently have conflicting effects on the course of disease [98]. Beyond macrophages and DCs, neutrophils actively contribute to plaque progression through the formation of neutrophil extracellular traps (NETs). Increasing evidence indicates that NETs function as potent amplifiers of vascular inflammation [99]. Mechanistically, cholesterol‐loaded macrophages promote NET formation through NLRP3 inflammasome‐dependent IL‐1β secretion, establishing a pathogenic macrophage–neutrophil inflammatory circuit [100]. NET‐associated histones further facilitate monocyte adhesion and accumulation within the arterial wall, thereby accelerating lesion growth [101]. In addition, NET formation is associated with increased local IL‐1β production and inflammatory cell recruitment within plaques, contributing to lesion progression [102]. Innate immune cells can also develop a de facto memory response known as “trained immunity” [103]. Rather than representing a transient activation, this process is maintained through coordinated metabolic and epigenetic reprogramming of hematopoietic progenitors and their myeloid descendants. In diabetes, transient hyperglycemia induces persistent chromatin remodeling and Runx1‐dependent inflammatory priming that remains detectable even after normalization of glucose levels, providing a mechanistic basis for metabolic memory and sustained vascular inflammation [104]. Likewise, ox‐LDL promotes trained immunity through metabolic rewiring, including increased mitochondrial oxidative phosphorylation, which drives long‐term inflammatory responsiveness [105]. Mechanistically, the oxysterol receptor LXRα links these metabolic alterations to epigenetic remodeling by regulating the activation of histone marks at inflammatory gene loci [106]. These findings highlight trained immunity as a central mechanism connecting metabolic stress, epigenetic memory, and persistent vascular inflammation in AS.

#### 2.3.2 Adaptive Immunity

T and B lymphocytes both play complex, precisely calibrated roles in the regulation of AS (Figure 3). Helper T type 1 cells (Th1s) and type 17 cells (Th17s) primarily have a proatherosclerotic effect, and some B2 cell‐derived antibodies are also similar according to well‐established experimental mouse models. Th2s, regulatory T cells (Tregs), and other innate B1 cells that produce natural IgM antibodies typically provide protection. Follicular helper T cells (Tfhs) located in lymphoid organs coordinate the antibody class switching of B cells and affect the quality of the humoral response [107]. However, cross‐species comparative single‐cell RNA‐seq analyses highlight significant distinctions between human disease and mouse models. Human plaques are rich in T cells, display clear signatures of IFN‐γ production and T‐cell exhaustion (a state of dysfunction in chronic antigen exposure), and exhibit persistent clonal expansion of CD4^+^ T cells that are specific for ApoB (the core protein of LDL) but resist neat classification into classical Th1, Th2, or Th17 subsets [108, 109]. Recent single‐cell T‐cell receptor sequencing (scTCR‐seq) has characterized this clonal expansion as a breakdown of peripheral tolerance, supporting the concept that autoimmune mechanisms contribute substantially to AS progression [19, 110]. As a prime example of this plasticity, seminal studies identifying specific ApoB epitopes (such as p18) reveal that these antigens primarily induce protective Foxp3^+^ regulatory T cells in healthy individuals [111]. However, these ApoB‐reactive CD4^+^ T cells progressively convert from initially protective Tregs to pathogenic, mixed‐phenotype effector cells during disease progression [112]. This pathogenic transition is further exemplified by the emergence of an unusual subset of CCR5^+^T‐bet^+^Foxp3^+^ effector T cells, which utilize CCR5 to home specifically to the atherosclerotic aorta and drive AS rather than act as suppressors [113]. Along this line, the humoral response is correspondingly engaged, as anti‐ApoB autoantibodies exhibit distinct IgG‐ and IgM‐associated immune signatures, further supporting the autoimmune nature of AS [114]. Furthermore, this localized autoimmune response is amplified by molecular mimicry, in which expanded plaque TCRs cross‐react with both viral epitopes and resident vascular self‐antigens [115]. It is also believed that local cDC1s in plaques increase this autoinflammatory reaction by activating Th1 and CD8 T cells in a STING‐dependent manner [116]. The subsequent recruitment of these diverse immune cells into the inflamed vasculature is actively governed by cell type‐specific mechanisms; the CD40–CD40L axis has recently been demonstrated to drive this process, with endothelial‐expressed CD40L serving as a critical proatherogenic adhesion receptor for leukocytes [117]. Moreover, remote systemic events such as myocardial infarction (MI) can trigger the release of alarmins (e.g., IL‐1α and MRP8/14), activating B cells to generate pathogenic IgG antibodies that target neoantigens in the plaque [118]. Protective immunity can be dampened by the plaque microenvironment itself. For example, dysregulated glycolytic metabolism in human plaques impairs the function of infiltrating naïve CD4^+^ T cells, limiting their ability to mount effective regulatory responses [119]. Whether these human‐specific immune features reflect fundamental biological differences or simply differences in disease stage between human studies and experimental models remains an important question.

**FIGURE 3:**
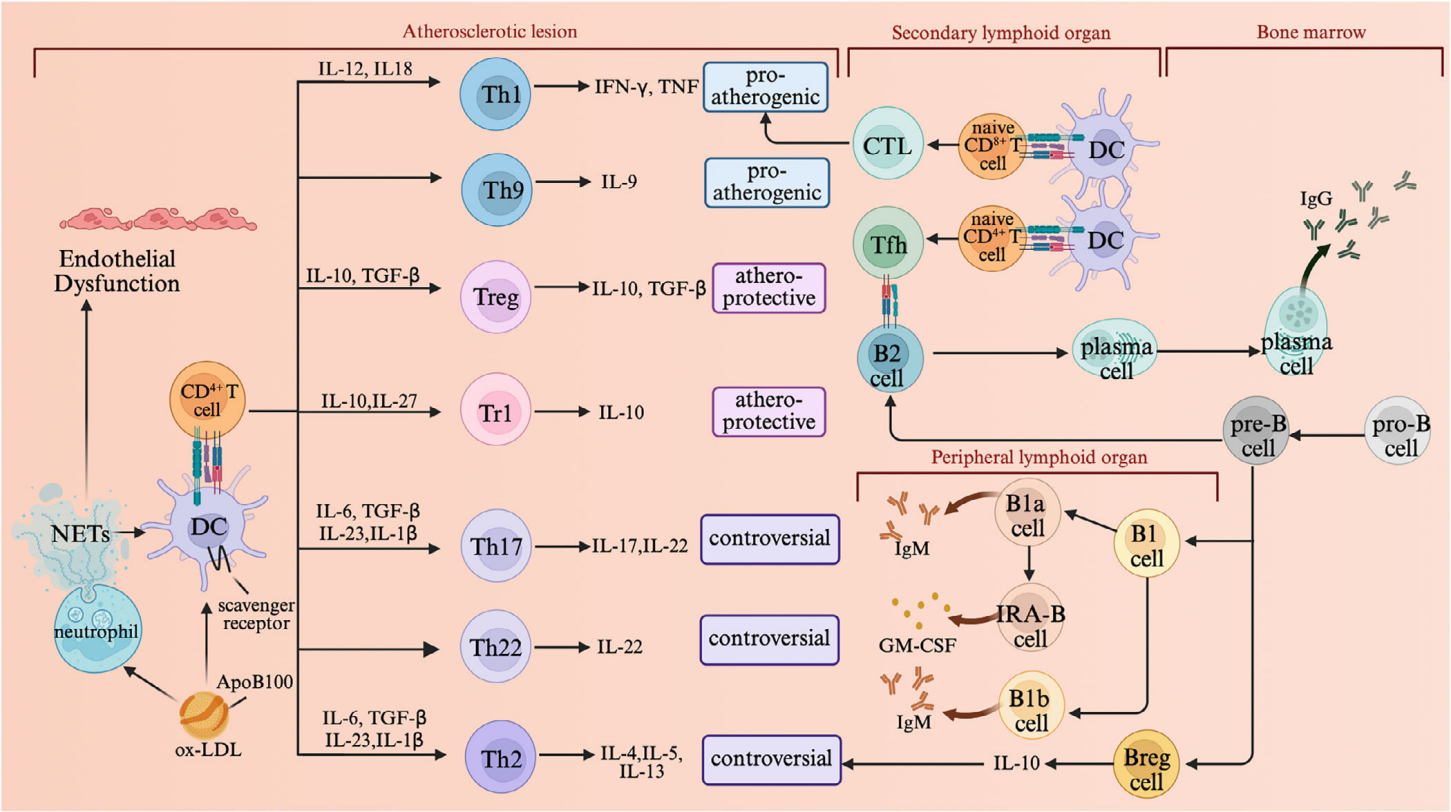
Crosstalk between innate and adaptive immune components in the pathogenesis of atherosclerosis. In atherosclerotic lesions, ox‐LDL activates neutrophils and promotes NET formation, thereby aggravating endothelial dysfunction and facilitating dendritic cell (DC) activation. DC‐mediated antigen presentation drives CD4^+^ T‐cell differentiation into proatherogenic (Th1 and Th9), atheroprotective (Treg and Tr1), or context‐dependent (Th17, Th22, and Th2) subsets. In secondary and peripheral lymphoid organs and the bone marrow, DCs coordinate cytotoxic T lymphocyte and Tfh responses and regulate B‐cell maturation. B1 cells produce IgM, IRA‐B cells secrete GM‐CSF, and Breg cells exert controversial effects by producing IL‐10.

### 2.4 Vascular Smooth Muscle Cell Plasticity

Recent advances in single‐cell and chromatin accessibility profiling have transformed our understanding of VSMC plasticity. The field has moved beyond isolated markers to explore complex spatiotemporal trajectories. The single‐nucleus assay for transposase‐accessible chromatin with sequencing (snATAC‐seq) across human coronary arteries has recently mapped over 320,000 accessible sites, identifying critical regulatory elements specifically within smooth muscle cell (SMC) transition states, such as fibromyocytes [120]. Concurrently, bioinformatic deconvolution of bulk microarrays using plaque scRNA‐seq data has highlighted multiple mesenchymal cell populations in atherosclerotic lesions, including distinct classical and modulated SMC subphenotypes [121]. These technologies reveal that the transition of VSMCs involves not only a loss of the contractile state but also a hyperproliferative process. Indeed, lineage tracing has demonstrated that SMC‐derived cells in AS exhibit tumor cell‐like characteristics, including genomic instability, evasion of senescence, and hyperproliferation, a phenotypic switch that can be accelerated by oncogenic mutations such as KrasG12D [21].

These lineage transitions are orchestrated by a multilayered epigenetic regulatory network. Traditionally, the protein arginine methyltransferase PRMT1 helps epigenetically maintain myocardin expression and the differentiated, contractile state [122]. However, under pathogenic stress, multilayered chromatin remodeling overrides this baseline. Recent single‐nucleus profiling has suggested PRDM16 as a key transcription factor driver in this process [120]. PRDM16 functions as a critical transcriptional repressor of the synthetic SMC phenotype by occupying chromatin and suppressing activating marks at synthetic loci; its deletion induces a synthetic program across all SMC subtypes [123]. Conversely, the histone methyltransferase SUV39H1 actively drives dedifferentiation by regulating dynamic trimethylation at histone H3 lysine 9 (H3K9me3), which alters chromatin accessibility and promotes the expression of KLF4 [124]. Furthermore, epigenetic regulation extends to noncoding regions, where the human‐specific long noncoding RNA INKILN physically interacts with and stabilizes MKL1 and USP10 to activate a proinflammatory gene program in VSMCs [125].

Ultimately, VSMCs differentiate into distinct phenotypes with opposing consequences for plaque fate through intricate microenvironmental crosstalk (Table 1). A classic two‐step conversion is triggered by high‐lipid stress and inactivation of the NOTCH pathway. First, VSMCs adopt a dedifferentiated “modulated” state, and subsequently, phagocytic, macrophage‐like cells that contribute to the necrotic core [126]. Recent single‐cell transcriptome data from human plaques corroborate this finding, showing that expression of the ChemR23 receptor is highest in contractile VSMCs but markedly reduced in synthetic ones and that its deficiency actively promotes the switching of VSMCs into macrophage‐like foam cells [127]. The transcription factor HOXA1 has been shown to accelerate this adverse change by upregulating expression of KLF4 and increasing NF‐κB p65 signaling [126, 128]. In contrast, cells in intermediate differentiation states attempt to communicate with and stabilize the matrix; for example, the matricellular protein CCN2 can reduce ER stress and lipid uptake, promoting a more stable plaque phenotype [129]. Additionally, novel regulators, such as the circadian rhythm gene ARNTL, have been identified through transcriptomics; silencing ARNTL in human carotid SMCs attenuates proliferation and increases the expression of contractile markers [121]. As a result, plaque vulnerability is dictated by the dynamic equilibrium between these diverse, epigenetically driven fates.

**TABLE 1:** Major phenotypic states of vascular smooth muscle cells in recent atherosclerosis studies.

VSMC phenotypic state	Related molecules	Experimental model/species	References
Senescent VSMC	CDKN2A/p16, ICAM1, TMEM178B, and SFRP4	Mouse: VSMCs and plaques; Human: Plaques.	[301]
	SIRT6	Mouse: VSMC‐specific SIRT6‐overexpressing Apoe^−/−^ mice; Human: Aortic and plaque‐derived VSMCs; aortic and carotid plaques.	[302]
Synthetic VSMC	Socs1, miR‐155‐5p	Mouse: STZ‐induced diabetic Apoe^−/−^ mice; cultured mouse VSMCs under diabetic‐like conditions.	[303]
Fibroblast‐like VSMC	BMAL1, YAP1, FSP1	Mouse: VSMC‐specific Bmal1‐deficient atherosclerotic mice; Human: Stable and unstable human atherosclerotic plaques.	[304]
	CEMIP, FN1	Mouse: Apoe^−/−^ mice; Human: carotid plaques.	[13]
	KIF13B	Mouse: Ldlr^−/−^ and Ldlr^−/−^Kif13b^−/−^ mice; VSMC‐specific Kif13b‐knockout mice; Human: Advanced human atherosclerotic plaques and primary human aortic smooth muscle cells.	[305]
	NOTCH3, PAR1	Mouse: Atherosclerosis and acute vascular injury model; Human: Atherosclerotic plaques.	[306]
Macrophage‐like/foam‐cell‐like VSMC	ChemR23	Mouse: Nonhematopoietic ChemR23‐deficient Apoe^−/−^ mice; Human: Aortic SMCs; advanced human atherosclerotic plaques.	[127]
	NOTCH, MYOCD	Mouse: Atherosclerotic plaques; Rat: Primary rat VSMCs.	[126]
	PAD4, H3Cit	Mouse: SMC‐lineage‐tracing mice and SMC‐specific Pad4‐deficient mice; Human: Arterial atherosclerotic plaque.	[307]
	BHLHE40	Mouse: SMC‐lineage‐tracing atherosclerotic mice; Human: Carotid atherosclerotic tissues.	[308]
Tumor‐like VSMC	−	Mouse: SMC‐lineage‐tracing and SMC‐specific Kras^G12D atherosclerosis models; Human: Atherosclerotic tissue samples.	[21]
Osteochondrogenic VSMC	TXNIP	Mouse: Txnip^−/−^ mice and Txnip^flox/flox^ mice; Human: Calcified atherosclerotic plaques, primary aortic VSMCs.	[309]
	NF90, AGER1	Mouse: Diabetic atherosclerosis model with VSMC‐specific NF90/110 deletion; Human: Calcified atherosclerotic plaques from patients with diabetes; primary human aortic VSMCs.	[310]
Mesenchymal cell‐like VSMC	RUNX1, TIMP1	Mouse: Myh11‐lineage‐traced carotid injury model; Apoe^−/−^ lineage‐traced atherosclerosis model; Human: Carotid atherosclerotic plaques, nonplaque aortas, and cultured human VSMCs.	[311]
	SCA1	Mouse: VSMC‐lineage‐traced atherosclerosis and vascular injury models.	[312]

Abbreviations: Apoe, apolipoprotein E; H3Cit, citrullinated histone H3; Ldlr, low‐density lipoprotein receptor; miR, microRNA; PAD4, peptidylarginine deiminase 4; SMC, smooth muscle cell; STZ, streptozotocin; VSMC, vascular smooth muscle cell.

Gene and protein symbols follow standard mouse and human nomenclature.

### 2.5 Microbiome–Vascular Axis

An increasing body of literature indicates that the gut and oral microbiomes serve as a “microbiome–vascular axis” in AS. Dysbiosis of the gut, characterized by an increased abundance of oral‐associated or even pathogenic bacteria (e.g., *Streptococcus* and *Enterobacteriaceae*) and a low abundance of beneficial short‐chain fatty acid‐producing genera (e.g., *Bacteroides*), is commonly observed in patients with CVDs. These microbes produce proatherogenic metabolites, most notably trimethylamine‐N‐oxide (TMAO), from dietary choline and L‐carnitine, as well as endotoxins such as lipopolysaccharide (LPS). They also systemically modulate host gene expression programs related to oxidative stress and DNA repair [130, 131]. These microbial metabolites and endotoxins promote ED, activate the NLRP3 inflammasome and NF‐κB signaling in macrophages, and increase platelet reactivity and thrombotic risk [132, 133, 134, 135, 136]. The oral microbiome may also influence the microbiome–vascular axis independently of the gut microbiome. Oral bacterial species are enriched in the gut microbiota of patients with CVD. In particular, the periodontal pathogen *Porphyromonas gingivalis* may increase plasma TMAO levels by upregulating the expression of hepatic flavin‐containing monooxygenase 3 (FMO3) without necessarily altering gut microbial trimethylamine (TMA) production. These findings support the existence of an oral–liver–TMAO pathway that links oral health to systemic cardiovascular risk [137, 138]. Nonetheless, the pathogenic effect of this loop appears to be quantitative rather than absolute. For example, in a large prospective cohort of vegetarian adults with chronically low TMAO levels, Tong et al. [139] reported a significant incidence of ischemic heart disease over 18 years of follow‐up, with a hazard ratio (HR) of 0.78 compared with that of the meat eaters. This implies that even with an attenuation of the TMAO axis, there is an approximately 22% reduction in relative risk. These findings support three conclusions: (i) Platelet priming, among other biological activities, can be preserved even when patients are exposed to very little TMAO; (ii) This considerable remaining risk may be attributable to TMAO‐independent mechanisms, including Lp(a), clonal hematopoiesis, and vascular calcification, making interventions against TMAO additive but not curative; and (iii) TMAO is not a binary trigger but rather a continuous risk rheostat. Combined targeting of microbial TMA formation and these orthogonal pathways will thus probably be needed to minimize residual ASCVD risk.

## 3 Therapeutic Interventions

Actively treating various proatherosclerotic conditions is an important strategy for preventing ASCVD events. For example, intensive lipid lowering, strict blood pressure control, and glycemic control can inhibit the progression of AS. Beyond diagnosing and treating the clinical manifestations of AS, clinicians must also manage the diseases and risk factors that promote AS, including lipid management, inflammation control, and immune modulation.

### 3.1 Current Therapeutic Interventions

#### 3.1.1 Lifestyle Interventions

Lifestyle interventions are the cornerstone of both primary and secondary prevention of AS, a point emphasized by all major guidelines. Although their importance is widely recognized, their actual implementation still requires improvement [140].

##### 3.1.1.1 Dietary Modification

Improving dietary habits is among the key nonpharmacological methods for preventing and managing CVD [140]. Studies emphasize reducing the intake of saturated fats and sodium while increasing the consumption of unsaturated fats, dietary fiber, fruits, vegetables, and whole grains [141]. In accordance with the American Heart Association's (AHA) “Life's Essential 8” guidelines, diet modification is among the key metrics for lowering ASCVD risk [142].

##### 3.1.1.2 Physical Activity

Physical activity is essential for cardiovascular health [143]. It is recommended to engage in at least 150 min of moderate‐intensity aerobic exercise per week, such as brisk walking, jogging, or swimming, and to incorporate strength training. Regular physical activity can improve cardiopulmonary function, aid in weight management, and positively impact blood pressure control [144]. Studies have shown that both aerobic exercise and strength training are effective at supporting cardiovascular risk‐factor management and are associated with a lower risk of all‐cause mortality [143].

##### 3.1.1.3 Smoking Cessation and Alcohol Restriction

Smoking is an important and modifiable risk factor for AS. AHA guidelines emphasize smoking cessation as one of the key lifestyle metrics for reducing ASCVD risk. In addition, alcohol intake should be limited, as excessive consumption is associated with increased cardiovascular risk [140].

##### 3.1.1.4 Stress Management

Chronic stress is one of the risk factors for CVD. Relaxation techniques such as meditation, yoga, and deep breathing can effectively alleviate mental stress, thereby maintaining a positive mindset. These psychological and social interventions are considered to be closely related to cardiovascular protection [140].

#### 3.1.2 Pharmacological Treatment

Pharmaceuticals play important roles in managing AS, controlling risk factors, maintaining plaque stability, and preventing major cardiovascular events (Table S1).

##### 3.1.2.1 Lipid‐Lowering Drugs

###### 3.1.2.1.1 Statins

Statins are the cornerstone drugs for lowering LDL‐C levels, as they inhibit HMG‐CoA reductase to reduce cholesterol synthesis. They not only lower LDL‐C but also stabilize atherosclerotic plaques and exert anti‐inflammatory effects [145].

###### 3.1.2.1.2 Cholesterol Absorption Inhibitors

Inhibitors such as ezetimibe lower LDL‐C by inhibiting intestinal cholesterol absorption and are often used in combination with statins, especially for patients who cannot achieve LDL‐C targets with statin monotherapy [146].

###### 3.1.2.1.3 Monoclonal Antibodies (mAbs) Against PCSK9

These drugs lower LDL‐C levels by inhibiting the ability of the PCSK9 protein to degrade LDL receptors, thereby increasing hepatic clearance of LDL‐C. They can not only effectively reduce LDL‐C levels by 50%–60% on the basis of statins and improve the prognosis of ASCVD patients [145] but also show benefits in primary prevention in high‐risk groups without a history of MI or stroke [147].

###### 3.1.2.1.4 Small Interfering RNA (siRNA) Targeting PCSK9

The ORION clinical trial found a significant reduction in LDL‐C (50%) by inclisiran in patients with atherosclerotic CVD [148]. In addition, one study showed that after 3 months of inclisiran therapy, patients at high cardiovascular risk experienced significant reductions in LDL‐C levels and key inflammatory markers (PTX3 and IL‐18), suggesting that the drug has anti‐inflammatory effects beyond its lipid‐lowering effects [149]. A recent study evaluated the effect of inclisiran on plaque delipidation at the European Society of Cardiology (ESC) 2025 conference and found that maximum lipid‐core burden index at 4 mm was reduced in the inclisiran group from 193.7 to 103.2, a decrease of 46.7% (−90.5, *p* = 0.017) at the 15‐month follow‐up [150]. These findings indicate that inclisiran has the potential to stabilize plaques, and we look forward to more clinical studies to confirm these findings.

###### 3.1.2.1.5 A Third‐Generation Small Binding Protein Targeting PCSK9

Lerodalcibep is a novel, third‐generation PCSK9 inhibitor based on an adnectin scaffold—a small binding protein engineered to bind with high affinity to human PCSK9. In one randomized, double‐blind, placebo‐controlled Phase 3 trial, 922 patients with established CVD or at very high or high CVD risk and LDL‐C ≥ 70 mg/dL (CVD) or ≥ 100 mg/dL (high risk) despite maximally tolerated statin therapy were randomized 2:1 to receive monthly subcutaneous lerodalcibep (300 mg) or placebo for 52 weeks. The percent change in LDL‐C at Week 52 showed a placebo‐adjusted LDL‐C reduction of 56.2%; 90% of lerodalcibep‐treated patients achieved ≥ 50% LDL‐C lowering and guideline‐recommended targets. Treatment‐emergent adverse events were similar between groups except for mild/moderate injection‐site reactions (6.9% vs. 0.3%). Therefore, monthly administration of lerodalcibep significantly reduced LDL‐C levels in high‐risk CVD patients, with a safety profile similar to that of placebo, supporting its long‐term use when statins alone are insufficient [151].

###### 3.1.2.1.6 mAbs Against Angiopoietin‐Like Protein 3 (ANGPTL3)

ANGPTL3, a protein predominantly synthesized by hepatocytes and secreted into the bloodstream, plays a key role in lipid metabolism by suppressing lipoprotein lipase and endothelial lipase activity, which in turn impedes the catabolism of triglyceride (TG)‐rich lipoproteins. Conversely, inherited deficiency variants of ANGPTL3 correlate with reduced plasma TG and LDL‐C concentrations, thereby lowering susceptibility to ASCVD [152]. Evinacumab is a human‐derived mAb specifically designed to target circulating ANGPTL3 protein. Clinical trials have shown that evinacumab significantly reduced LDL‐C levels by 42% from baseline at Week 24 in patients with homozygous familial hypercholesterolemia (HoFH) [153] and significantly reduced LDL‐C levels by up to 56.0% at Week 16 in patients with refractory hypercholesterolemia [154]. In addition, evinacumab significantly reduced cardiovascular events over 4 years of follow‐up in patients with HoFH [155]. Recently, another mAb against ANGPTL3, SHR‐1918, resulted in a decrease in LDL‐C of 21.7%–29.9% among patients with moderate‐to‐high ASCVD risk in a Phase 2 study [156]. Furthermore, in a Phase 1 trial involving 48 adults with mixed hyperlipidemia, LY3475766—a human mAb specifically blocking ANGPTL3/8—was well tolerated and dose‐dependently reduced TG (up to −70%), remnant cholesterol (−86%), and LDL‐C (−32%) while increasing HDL‐C (+27%) levels [157].

###### 3.1.2.1.7 mAbs Against Angiopoietin‐Like Protein 4 (ANGPTL4)

ANGPTL4 is a key regulator of TG metabolism; individuals carrying loss‐of‐function mutations in this gene exhibit lower levels of circulating TG and remnant cholesterol, along with decreased susceptibility to both type 2 diabetes mellitus (T2DM) and ASCVD. A clinical investigation of MAR001, a mAb designed to inhibit ANGPTL4, was conducted through two separate early‐phase trials: a first‐in‐human, placebo‐controlled, single‐ascending‐dose Phase 1 study involving healthy volunteers and participants with hypertriglyceridemia, followed by a randomized, double‐blind, placebo‐controlled Phase 1b/2a trial that included 55 individuals with hypertriglyceridemia and metabolic dysfunction. These studies revealed that MAR001 administration was safe and generally well tolerated, with no observed treatment‐emergent systemic inflammation or alterations in mesenteric lymph node morphology as assessed by MRI; moreover, the 450 mg dose produced placebo‐adjusted mean reductions of 52.7% in TG levels and 52.5% in remnant cholesterol levels at Week 12 [158]. These results suggest that ANGPTL4 inhibition may attenuate atherosclerotic processes and thereby reduce the incidence of ASCVD events.

###### 3.1.2.1.8 Targeting the Scavenger Receptor for ox‐LDL

Studies have shown that lectin‐like oxidized LDL receptor‐1 (LOX‐1) can promote AS and inflammation. A randomized, double‐blind, dose‐finding study showed that a LOX‐1 antagonist (MEDI6570) can significantly reduce soluble LOX‐1 and IL‐6 levels but did not reduce noncalcified coronary plaque volume in patients with residual inflammation after acute MI [159].

###### 3.1.2.1.9 Bempedoic Acid

Bempedoic acid is an inhibitor of ATP citrate lyase (ACLY), an enzyme that plays a key role in lipid synthesis. The CLEAR Outcomes trial demonstrated that bempedoic acid can safely and effectively reduce MACEs in 13,970 statin‐intolerant patients [160], as well as in high‐risk primary prevention patients [161].

###### 3.1.2.1.10 Antisense Oligonucleotides (ASOs) Targeting Apolipoprotein (Apo) C‐III

Volanesorsen and olezarsen are two ASOs targeting ApoC‐III. Volanesorsen resulted in a 73.9% reduction in TG levels after 3 months of treatment [162] and the maintenance of 50% lower levels compared with those of the control over 15 months [163] and significantly reduced hepatic steatosis in patients with familial partial lipodystrophy [164]. Like volanesorsen, olezarsen, an N‐acetylgalactosamine‐conjugated antisense oligonucleotide, has also been shown to significantly reduce TG levels by 50% at 6 months in patients with high cardiovascular risk [165] and by more than 50% at 6 months in patients with moderate hypertriglyceridemia [166]. Whether this magnitude of triglyceride lowering translates into atherosclerotic benefit, however, remained unresolved. Recently, the ESSENCE‐TIMI 73b trial evaluated olezarsen in patients with hypertriglyceridemia at high cardiovascular risk. Despite reducing triglycerides by ∼64% and remnant cholesterol by ∼72%, olezarsen did not reduce noncalcified coronary plaque volume over 12 months compared with placebo (placebo‐adjusted least‐squares mean difference, 2.98% [95% CI, −3.4 to 9.3]; *p* = 0.36) [167].

##### 3.1.2.2 Antidiabetic Therapies

###### 3.1.2.2.1 Biguanides

Metformin is a foundational drug for the treatment of T2DM, and its clinical value has long surpassed the scope of glycemic control. Its antiatherosclerotic effects are primarily achieved through the activation of AMPK [168]. As an intracellular energy sensor, AMPK is activated under metabolic stress, subsequently regulating a series of downstream signaling pathways to maintain vascular health [169]. Metformin protects against AS through a multitarget approach. It improves endothelial function by increasing NO production, which helps blood vessels relax and heal [170, 171]. Additionally, it reduces inflammation by blocking key inflammatory pathways and preventing immune cells from building up inside vessel walls, effectively preventing early plaque formation [170, 172]. Finally, metformin decreases oxidative stress by decreasing the levels of harmful ROS and promoting the clearance of damaged cell parts (autophagy), ultimately keeping blood vessel cells healthy under stress [168, 171]. With respect to clinical evidence, the UKPDS study was the first study to confirm that metformin reduces the risk of macrovascular complications. In the metformin group (maximum dosage of 2550 mg/day), the risk of macrovascular disease was 30% lower than that in the conventional treatment group. The risk of MI was reduced by an even greater 39%. During the subsequent 10‐year posttrial follow‐up, this reduction in the risk of MI and macrovascular endpoints continued [173, 174].

###### 3.1.2.2.2 Sodium‐Glucose Cotransporter 2 Inhibitors (SGLT2is)

These inhibitors were initially designed as purely glucose‐lowering medications that promote urinary glucose excretion by inhibiting the reabsorption of glucose and sodium in the proximal convoluted tubule of the kidney. However, clinical trials have found that their efficacy in slowing AS progression exceeds what would be expected from their diuretic and hypoglycemic effects alone [175]. Studies utilizing optical coherence tomography (OCT) and coronary computed tomography angiography (CCTA) have directly demonstrated the remodeling of plaque morphology. For patients undergoing percutaneous coronary intervention (PCI), long‐term administration of SGLT2is promoted the transition of coronary plaques toward a stable phenotype. This was characterized by a significant increase in fibrous cap thickness (48 ± 15 µm vs. 26 ± 24 µm in nonusers, *p* = 0.005) and a reduced lipid arc [176]. Furthermore, longitudinal CCTA follow‐up cohorts indicated that SGLT2i therapy significantly reversed both the total coronary plaque volume and the percent atheroma volume (PAV) [177].

###### 3.1.2.2.3 Pioglitazone

Belonging to the thiazolidinedione (TZD) class of medications, pioglitazone functions as a synthetic ligand for peroxisome proliferator‐activated receptor‐gamma (PPAR‐γ). PPAR‐γ is a nuclear hormone receptor that critically regulates carbohydrate and lipid metabolism, as well as inflammatory responses in vascular tissues [178]. Beyond its role as a classic insulin sensitizer, pioglitazone effectively counteracts ox‐LDL‐driven intracellular lipid retention by upregulating the expression of the cholesterol efflux transporters ABCA1 and ABCG1 in macrophages. By doing so, it locally inhibits the formation of atherosclerotic plaques within the vascular wall [179]. The PERISCOPE trial enrolled 543 patients with T2DM and angiographically confirmed CAD who were randomly assigned to receive either pioglitazone or sulfonylurea glimepiride for 18 months [180]. Intravascular ultrasound (IVUS) findings showed contrasting changes in atherosclerotic burden between the two groups. The change in PAV, which served as the primary efficacy endpoint, significantly increased by 0.73% in the glimepiride group but slightly decreased by 0.16% in the pioglitazone group [180].

###### 3.1.2.2.4 Glucagon‐Like Peptide‐1 Receptor Agonists (GLP‐1RAs)

These drugs were initially developed as glucose‐lowering agents that stimulate insulin secretion while suppressing glucagon release. However, large‐scale cardiovascular outcome trials (CVOTs) have suggested that their efficacy in reducing atherothrombotic events may not be attributable solely to their glycemic effects. The landmark SELECT trial enrolled 17,604 patients with established ASCVD who had overweight/obese but not diabetes, and demonstrated that, compared with placebo, administration of once‐weekly semaglutide (2.4 mg) reduced MACEs risk by 20% over a median follow‐up of 3.3 years (HR: 0.80; 95% CI, 0.72–0.90) [181]. Moreover, the SUSTAIN‐6 trial found that compared with placebo, once‐weekly semaglutide (0.5 mg and 1.0 mg) significantly reduced MACEs in T2DM patients at high cardiovascular risk (HR: 0.74; 95% CI, 0.58–0.95) [182]. The LEADER trial further corroborated these findings, revealing that liraglutide reduced MACEs by 13% (HR: 0.87; 95% CI, 0.78–0.97) in 9340 T2DM patients at high cardiovascular risk [183]. Current guidelines, including the 2023 ESC guidelines and the 2026 American Diabetes Association (ADA) Standards of Care, recommend GLP‐1RAs with proven cardiovascular benefit for patients with T2DM and established ASCVD as part of comprehensive cardiovascular risk reduction [184, 185]. Imaging studies have directly revealed the plaque‐modifying effects of GLP‐1RAs, with serial CCTA showing significant regression of nonculprit lesion plaque burden and PAV (PAV: −6.1% vs. −0.7%, *p* = 0.039) in GLP‐1RA‐treated diabetic patients with recent acute coronary syndrome (ACS) [186], indicating that cardiovascular protection is through direct antiatherosclerotic, anti‐inflammatory, and endothelial effects.

###### 3.1.2.2.5 Dipeptidyl Peptidase‐4 Inhibitors (DPP‐4is)

DPP‐4is prolong endogenous incretin signaling by preventing the degradation of glucagon‐like peptide‐1 and glucose‐dependent insulinotropic polypeptide, thereby increasing glucose‐dependent insulin secretion and suppressing glucagon release with a low risk of hypoglycemia and a largely weight‐neutral profile. Experimental and translational studies suggest that DPP‐4 inhibition may attenuate AS by reducing monocyte recruitment, vascular inflammation, oxidative stress, and ED [187]. However, these potentially favorable vascular effects have not translated into consistent reductions in clinical atherothrombotic events. In major CVOTs, DPP‐4is were generally noninferior for MACEs, but saxagliptin was associated with an increased risk of hospitalization for heart failure [188, 189, 190, 191, 192]. Therefore, DPP‐4is should be viewed mainly as glucose‐lowering agents with cardiovascular safety rather than as an established antiatherosclerotic therapy [184].

##### 3.1.2.3 Antihypertensive Therapies

###### 3.1.2.3.1 Calcium Channel Blockers (CCBs)

Third‐generation dihydropyridines such as amlodipine and lacidipine selectively inhibit the inward flux of calcium ions across the membranes of VSMCs and cardiomyocytes, thereby reducing peripheral vascular resistance and inducing potent vasodilation. However, their unique anti‐AS advantages are largely derived from their distinctive physicochemical properties. Their high lipophilicity enables them to integrate deeply into the lipid bilayer of the cell membrane, where they exert powerful membrane antioxidant effects. This effectively protects endothelial cells from oxidative stress‐induced damage and lipid peroxidation [193]. In the CAMELOT trial initiated in 1999, 1997 patients with angiographically documented CAD and normal blood pressure were randomized to receive amlodipine, enalapril, or placebo. Amlodipine reduced the incidence of adverse cardiovascular events compared with placebo (16.6% vs. 23.1%, *p* = 0.003). In the IVUS substudy of 274 patients, PAV increased in the placebo group (1.3 ± 4.4%, *p* = 0.001 vs. baseline), whereas the change with amlodipine was not significant (*p* = 0.31 vs. baseline). Among patients whose baseline systolic blood pressure exceeded the mean, PAV progression was slower with amlodipine than with placebo (0.2 ± 3.9% vs. 2.3 ± 4.7%, *p* = 0.02), with no effect below the mean [194]. The PREVENT study showed that while amlodipine did not alter the angiographic progression of early coronary luminal diameter narrowing, high‐resolution B‐mode ultrasonography revealed a remodeling effect within the carotid artery system. In the placebo group, the carotid intima‐media thickness (CIMT) increased by 0.033 mm; conversely, the amlodipine group exhibited a regression of 0.0126 mm (*p* = 0.007). These findings indicate a substantial reversal in the progression of carotid AS [195].

###### 3.1.2.3.2 ACE Inhibitors

The role of ACE inhibitors in the management of atherosclerotic vascular disease was established by the landmark HOPE study. This trial randomized 9297 high‐risk patients—excluding those with heart failure or severe left ventricular dysfunction—to receive either 10 mg of ramipril daily or a placebo [196]. The SECURE trial, a substudy of the HOPE study, further evaluated 732 patients using highly reproducible B‐mode ultrasonography to measure 12 carotid artery segments. The results found a clear dose‐dependent inhibition of the progression slope in maximum CIMT. The annual progression rate was 0.0217 mm in the placebo group, which decreased to 0.0180 mm in the 2.5 mg ramipril group and reached a nadir of 0.0137 mm in the 10 mg ramipril group (overall intervention effect *p* = 0.033) [197].

#### 3.1.3 Revascularization Strategies

When pharmacological treatment is ineffective or vascular stenosis is severe, percutaneous interventions and surgical revascularization become important treatment options.

##### 3.1.3.1 Percutaneous Interventions

###### 3.1.3.1.1 Interventional Therapy for Coronary Artery Stenosis

This procedure restores blood flow by placing stents or balloons to dilate narrowed coronary arteries. Drug‐eluting stents are a commonly used treatment method, but there is still room for improvement in their coating biocompatibility and drug‐loading capacity. Self‐assembly coating technology offers a thinner, stronger bonding, and more biocompatible alternative for stents [198]. Recent studies on novel bioresorbable scaffolds, including an iron‐based scaffold (IRONMAN II) [199] and a poly‐L‐lactic acid‐based scaffold (FUTURE‐III) [200], have shown noninferiority to contemporary drug‐eluting stents in key efficacy endpoints, with a very low incidence of scaffold thrombosis and promising long‐term outcomes.

###### 3.1.3.1.2 Peripheral Endovascular Intervention

This procedure restores blood flow through percutaneous transluminal angioplasty and/or stent placement across various arterial territories. In the carotid artery, the current evidence base for carotid artery stenting (CAS) demonstrates significant context‐dependent heterogeneity. In asymptomatic patients, a meta‐analysis of three modern RCTs (including CREST‐2, SPACE‐2, and ECST‐2) found no significant benefit of CAS over contemporary medical management alone (RR: 0.72; 95% CI, 0.23–2.27) [201]. In symptomatic carotid stenosis, network meta‐analyses have demonstrated that compared with carotid endarterectomy (CEA), CAS is associated with lower risks of cranial nerve injury, hematoma, and infection [202]. Current ESC guidelines recommend CEA for selected patients with ≥ 60%–99% asymptomatic carotid stenosis who are at increased risk of stroke, and CEA is preferred over CAS in patients > 70 years old with symptomatic ≥ 50%–99% carotid stenosis [203]. In lower extremity arterial disease, a network meta‐analysis of 82 RCTs (*n* = 12,796) concluded that endovascular device selection should be tailored to the clinical–anatomical context, with drug‐eluting technologies demonstrating superiority for infrapopliteal chronic limb ischemia [204]. Moreover, the SIRONA trial revealed that compared with paclitaxel‐coated balloons, sirolimus‐coated balloons resulted in noninferior clinical outcomes for femoropopliteal disease at 12 months (90.5% vs. 92.7%, *p*_noninferiority = 0.003) [205]. These findings suggest that device selection for lower extremity intervention requires careful anatomic and lesion‐specific consideration, and that the long‐term benefits of drug‐eluting technologies remain uncertain.

##### 3.1.3.2 Surgical Treatments

###### 3.1.3.2.1 Coronary Artery Bypass Grafting (CABG)

Commonly known as “bypass surgery,” this procedure restores blood flow by grafting new vessels from a location proximal to the stenosed or occluded coronary arteries to a location distal to the stenosis, thereby bypassing the blocked segment and achieving complete myocardial revascularization. In accordance with the guidelines, CABG is recommended as the preferred revascularization strategy in patients with left main CAD (especially when surgical risk is low and the anatomy is complex), in patients with multivessel disease and a SYNTAX score > 22 or with diabetes, and in those for whom complete revascularization cannot be achieved equivalently by PCI [206, 207].

###### 3.1.3.2.2 Carotid Endarterectomy

This surgical procedure removes atherosclerotic plaques from the inner wall of the carotid artery to restore blood flow and prevent subsequent ischemic stroke. Current guidelines continue to recommend CEA as the standard revascularization strategy for symptomatic carotid stenosis ≥ 50%–99%. For asymptomatic stenosis (≥ 60%–99%), CEA is recommended in select patients with a perioperative stroke/death risk < 3% [203, 208].

### 3.2 Novel Therapeutic Interventions

#### 3.2.1 Gene Therapy

##### 3.2.1.1 ASOs

ASOs are considered among the most advanced RNA‐based therapeutic methods. An ASO is composed of single‐stranded DNA, usually containing 13–20 nucleotides, and its effect is to bind to target mRNA to form a double‐stranded complex that is cleaved by RNase H1, thereby inhibiting protein synthesis and downregulating the expression of specific molecular targets [209, 210].

###### 3.2.1.1.1 ASOs Targeting ANGPTL3

Vupanorsen, a hepatically targeted antisense oligonucleotide that inhibits ANGPTL3 synthesis, was evaluated in a Phase 2b randomized, double‐blind, placebo‐controlled trial involving 286 adults with a non‐HDL‐C concentration ≥ 100 mg/dL and a TG concentration of 150–500 mg/dL on statin therapy. The trial demonstrated significant dose‐dependent reductions in non‐HDL‐C (up to 27.7%) and TG (up to 56.8%) levels at 24 weeks, although the effects on LDL‐C levels were more modest (up to 16.0%). However, the development program for this drug was discontinued because of safety concerns, specifically the dose‐dependent increases in the hepatic fat fraction and liver enzyme elevation, in addition to a higher incidence of injection‐site reactions [211].

###### 3.2.1.1.2 ASOs Targeting Lipoprotein (a) [Lp(a)]

Pelacarsen is a second‐generation ASO. It targets apo(a) production via chemical modifications that increase stability, reduce toxicity, and enable specific liver uptake. Clinical trials suggested that pelacarsen reduced Lp(a) levels in a dose‐dependent manner by up to 80% in patients with CVD, with a specific study showing Lp(a)‐C reductions of 29%–67% and a neutral to mild effect (2%–19% reduction) on corrected LDL‐C (LDL‐Ccorr) [212].

##### 3.2.1.2 siRNAs

siRNA can be used for potent and specific gene silencing mediated by double‐stranded RNA, which has successfully transitioned from basic research to clinical research [213, 214].

###### 3.2.1.2.1 siRNAs Targeting ANGPTL3

Two siRNAs targeting ANGPTL3 have been reported. Solbinsiran was evaluated in a Phase 2, double‐blind, randomized, placebo‐controlled trial involving 205 adults with mixed dyslipidemia on statin therapy, which demonstrated that compared with placebo, the 400 mg dose (administered subcutaneously on Days 0 and 90) significantly reduced apoB by 14.3% and LDL‐C by 16.8% from baseline on Day 180, with generally well‐tolerated safety profiles [215]. Similarly, zodasiran, another siRNA of ANGPTL3, has been investigated in two Phase 2 trials: the double‐blind, placebo‐controlled ARCHES‐2 trial (NCT04832971) in 204 adults with mixed hyperlipidemia showed significant dose‐dependent reductions in TG (up to 63% with 200 mg) and LDL‐C (up to 20%) levels [216], and the open‐label GATEWAY trial (NCT05217667) in 18 patients with HoFH demonstrated that quarterly zodasiran (200 mg or 300 mg) reduced fasting LDL‐C levels by approximately 36%–40% at Month 6, with greater reductions (up to 56%) in those receiving concomitant PCSK9 inhibitor therapy and a favorable safety profile with sustained efficacy during open‐label extension [217].

###### 3.2.1.2.2 siRNAs Targeting Lp(a)

Olpasiran (AMG 890), zerlasiran (SLN360), and lepodisiran (LY3819469) are all investigational GalNAc‐conjugated siRNAs that share the core mechanism of selectively degrading hepatocyte LPA mRNA to inhibit the synthesis of apolipoprotein(a), thereby potently and sustainably lowering circulating Lp(a) levels. Olpasiran, administered subcutaneously every 12 or 24 weeks in 281 participants with established ASCVD, achieved up to a > 95% median reduction in Lp(a) levels in the Phase 2 OCEAN(a)‐DOSE trial [218]. In a Phase 2 trial, zerlasiran demonstrated a well‐tolerated profile with primarily mild injection‐site reactions and achieved a placebo‐adjusted, time‐averaged reduction in Lp(a) of greater than 80% over 36 weeks across all dosing regimens [219]. In a Phase 1 trial, lepodisiran demonstrated a well‐tolerated profile with primarily mild injection‐site reactions and achieved dose‐dependent, long‐duration reductions in Lp(a) concentrations, with maximal median reductions ranging from 41% at the 4 mg dose to 97% at the 608 mg dose and a sustained 94% reduction on Day 337 following the 608 mg single dose [220]. Clinical studies focusing on the effect of Lp(a) reduction on plaque progression and in individuals with ASCVD are currently underway, and we look forward to the results being published (see the “Ongoing clinical trials” section).

#### 3.2.2 Immunotherapy

Inflammatory cytokines such as tumor necrosis factor alpha (TNF‐α), IL‐1β, and IL‐6 can induce apoptosis, pyroptosis, and necrosis within plaques, thereby affecting plaque stability and promoting plaque rupture or ulceration. Because AS is driven not only by lipid accumulation but also by chronic vascular inflammation, immunomodulatory strategies have attracted increasing attention as therapeutic approaches distinct from conventional lipid‐lowering therapy.

##### 3.2.2.1 Targeting Inflammatory Factors

mAbs targeting IL‐1β and IL‐6 can suppress inflammatory responses and reduce residual inflammatory risk in selected cardiovascular populations. Ziltivekimab, a humanized mAb targeting the ligand IL‐6, reduced the levels of high‐sensitivity C‐reactive protein (hsCRP) and other inflammatory and thrombotic biomarkers in patients with chronic kidney disease (CKD) and high atherosclerotic risk in the RESCUE Phase 2 trial [221]. Canakinumab, an anti‐IL‐1β mAb, reduced recurrent cardiovascular events in patients after MI with elevated hsCRP levels in the CANTOS trial without altering lipid levels [10]; however, the trial also demonstrated an increased risk of fatal infections. More recently, low‐dose IL‐2 therapy increased regulatory T‐cell levels and reduced arterial inflammation in ACS patients with residual systemic inflammation in the IVORY Phase 2 trial [222]. These findings support inflammation as a tractable therapeutic target in atherosclerotic CVD, although patient selection, infection risk, cost, and long‐term safety remain important considerations.

##### 3.2.2.2 Potential Cardiovascular Risks of Immune Checkpoint Modulation

Immune checkpoint modulation may have important vascular consequences in patients with AS. Although immune checkpoint inhibitors (ICIs) targeting PD‐1/PD‐L1 or CTLA‐4 are widely used in cancer treatment, clinical and mechanistic evidence indicates that they may disturb vascular immune homeostasis and accelerate atherosclerotic disease. In a matched cohort with an imaging substudy, ICI exposure was associated with a higher incidence of AS‐related cardiovascular events and a faster increase in plaque volume [223]. A recent retrospective cohort further reported ICI‐associated ASCVD events, including MI, ischemic stroke, coronary revascularization, and acute limb ischemia [224]. These clinical observations are supported by pathological and single‐cell studies showing that checkpoint pathways are active within human coronary, carotid, and aortic plaques. ICI exposure has been linked to altered plaque inflammatory composition, while recent immune‐checkpoint mapping revealed PD‐1, CTLA4, LAG3, and related checkpoint interactions across plaque immune‐cell networks [225, 226]. Recent studies further suggest that plaque‐associated PD‐1+ T‐cell subsets may be key cellular targets of checkpoint modulation, although their effects may depend on the antibody format, immune‐cell state, and plaque context [227, 228]. Several registered studies are now evaluating ICI‐associated plaque progression or adverse cardiovascular events, including NCT06519292 and NCT06785974 [229, 230]. Thus, checkpoint modulation should not be presented as a uniformly beneficial immune strategy in ASCVD; rather, it highlights the need for cardiovascular risk stratification and monitoring when immune‐activating therapies are used in patients with preexisting atherosclerotic risk.

##### 3.2.2.3 Chemokine‐Targeted Immunotherapy

Chemokine pathways are attractive targets for AS because they regulate leukocyte recruitment, macrophage localization, endothelial activation, T‐cell polarization, and plaque inflammation. Key axes include CCL2–CCR2, CXCL12–CXCR4, and CCL5‐related chemokine heterodimers. Clinical translation, however, remains limited. In a randomized, double‐blind, placebo‐controlled Phase 2 study, the CCR2‐blocking mAb MLN1202 reduced circulating hsCRP levels in individuals with atherosclerotic risk factors and elevated baseline CRP levels, supporting the proof‐of‐mechanism for the role of CCR2 blockade in residual inflammatory risk [231]. This trial did not assess plaque regression or cardiovascular outcomes. A subsequent Phase 2a FDG‐PET/CT study of MLN1202 in patients with stable ASCVD was registered but withdrawn without enrollment, whereas a Phase 1 study is evaluating CCR2‐targeted PET detection in human carotid AS [232, 233].

The major challenge is that chemokine signaling is highly context dependent. Broad blockade may be harmful when a chemokine pathway also maintains protective vascular immune niches. For example, mural cell‐derived CCL2 and MIF preserve a homeostatic macrophage niche in viable plaque regions, whereas disruption of this unit aggravates plaque necrosis and inflammation [234]. Similarly, vascular cell‐specific enhancement of CXCR4 signaling may be atheroprotective, while endothelial‐derived CXCL12 has been linked to AS progression [235, 236]. Recent work has also identified a noncanonical chemokine axis in which DC‐derived CCL17 signals through CCR8 on T cells to induce CCL3, thereby suppressing atheroprotective Foxp3^+^ Tregs and promoting AS [237]. More selective strategies, such as disrupting pathogenic chemokine heterodimers or timing CCL2–CCR2 inhibition according to circadian leukocyte recruitment, may therefore be preferable to nonspecific systemic inhibition [238, 239]. Future chemokine‐targeted therapies will likely require cell‐specific, temporally controlled, or interaction‐selective designs.

#### 3.2.3 Gene‐Editing Therapy

##### 3.2.3.1 Targeting the Low‐Density Lipoprotein Receptor (LDLR) Gene

One open‐label, single‐center, nonrandomized trial evaluated the safety and efficacy of NGGT006, an adeno‐associated virus (AAV) vector carrying an optimized human LDLR gene, in patients with HoFH due to LDLR mutations. Three patients received a single injection of NGGT006 at increasing doses (7.5 × 10^1^ ^2^, 1.5 × 10^13^, or 3.0 × 10^13^ vg/kg) and were followed for 52 weeks. NGGT006 was generally well tolerated, with no vector‐related severe adverse events; however, transient liver enzyme elevations occurred in all three patients and resolved after sirolimus and methylprednisolone therapy. The patient who received the highest dose achieved a rapid and sustained reduction in LDL‐C levels (from 11.17 to 0.28 mmol/L), which corresponds to an approximately 97.5% decrease. However, the two lower doses did not lead to meaningful LDL‐C reduction, and the long‐term safety and efficacy require further confirmation [240].

##### 3.2.3.2 Targeting the ANGPTL3 Gene

A Phase 1 ascending‐dose trial evaluated CTX310, a lipid nanoparticle (LNP)‐formulated CRISPR‐Cas9 therapy designed to induce loss‐of‐function mutations in the hepatic ANGPTL3 gene, in 15 adults with uncontrolled hypercholesterolemia, hypertriglyceridemia, or mixed dyslipidemia despite maximally tolerated lipid‐lowering therapy. All 15 participants had at least 60 days of follow‐up. The investigational treatment was generally well tolerated, with no dose‐limiting toxicity, and serious adverse events were reported in two participants (13%): one participant had a spinal disk herniation, and the other died suddenly 179 days after receiving the 0.1‐mg/kg dose. Infusion‐related reactions were reported in three participants (20%), and one participant with elevated aminotransferase levels at baseline had a transient elevation in aminotransferase levels. Dose‐dependent reductions in ANGPTL3 levels were observed, with mean decreases of 79.7% and 73.2% at the two highest doses (0.7 and 0.8 mg/kg), indicating successful hepatic gene editing [241]. These early findings require confirmation in larger populations and with longer term clinical follow‐up.

##### 3.2.3.3 Targeting the PCSK9 Gene

VERVE‐101, a first‐in‐class in vivo base editing therapy targeting PCSK9, provided a proof‐of‐concept for durable LDL‐C reduction in the Phase 1b Heart‐1 trial. However, its development was discontinued because of safety concerns (transient liver enzyme increases and platelet decreases) linked to its first‐generation LNP delivery system [242]. In the subsequent Phase 1b Heart‐2 study of VERVE‐102, a total of 35 patients with heterozygous familial hypercholesterolemia (HeFH) or patients with premature CAD were included, with at least 28 days of follow‐up. The results showed that a dose of 1.0 mg/kg could reduce PCSK9 levels by 88% and LDL‐C levels by 62%. This reduction persisted for at least 1 year of follow‐up in 15 patients, and mild‐to‐moderate infusion‐related reactions and transient alanine aminotransferase elevations occurred; aspiration pneumonitis occurred in one participant with gastroesophageal reflux disease [243]. In addition, another drug, YOLT‐101, is an experimental in vivo gene therapy that leverages adenine base editing and GalNAc‐modified LNP delivery to silence PCSK9. Data from a trial in six adults with HeFH and persistently high LDL‐C levels showed that a single intravenous dose was generally well tolerated; the most common side effects were transient infusion reactions and self‐limited liver enzyme increases. In the highest dose group (0.6 mg/kg, *n* = 3), dose‐dependent and durable reductions in plasma PCSK9 and LDL‐C levels were observed at 24 weeks, reaching 74.4% and 52.3%, respectively [244]. These are encouraging outcomes for further clinical development.

#### 3.2.4 Cell Therapy

##### 3.2.4.1 Mesenchymal Stem Cells (MSCs)

MSCs are multipotent stromal cells with self‐renewal, multilineage differentiation, immunomodulatory, and tissue‐repair capacities. Experimental and translational studies suggest that MSCs may modulate inflammatory signaling, promote vascular repair, and reduce plaque formation, but clinical evidence for direct antiatherosclerotic efficacy remains limited [245]. Therefore, MSC‐based approaches should currently be viewed as investigational strategies rather than established therapies for atherosclerotic vascular disease.

##### 3.2.4.2 CAR‐Treg Cell Therapy

Regulatory T cells (Tregs) are key suppressors of vascular inflammation, and engineered CAR‐Treg therapy has recently emerged as a cell‐based immunomodulatory strategy for treating AS. Antioxidized LDL‐specific CAR‐Tregs reduced macrophage foam‐cell formation in vitro and attenuated plaque development in AS‐prone mice through cell contact‐mediated suppression and secretion of inhibitory cytokines, suggesting that antigen‐directed Treg therapy may provide local immune suppression within atherosclerotic lesions [246]. However, this strategy remains at an early preclinical stage, and no human efficacy data exist for CAR‐Treg therapy in atherosclerotic patients. Major translational challenges include maintaining Treg stability and function in the inflammatory plaque microenvironment, ensuring specific homing to lesion sites without systemic immunosuppression, scaling up GMP‐grade manufacturing from low‐abundance Tregs, and establishing long‐term safety and persistence [247, 248]. Future studies will need to address these barriers in well‐designed preclinical models prior to any potential clinical testing.

##### 3.2.4.3 Induced Pluripotent Stem Cells (iPSCs)

iPSCs are generated by reprogramming somatic cells and can be differentiated into multiple cardiovascular and vascular lineages, including endothelial cells, VSMCs, macrophage‐like cells, and cardiomyocytes. Compared with their more direct application in myocardial repair after infarction, their therapeutic role in AS remains less established. No clinical trial has yet demonstrated iPSC‐based cell therapy as a direct treatment for atherosclerotic vascular lesions. Current registered cardiovascular iPSC studies focus mainly on disease modeling, biobanking, or myocardial regeneration rather than plaque‐targeted therapy. For example, the CAUSE trial used patient‐specific iPSC‐derived endothelial cells to study extreme‐trait CAD and fibromuscular dysplasia, whereas an ongoing iPSC biobank study aims to establish ethnically diverse CVD models [249, 250]. In contrast, registered iPSC‐derived cardiomyocyte trials in ischemic cardiomyopathy and heart failure address myocardial regeneration rather than atherosclerotic plaque therapy [251, 252].

Nevertheless, iPSC‐derived vascular cells provide useful human platforms for studying AS‐related mechanisms. Haplotype‐edited iPSC‐derived VSMCs have shown that the 9p21.3 CAD risk locus promotes atherogenic‐like VSMC phenotypes, including altered adhesion, contraction, proliferation, and vascular stability [253]. Another iPSC‐derived VSMC model revealed that the 9p21 risk locus increases vascular calcification, a key feature of advanced atherosclerotic lesions [254]. More recently, iPSC‐derived endothelial cells exposed to hyperoxic stress developed an AS‐like transcriptional phenotype, supporting their use for modeling ED [255]. Advances in the generation of iPSC‐derived mural progenitor cells with smooth muscle and pericyte potential may also support vascular repair modeling and future regenerative strategies, although this remains indirect evidence for AS therapy [256]. Recent work further indicates that the levels of circular RNAs increase during vascular cell differentiation and that these RNAs may serve as biomarkers for vascular disease, expanding the utility of iPSC‐derived vascular models for mechanism discovery and biomarker development [257]. Overall, the near‐term value of iPSCs in AS lies in patient‐specific disease modeling, vascular organoid construction, mechanism validation, and drug screening. Direct therapeutic use will require further evidence on cell maturity, lineage purity, tumorigenicity, immune compatibility, delivery route, and long‐term behavior within inflammatory plaque microenvironments.

### 3.3 Ongoing Clinical Trials

With the rapid development of new therapeutic interventions, several pivotal clinical trials are advancing globally to validate the safety and efficacy of these innovative therapies in the treatment of CVDs (Table 2). Here are several representative ongoing clinical trials.

**TABLE 2:** Summary of key ongoing clinical trials for novel therapeutic interventions in atherosclerosis.

Study title	Study design	Intervention	Endpoints	Follow‐up	References
VICTORION‐PLAQUE (NCT05360446)	Phase 3b, multicenter, randomized, double‐blind, placebo‐controlled, parallel‐group	Experimental: Inclisiran sodium 300 mg, SC on Day 1, Month 3, then Q6M Control: Matched placebo, SC on Day 1, Month 3, then Q6M.	Primary: % change in total coronary atheroma volume from baseline to Month 24. Secondary: % change in low‐attenuation plaque volume; % change in LDL‐C; plaque progression/regression.	24 months	[258]
AZURE‐Outcomes (NCT07000357)	Phase 3, international, randomized, double‐blind, placebo‐controlled, parallel‐group	Experimental: AZD0780, PO, QD Control: Matched placebo, PO, QD.	Primary: Time to first MACE‐PLUS (CV death, MI, ischemic stroke, acute limb ischemia, major amputation, urgent arterial revascularization). Secondary: 3P MACE, MI, urgent coronary revascularization, CV death, MALE, all‐cause death.	54 months	[259]
ARO‐DIMERPA (NCT07223658)	Phase 1/2a, double‐blind, placebo‐controlled, SAD + MAD	Experimental: ARO‐DIMERPA, SC, single or multiple ascending doses Control: normal saline (0.9%), SC.	Primary: TEAEs up to Week 36. Secondary: % change in LDL‐C, TG, apoC‐III, PCSK9 (baseline to Week 36); PK parameters.	36 weeks	[260]
EPI‐001 (ChiCTR2400081208)	Single‐center, open‐label, single‐arm, dose escalation and expansion	A single dose of EPI‐001 injection, IV. Dose escalation (low, middle, high dose) followed by a dose‐extension cohort at the optimal biological dose; total 32 participants (6/6/12/8).	Primary: AE/SAE, injection‐site reactions, lab abnormalities, CRP, vitals, ECG. Secondary: Change in PCSK9, lipid profile, PK, immunogenicity.	Not reported	[261]
EPI‐001T (ChiCTR2500114949)	Open‐label, single‐arm, dose escalation and expansion	Experimental: a single dose of EPI‐001T injection, IV. Dose escalation: 0.3–1.0 mg/kg (*n* = 6 each)	Primary: AE/SAE, vitals, ECG, lab abnormalities, CRP. Secondary: % change LDL‐C, PCSK9, other lipids; PK; anti‐PEG antibodies.	50 weeks	[262]
STX‐1150‐01 (NCT07428473)	Phase 1, open‐label, single‐ascending dose, and single‐ or multidose expansion	STX‐1150, IV infusion, CRISPR‐based epigenetic silencer targeting hepatic PCSK9.	Primary: TEAEs, SAEs, AESIs up to week 52. Secondary: Incidence of dose‐limiting toxicities (within 14 days), PCSK9, and LDL‐C changes, treatment‐induced immunogenicity.	52 weeks	[263]
OCEAN(a)‐Outcomes (NCT05581303)	Phase 3, double‐blind, randomized, placebo‐controlled, event‐driven	Experimental: Olpasiran, SC, Q12W Control: Placebo, SC, Q12W.	Primary: Time to first CHD death, MI, or urgent coronary revascularization. Secondary: CV death/MI/stroke, composite endpoints, % change Lp(a) at Week 48, and so forth	5 years	[264]
Lp(a) HORIZON (NCT04023552)	Phase 3, double‐blind, randomized, placebo‐controlled, event‐driven	Experimental: TQJ230 80 mg, SC, monthly Control: Placebo, SC.	Primary: Time to first CHD death, MI, or urgent coronary revascularization. Secondary: Same as OCEAN(a).	Minimum 2.5 years	[265]
ACCLAIM‐Lp(a) (NCT06292013)	Phase 3, double‐blind, randomized, placebo‐controlled, event‐driven	Experimental: Lepodisiran sodium, SC Control: Placebo, SC.	Primary: Time to first occurrence of any component of the cardiovascular death, nonfatal myocardial infarction, nonfatal ischemic stroke, and urgent coronary revascularization. Secondary: Cardiovascular death; fatal or nonfatal myocardial infarction; all‐cause death; change in Lp(a) at Week 4.	4.5 years	[266]
CTX320 FIH (ACTRN12623001095651)	Phase 1, open‐label, first‐in‐human, ascending single‐dose	Experimental: a single dose of CTX320 at varying dose levels, IV. No control group.	Primary: Incidence of dose‐limiting toxicities. Secondary: Percentage change in apoB; percentage change in Lp(a); frequency and severity of AEs, TEAEs; pharmacokinetics of CTX320.	12 months	[267]
CLEAR HIV Trial (NCT05488431)	Phase 2, randomized, quadruple‐masked, placebo‐controlled	Experimental: 180 mg Bempedoic acid, orally, QD, 52 weeks Control: Placebo, orally, QD, 52 weeks.	Primary: Change in arterial inflammation by FDG‐PET/CT from baseline to Week 52. Secondary: Changes in cardiometabolic, inflammatory, and immune‐activation markers	52 weeks	[268]
ZEPHYR (NCT07276282)	Phase 3, randomized, quadruple‐masked, placebo‐controlled	Experimental: Ziltivekimab, SC, once monthly Control: Placebo, SC, once monthly.	Primary: Change in percent atheroma volume from baseline to week 52. Secondary: Changes in plaque composition and inflammatory imaging measures.	15 months	[271]
ELLIPSE (NCT05975554)	Randomized, open‐label, parallel‐assignment	Low‐dose IL‐2, systemic administration/Control: Standard care.	Primary: Difference in gene expression in plaque Tregs between the IL‐2 and control groups at the time of surgery, assessed by scRNA‐seq. Secondary: Differences in Teff‐cell gene expression; Treg and Teff expression patterns from baseline to surgery; immune‐cell gene signatures in plaque and blood.	At time of surgery	[274]
IVORY‐FINALE (NCT06427694)	Observational follow‐up study	Follow‐up of participants from IVORY (NCT04241601) after low‐dose IL‐2 or placebo.	Primary: Number of participants with cardiovascular death, nonfatal myocardial infarction, resuscitated cardiac arrest, ischemic stroke, or unplanned coronary revascularization up to 5 years. Secondary: Cardiovascular death; unplanned coronary revascularization; resuscitated cardiac arrest; nonfatal myocardial infarction; hospitalization for heart failure; revascularization or amputation for peripheral vascular disease; hemorrhagic stroke; new atrial fibrillation; ventricular arrhythmia; ischemic stroke; cardiovascular hospitalization; all‐cause death.	5 years	[275]
Colchicine Use in Intracranial Atherosclerotic Disease (NCT05503225)	Phase 2, randomized, open‐label	Colchicine 0.5 mg orally once daily/Control: standard medical therapy.	Primary: Regression of intracranial stenosis, regression of plaque volume, and major adverse cardio‐ or cerebrovascular events, all at 12 months. Secondary: Longitudinal changes in plaque enhancement, plaque volume, white matter hyperintensity volume, number of silent lacunes, intracranial stenosis, and cognitive ability.	12 months	[276]
REC‐SAFECAD (NCT06572267)	Phase 2, randomized, single‐blind, placebo‐controlled pilot	Study biologic, IV infusion, monthly/Control: Saline.	Primary: Proportion of nontarget lesions with high perivascular fat attenuation on CCTA at 6 months. Secondary: Change in PVAT attenuation and nontarget lesion plaque composition; cardiac death; all‐cause death; target vessel myocardial infarction; clinically driven target lesion revascularization; any stroke, myocardial infarction, or revascularization; device‐oriented and patient‐oriented composite endpoints; changes in inflammatory biomarkers and PBMC gene expression; adverse events.	6 months	[277]
HeXell‐2020 (NCT07137104)	Phase 1/2a, open‐label, single‐arm, sequential dose escalation	Experimental: Allogeneic umbilical cord MSCs (HeXell‐2020), IV. Phase 1: 90 million cells/dose, either three doses or six doses at 2‐week intervals; Phase 2a: recommended Phase 2 dose.	Primary: Safety and tolerability; determination of the recommended phase 2 dose in Phase I; change in myocardial perfusion defect severity by SPECT. Secondary: coronary stenosis/plaque and calcium score by CCTA, cardiac function, angina, quality of life, and cardiovascular events.	12 months	[278]
Heartseed HS‐005/EMERALD (NCT07347197; jRCT2033250454)	Phase 1/2, multicenter, open‐label	Endocardial delivery of allogeneic iPSC‐derived cardiomyocyte spheroids for heart failure with reduced ejection fraction.	Primary: Safety and tolerability within 26 weeks. Secondary: Left ventricular ejection fraction and volumes by cardiac MRI and echocardiography, myocardial wall motion, myocardial blood flow and viability by SPECT, 6‐min walk distance, NT‐proBNP, and quality of life at 26 and 52 weeks.	**52 weeks（primary endpoint at 26 weeks）**	[252]

Abbreviations: 3P AE, adverse event; AESIs, Adverse Events of Special Interest; apoC‐III, apolipoprotein C‐III; CCTA, coronary computed tomography angiography; CHD, coronary heart disease; ChiCTR, Chinese Clinical Trial Registry; CRISPR, clustered regularly interspaced short palindromic repeats; CRP, C‐reactive protein; CT, computed tomography; CV, cardiovascular; ECG, electrocardiogram; FDG, 18F‐fluorodeoxyglucose; FIH, first‐in‐human; HIV, human immunodeficiency virus; IL‐2, interleukin‐2; iPSC, induced pluripotent stem cell; IV, intravenous; jRCT, Japan Registry of Clinical Trials; LDL‐C, low‐density lipoprotein cholesterol; Lp(a), lipoprotein(a); MACE, major adverse cardiovascular event; MACE, three‐point major adverse cardiovascular events; MACE‐PLUS, expanded composite of major adverse cardiovascular events; MAD, multiple ascending dose; MALE, major adverse limb event; MI, myocardial infarction; MRI, magnetic resonance imaging; MSC, mesenchymal stem cell; NCT, ClinicalTrials.gov identifier; NT‐proBNP, N‐terminal pro‐B‐type natriuretic peptide; PBMC, peripheral blood mononuclear cell; PCSK9, proprotein convertase subtilisin/kexin type 9; PEG, polyethylene glycol; PET, positron emission tomography; PK, pharmacokinetic(s); PO, per os (by mouth); PVAT, perivascular adipose tissue; Q12W, once every 12 weeks; Q6M, once every 6 months; QD, once daily; SAD, single‐ascending dose; SAE, serious adverse event; SC, subcutaneous; scRNA‐seq, single‐cell RNA sequencing; SPECT, single‐photon emission computed tomography; TEAE, treatment‐emergent adverse event; Teff, effector T cell; TG, triglycerides; Treg, regulatory T cell. Trial registry identifiers: ACTRN, Australian New Zealand Clinical Trials Registry.

#### 3.3.1 Lipid‐Related Targets

##### 3.3.1.1 PCSK9

###### 3.3.1.1.1 VICTORION‐PLAQUE (NCT05360446)

This is a Phase 3b, multicenter, randomized, double‐blind, placebo‐controlled, parallel‐group trial to evaluate the efficacy of inclisiran administered subcutaneously on Days 1, Month 3, and every 6 months thereafter in addition to maximally tolerated statin therapy versus placebo in reducing the primary endpoint of percent change in total coronary atheroma volume assessed by CCTA from baseline to Month 24 in approximately 600 participants with a diagnosis of nonobstructive CAD without previous cardiovascular events [258].

###### 3.3.1.1.2 AZURE ‐ Outcomes (NCT07000357)

This is a Phase 3, randomized, double‐blind, placebo‐controlled, parallel‐group, event‐driven trial to evaluate the effect of AZD0780 (an oral small‐molecule PCSK9 inhibitor) administered once daily versus placebo in reducing the risk of the primary MACEs composite endpoint in approximately 15,100 participants with established ASCVD or at high risk for a first ASCVD event, followed until the primary analysis censoring date (estimated primary completion on October 2029) [259].

###### 3.3.1.1.3 ARO‐DIMERPA (NCT07223658)

This is a Phase 1/2a, randomized, placebo‐controlled, dose‐escalating trial to evaluate the safety, tolerability, pharmacokinetics, and pharmacodynamics of ARO‐DIMERPA, a first‐in‐class dual‐function RNA interference (RNAi) therapeutic designed to silence both PCSK9 and APOC3 genes with a single molecule, administered subcutaneously in up to 78 adult subjects with mixed hyperlipidemia, with primary endpoints including the incidence of adverse events, changes in laboratory parameters, and dose‐limiting toxicities [260].

###### 3.3.1.1.4 EPI‐001 (ChiCTR2400081208)

This is an investigator‐initiated, single‐center, open‐label, single‐arm, dose‐escalation, and dose‐expansion trial to evaluate the tolerability, safety, pharmacokinetics, and preliminary efficacy of injection with EPI‐001, a novel epigenetic silencing therapy utilizing the EPIREG technology platform to precisely methylate and silence the PCSK9 gene without cutting DNA, administered by intravenous infusion in adult patients with refractory HeFH who have inadequately controlled LDL‐C levels (≥ 3.8 mmol/L in the dose‐escalation phase) despite stable lipid‐lowering therapy. The primary endpoints include the incidence of adverse events, dose‐limiting toxicities, and changes in laboratory parameters, and the secondary endpoints include the percent change from baseline in LDL‐C levels, PCSK9 protein levels, and duration of effect [261].

###### 3.3.1.1.5 EPI‐001T (ChiCTR2500114949)

This is an open‐label, single‐arm, dose‐escalation, and dose‐expansion investigator‐initiated trial (IIT) conducted at Renji Hospital, which is affiliated with Shanghai Jiao Tong University School of Medicine, to evaluate the tolerability, safety, pharmacokinetics, and preliminary efficacy of injection with EPI‐001T, a second‐generation epigenetic silencing therapy targeting PCSK9 via the EPIREG platform, administered subcutaneously in approximately 16 patients with established ASCVD or individuals at equivalent ASCVD risk, with a primary objective to assess safety and tolerability and confirm the recommended dose for subsequent studies, with six patients in the dose‐escalation phase and 10 in the dose‐expansion phase [262].

###### 3.3.1.1.6 STX‐1150‐01 (NCT07428473)

This is a Phase 1, open‐label, single‐ascending dose (Part 1) and single‐ or multidose expansion (Part 2) study to evaluate the safety, tolerability, pharmacokinetics, and pharmacodynamics of STX‐1150, a first‐in‐class CRISPR‐based epigenetic silencer designed to durably reduce LDL‐C by silencing the PCSK9 gene in the liver without altering the underlying DNA sequence (Figure 4), administered via single intravenous infusion in up to 64 adult participants with elevated LDL‐C levels and increased cardiovascular risk, with primary endpoints including the incidence of adverse events, dose‐limiting toxicities, and changes in laboratory parameters [263].

**FIGURE 4:**
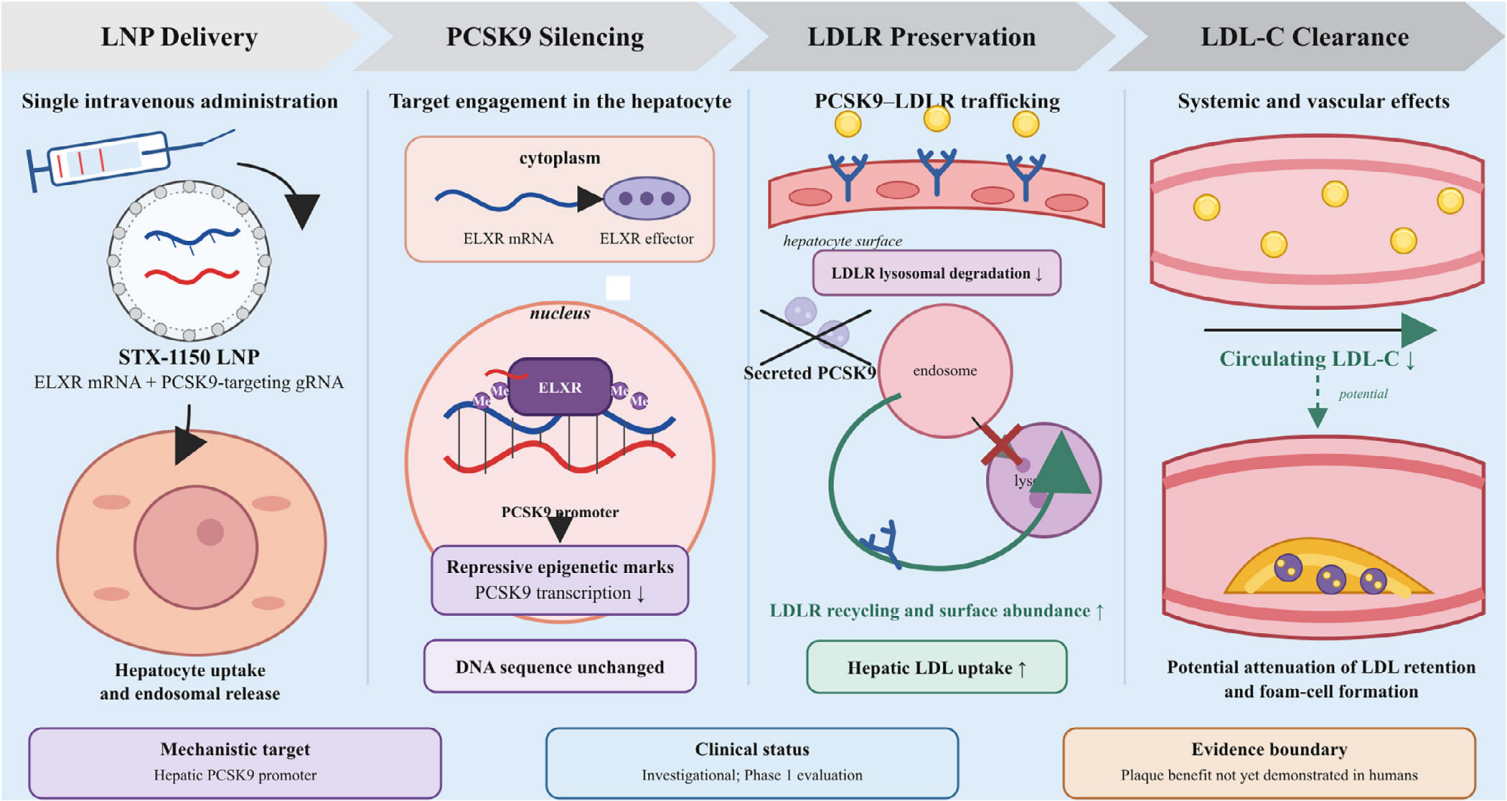
Proposed mechanism of action of STX‐1150 through epigenetic silencing of hepatic PCSK9. STX‐1150 comprises a lipid nanoparticle (LNP)‐encapsulated mRNA encoding the ELXR effector and a PCSK9‐targeting guide RNA (gRNA). Following hepatocyte uptake and endosomal release, the ELXR effector is expressed and directed by the gRNA to the PCSK9 promoter, where it establishes repressive epigenetic marks and reduces PCSK9 transcription without permanently altering the underlying DNA sequence. The resulting decrease in secreted PCSK9 is expected to limit PCSK9‐mediated lysosomal degradation of the LDLR, thereby increasing LDLR recycling and cell‐surface abundance, enhancing hepatic LDL uptake and reducing circulating low‐density lipoprotein cholesterol (LDL‐C). Sustained LDL‐C lowering may attenuate arterial LDL retention and foam‐cell formation; however, these effects on atherosclerotic plaques have not yet been demonstrated in humans. Solid arrows indicate direct mechanistic relationships, whereas the dashed arrow denotes a potential downstream vascular effect. STX‐1150 remains an investigational therapy undergoing Phase 1 clinical evaluation.

##### 3.3.1.2 Lp(a)

###### 3.3.1.2.1 OCEAN(a)‐Outcomes (NCT05581303)

This is a Phase 3, double‐blind, randomized, placebo‐controlled, multicenter, event‐driven trial to evaluate the effect of olpasiran administered subcutaneously every 12 weeks versus placebo in reducing the risk of the primary MACEs endpoint in 7297 participants with established ASCVD and elevated Lp(a) levels (≥ 200 nmol/L or ≥ approximately 80 mg/dL), followed until the primary completion date (estimated as March 31, 2028) [264].

###### 3.3.1.2.2 Lp(a) HORIZON (NCT04023552)

This is a Phase 3, double‐blind, randomized, placebo‐controlled, multicenter, event‐driven trial to evaluate the effect of pelacarsen administered subcutaneously every 4 weeks versus placebo in reducing the risk of the primary MACEs endpoint in 8323 participants with established CVD and elevated Lp(a) levels (≥ 70 mg/dL), with a key subgroup analysis in those participants with Lp(a) levels ≥ 90 mg/dL [265].

###### 3.3.1.2.3 ACCLAIM‐Lp(a) (NCT06292013)

This is a Phase 3, double‐blind, randomized, placebo‐controlled, multicenter, event‐driven trial to evaluate the effect of lepodisiran administered subcutaneously versus placebo in reducing the risk of the primary MACE in approximately 17,300 adults with elevated Lp(a) levels (≥ 175 nmol/L) and who have either established ASCVD with a prior event or revascularization or who were aged ≥ 55 years with high‐risk primary prevention criteria, who were followed for approximately 4.5 years [266].

###### 3.3.1.2.4 CTX320 FIH (ACTRN12623001095651)

This is a Phase 1, open‐label, multicenter, first‐in‐human, ascending single‐dose trial to evaluate the safety and tolerability of CTX320, a LNP formulation of CRISPR‐Cas9 targeting the LPA gene, administered as a single intravenous infusion in participants with elevated lipoprotein(a) levels and a history of atherosclerotic CVD or calcific aortic valve stenosis, with preliminary efficacy data demonstrating reductions in Lp(a) levels of up to 73% in the dose escalation phase [267].

##### 3.3.1.3 Bempedoic Acid

###### 3.3.1.3.1 CLEAR HIV Trial (NCT05488431)

This is an ongoing phase 2, randomized, quadruple‐masked, placebo‐controlled study examining whether bempedoic acid can reduce arterial inflammation by FDG‐PET/CT, and improve coronary plaque composition (by using CCTA) in HIV‐positive individuals with elevated cardiovascular risk. The trial remains active and is expected to conclude in March 2028 [268].

#### 3.3.2 Immunotherapy‐Related Clinical Trials

##### 3.3.2.1 Monoclonal Antibodies Against IL‐6

###### 3.3.2.1.1 ZEUS Trial (NCT05021835)

This is a Phase 3, randomized, placebo‐controlled cardiovascular outcomes trial evaluating whether IL‐6 ligand inhibition reduces cardiovascular events in patients with ASCVD, CKD and systemic inflammation (hsCRP ≥ 2 mg/L). Although once‐monthly ziltivekimab 15 mg achieved target engagement, producing the expected reductions in free IL‐6 and hsCRP, it did not reduce the primary MACEs endpoint compared with placebo (HR: 0.99; 95% CI, 0.88–1.11); serious infections were more frequent with ziltivekimab, whereas all‐cause mortality did not differ. Two further outcome trials with ziltivekimab, HERMES and ARTEMIS, are continuing and are anticipated to report in the first half of 2027 [269, 270].

###### 3.3.2.1.2 NCT07276282

This is an ongoing Phase 3, randomized, quadruple‐masked, placebo‐controlled trial evaluating once‐monthly ziltivekimab in approximately 332 patients with acute MI. The primary endpoint is the change in PAV from baseline to 52 weeks [271].

##### 3.3.2.2 Drugs Against IL‐1

###### 3.3.2.2.1 NCT06691217 (TET2‐CHIP)

This is a Phase 2, randomized, quadruple‐masked, placebo‐controlled trial evaluating canakinumab, an anti‐IL‐1β mAb, in patients with established coronary heart disease who carry TET2‐mutant clonal hematopoiesis or no myeloid driver mutation. The primary endpoint is the change in perivascular fat attenuation index on CCTA from baseline to 48 weeks, compared between the canakinumab and placebo arms. The trial is recruiting 120 participants, with an estimated primary completion in January 2029 [272].

###### 3.3.2.2.2 NCT05177822 (VCU‐ART4)

This is a Phase 2, randomized, quadruple‐masked, placebo‐controlled trial evaluating interleukin‐1 blockade with anakinra, a recombinant interleukin‐1 receptor antagonist, in patients with ST‐segment elevation MI undergoing reperfusion, with the aim of preventing heart failure. The primary endpoint is peak oxygen consumption at 6 weeks. The trial has completed enrolment of 84 participants and is expected to complete in December 2027 [273].

##### 3.3.2.3 Low‐Dose IL‐2

###### 3.3.2.3.1 NCT05975554

This is an interventional study evaluating low‐dose IL‐2 in approximately 30 patients with carotid atherosclerotic plaques to assess plaque Treg gene signatures and the immune landscape using single‐cell RNA sequencing [274].

###### 3.3.2.3.2 NCT06427694 (IVORY‐FINALE)

This is an observational follow‐up study of approximately 60 participants who completed the IVORY trial, evaluating MACEs after low‐dose IL‐2 or placebo [275].

##### 3.3.2.4 Other Anti‐Inflammatory Interventions

###### 3.3.2.4.1 NCT05503225

This is an ongoing Phase 2, randomized trial evaluating low‐dose colchicine (0.5 mg daily) in approximately 72 patients with symptomatic intracranial atherosclerotic disease, with plaque regression and major adverse cardio‐ or cerebrovascular events assessed over 12 months [276].

###### 3.3.2.4.2 NCT06572267

This is an ongoing Phase 2, randomized, single‐blind, placebo‐controlled pilot trial evaluating meplazumab in 18 patients with CAD and high perivascular fat attenuation, with changes in plaque composition, perivascular fat attenuation, and inflammatory biomarkers assessed over 6 months [277].

#### 3.3.3 Cell Therapy‐Related Clinical Trials

##### 3.3.3.1 Mesenchymal Stem Cell Therapy

###### 3.3.3.1.1 NCT07137104

This is an open‐label, single‐arm Phase 1/2a trial evaluating the safety, tolerability, and preliminary effectiveness of HeXell‐2020, an allogeneic umbilical cord‐derived MSC product, in patients with stable CAD. The study tracks coronary stenosis and calcium score using coronary CT angiography (CCTA), as well as angina symptoms and cardiac function [278].

##### 3.3.3.2 Induced Pluripotent Stem Cell Therapy

###### 3.3.3.2.1 NCT07347197 (EMERALD; HS‐005)

This is a recruiting, open‐label Phase I/II trial evaluating endocardial delivery of allogeneic iPSC‐derived cardiomyocyte spheroids in 14 patients with reduced‐ejection‐fraction heart failure caused by ischemic heart disease or dilated cardiomyopathy. The primary outcome is safety; secondary outcomes include cardiac function and quality of life [252].

## 4 Challenges and Future Directions

At present, the clinical treatment of ASCVD has achieved great success. Statins and PCSK9‐targeted therapies can rapidly lower LDL‐C to target levels, yet the residual risk of cardiovascular events remains substantial. This suggests that AS is not merely a lipid disorder [3]. With the advancement of technology and the discovery of new mechanisms in immunology, neurology, and microbiology, they mark the shift of AS toward multidimensional exploration and precise treatment. However, this shift still faces complex challenges in clinical implementation and translation.

### 4.1 Translational Barriers

In the field of AS drug research, there is a certain discrepancy between preclinical results and clinical efficacy in human clinical trials. Many therapeutic drugs that have performed well in animal models have not shown clinical benefits after they enter clinical trials, highlighting the limitations of current drug development [279]. For example, in clinical studies on inflammatory pathways, although the CANTOS trial validated the value of the IL‐1β pathway, other drugs did not achieve consistent results. Although colchicine has good therapeutic effects in patients with chronic stable coronary heart disease, it showed a negative result in the CLEAR trial for acute MI [280]. On the one hand, genetically modified mouse models remain the cornerstone of preclinical research, but they cannot fully reproduce the lethal phenotype of human diseases, as these mice rarely experience spontaneous plaque rupture or acute thrombotic events [279, 281]. On the other hand, atherosclerotic plaques in mice usually present a stable phenotype, with a thick fibrous cap, and lack the “vulnerable” characteristics of human thin fibrous cap AS, including large necrotic cores and intraplaque hemorrhage [279]. In addition, mice have natural resistance to coronary AS, and most of the lesions occur in the aorta and carotid arteries, making it difficult to simulate the unique hemodynamic environment of human coronary arteries. In recent years, researchers have attempted to induce plaque instability using surgical models or novel genetically engineered mouse strains to address the aforementioned shortcomings. However, these models often have severe complications, limiting their application in long‐term drug screening [281].

Another challenge in translation is that most preclinical studies in animal models of AS have examined only male animals, and even when both sexes are included, adequately powered comparisons with sex as an independent variable remain rare [282]. Sex is an important determinant of plaque phenotype and clinical course: overall plaque burden and markers of inflammation are greater in men than in women, and although younger women are relatively protected, by the seventh decade the incidence of MI in women surpasses that of men [282]. Consistent with these clinical observations, age‐stratified in vivo OCT of culprit lesions in ACS showed that plaque rupture and features of plaque vulnerability increased with age in women but not in men, whereas the overall distribution of plaque rupture versus erosion was similar between sexes [283].

### 4.2 Technology‐Driven Solutions

In recent years, new technologies, including nanotechnology, microphysiological systems, and artificial intelligence (AI), have been used to address these translational challenges. First, nanotechnology makes it possible to deliver drugs precisely to the lesion and stabilize plaques, while also reducing the systemic side effects typically associated with standard therapies. The newly developed confined cascade enzyme nanoreactors possess multiple enzyme‐like activities and can efficiently scavenge ROS within AS plaques through cascade catalysis, thereby alleviating local oxidative stress [284]. In addition, the researchers engineered platelet membrane‐modified extracellular vesicles derived from M2 macrophages (P‐M2EVs) using a membrane fusion technique. This system mimics the natural interaction between platelets and macrophages, demonstrating enhanced plaque‐targeting ability and tissue penetration in vivo, without obvious systemic toxicity [285]. Second, microphysiological systems, or “Atherosclerosis‐on‐a‐Chip,” can help bridge the gap between conventional in vitro experiments and in vivo models. This type of platform simulates the complex three‐dimensional structure and hemodynamic environment of human blood vessels, enabling research conditions that are closer to physiological reality [286]. Recent studies have described dynamic coculture systems comprising human endothelial cells, SMCs, and monocytes. These systems enable the study of atherogenic processes, including monocyte extravasation and foam‐cell formation, and provide a platform for evaluating biological agents whose effects may not be reproduced in rodent models because of species differences [287, 288].

Third, spatial transcriptomics and proteomics redefine our understanding of the structure of atherosclerotic plaques by preserving the spatial background of molecular expression in tissue in situ [289]. For example, new spatial omics studies have shown that macrophage subtypes are not fixed and unchanging but undergo gradual transdifferentiation within the boundary region between the fibrous cap and necrotic core, resulting in a transition from an inflammatory HMOX1^+^ phenotype to a lipid‐handling TREM2^+^ phenotype [12]. In addition, mass spectrometry imaging technology promotes the integration of multimodal spatial omics, which can simultaneously analyze lipidome, proteome, and metabolome at cellular resolution, thereby elucidating heterogeneous intercellular signal exchange, lesion vulnerability, and sex‐specific disease mechanisms within plaques [290]. In the future, integrating multiomics data with emerging technologies may help advance precision medicine for AS.

Fourth, AI has become integrated into AS research, extending its utility from image diagnosis to risk prediction, basic research, and beyond. In the field of imaging, deep learning algorithms have achieved automated plaque segmentation, calcification score measurement, and precise detection of high‐risk features in CCTA [291]. The latest Quantitative Cardiovascular Imaging research group consensus statement in 2026 further suggests that personalized drug therapy should be guided by AI‐analyzed total plaque volume percentile curves and that drug therapy should be initiated for the presence of any plaque, while high‐intensity intervention is needed for those with a total plaque volume at or above the 70th percentile [291]. For risk prediction, machine learning models integrate multiple types of omics data, improving the predictive performance of traditional models and enhancing risk prediction 15 years before disease occurrence [292]. In terms of basic research, AI integrates multiple types of omics data to reveal cellular heterogeneity and molecular characteristics within plaques and to identify novel diagnostic biomarkers and therapeutic targets [293]. In the future, the further integration of AI with natural language processing, wearable devices, and multimodal data will drive AS from group‐based diagnosis and treatment toward a precise management model that covers the entire life cycle and individualization.

### 4.3 Clinical Implementation

In real‐world clinical practice, we need to rethink how to diagnose, stratify, and treat AS. The first issue is that there is still a lack of biomarkers that can reflect specific pathological processes of AS. For example, the NICI has been proven to be an important regulatory link in vascular inflammation, but there are no imaging methods or blood biomarkers in clinical practice for evaluating neural activity or neuroimmune crosstalk around the blood vessels of patients [2]. The perivascular fat attenuation index derived from CCTA is a noninvasive alternative index [294]. Recent studies have shown that imaging‐defined carotid plaque characteristics are associated with ischemic stroke and that carotid perivascular adipose tissue attenuation is associated with stroke and transient ischemic attack in patients with symptomatic carotid artery disease [295]. As for trained immunity, it can currently be studied only through the measurement of monocyte cytokine secretion function or epigenetic profiling analysis in experimental settings, which is still far from clinical practice. To support mechanism‐based diagnosis and treatment, these biomarkers need to be developed and validated for clinical use—for example, through PET tracers that target specific immune cells, liquid biopsies that capture cell state‐specific epigenetic signals, or integrated multiomics panels [296].

The second issue is the combination of treatment strategies. At present, multiple new intervention methods, such as anti‐inflammatory mAbs, RNA drugs, and cellular immune regulation therapies, have entered the clinical development stage, but there is still a lack of clear guidance on how to use them in conjunction with existing therapies. To solve this problem, it is necessary to design a reasonable combination therapy plan to achieve simultaneous intervention in different pathogenic pathways. A Mendelian randomization analysis revealed that simultaneous inhibition of LDL‐C, Lp(a), and the IL‐6 pathway could reduce the risk of coronary heart disease by about 30%, which was significantly better than that associated with the single‐target intervention [297]. In addition, in the future, patients can be stratified on the basis of the molecular pathological characteristics of plaques, such as phenotypes dominated by SMC proliferation or phenotypes dominated by an inflammatory response, which can aid in the personalized selection of treatment strategies [298]. However, achieving the above goals requires clinical studies with prospective trial designs and long‐term follow‐up systems to evaluate the safety and effectiveness of the combination regimen.

The third issue involves regulatory science and health economics. Traditional clinical trials require cardiovascular outcomes as the primary endpoint, which typically requires large sample sizes and long‐term follow‐up, but may have a high threshold for the development of new drugs. If regulatory agencies can accept alternative endpoints strongly associated with clinical events in different populations on the basis of sufficient verification, it can accelerate the drug development process [299]. AI‐based plaque quantification and radiotranscriptomics are advancing plaque characterization. These tools facilitate the validation of alternative endpoints, streamline experimental design, and accelerate clinical research and development decisions [291, 300]. Cost also remains a barrier: biologics, gene therapies, and cell‐based products are expensive, and equitable access will depend on value‐based pricing, risk‐sharing agreements, and streamlined manufacturing. Clinical translation will depend on a deeper understanding of disease biology, validated biomarkers, efficient trial designs, workable regulatory pathways, and delivery models that ensure equitable access.

## 5 Conclusion

AS has shifted from being considered a lipid‐storage disorder to a systemic disease shaped by interactions across the vascular, immune, metabolic, and nervous systems. These insights are now being translated into distinct therapies—anti‐inflammatory mAbs, RNA‐based strategies targeting PCSK9, ANGPTL3, and Lp(a), and early‐phase cell and gene‐editing approaches. Looking ahead, the management of AS is moving toward integrated risk assessment. This shift demands that clinical risk stratification evolve beyond single indicators to a comprehensive integration of lipid, inflammatory, and multiomic markers. Therapeutically, future clinical success will rely not on sequential monotherapy, but on selecting tailored combination regimens for individual patients by integrating polygenic risk scores, single‐cell transcriptomic features, and plaque imaging phenotypes. Although challenges remain, continued advances in emerging technologies and mechanistic insights are steadily driving the shift toward personalized, precision therapy for AS.

## Author Contributions

**Enge Zhou and Chenxi Zhan**: responsible for the conceptualization, literature search, and drafting of the manuscript. **Yuting Li and Yi Zeng**: investigation and validation. **Renfei Luo**: visualization. **Shixing Liu and Xin Ouyang**: table compilation. **Renqiang Yang**: reviewing. **Nianlong Yan and Long Jiang**: supervision, writing – review and editing. All the authors have read and approved the final manuscript.

## Funding

This work was financially supported by the following: the National Natural Science Foundation of China (No. 82360181); the Natural Science Foundation of Jiangxi Province (No. 20242BAB25458); Noncommunicable Chronic Diseases‐National Science and Technology Major Project (No. 2023ZD0503900, 2023ZD0503902). The Hope for Rare Foundation and Homy FH China Patient Network (No. FH2025‐012).

## Ethics Statement

The authors have nothing to report.

## Conflicts of Interest

The authors declare no conflicts of interest.

## Supporting information




**Supporting Information File**: 1 mco271095‐sup‐0001‐TableS1.docx

## Data Availability

No additional data are available.
