# Supplementary material for: Atherosclerosis: From Pathogenesis to Therapeutic Interventions
Source: MedComm (2020). 2026 Oct 29;7(11):e71095. doi: 10.1002/mco2.71095 (PMC13717303; doi:10.1002/mco2.71095)
Supplement: Supplementary file 1 [file MCO2-7-e71095-s001.docx]

**SUPPORTING INFORMATION**

**Atherosclerosis: From Pathogenesis to Therapeutic Interventions**

**Enge Zhou^1#^, Chenxi Zhan^2#^, Yuting Li^1^, Yi Zeng^1^, Renfei Luo^1,3^, Xin Ouyang^1^, Shixing Liu^1^, Renqiang Yang^1^, Nianlong Yan ^1,2*^, Long Jiang^1*^**

SHORT TITLE: Atherosclerosis: Pathogenesis and Therapeutics

**AFFILIATIONS:**

^1^Department of Cardiovascular Medicine, The Second Affiliated Hospital, Jiangxi Medical College, Nanchang University, Nanchang, Jiangxi, China.

^2^Department of Biochemistry and Molecular Biology, School of Basic Medical Sciences, Jiangxi Medical College, Nanchang University, Nanchang, Jiangxi, China;

^3^Affiliated Rehabilitation Hospital of Nanchang University, Nanchang, Jiangxi, China;

Correspondence: Nianlong Yan (yannianlong@ncu.edu.cn); Long Jiang (skyiadx@hotmail.com)

**^#^** Enge Zhou and Chenxi Zhan contributed equally to this work.

*Correspondence

**Contents**

Table S1 Pharmacological interventions in clinical studies relevant to atherosclerosis.
References.

# Table S1 Pharmacological interventions in clinical studies relevant to atherosclerosis.

| **Drug(s)** | **Study country^[Ref.]^** | **Population / age** | **Follow-up** | **Treatment / comparator** | **LDL-C levels or change** | **Imaging / assessment** | **Main findings** |
| --- | --- | --- | --- | --- | --- | --- | --- |
| **Lipid-lowering drugs** | | | | | | | |
| **Statins** | | | | | | | |
| Atorvastatin | Zhang X et al. (2013) China^[1]^ | 100 patients with new-onset unstable angina and borderline coronary lesions; randomized Age: Intensive-dose atorvastatin: 64.5±13.8; moderate-dose atorvastatin: 65.5±6.2 years | 9 months | Atorvastatin intensive regimen: 20, 40, 60, and 80 mg/day in weeks 1–4, respectively, then 80 mg/day through month 9; comparator: atorvastatin 20 mg/day for 9 months. | Intensive-dose atorvastatin: 105.55±22.65 to 62.37±15.93 mg/dL; moderate-dose atorvastatin: 106.13±20.52 to 80.04±17.77 mg/dL (baseline to 9 months). | Coronary angiography and IVUS | Plaque volume did not progress with 80 mg/day; it increased with 20 mg/day (within-group P<0.05). |
| Pitavastatin, Atorvastatin | Hiro T et al. (2009) Japan^[2]^ | 307 ACS patients undergoing PCI randomized; 252 with serial IVUS Age: Pitavastatin: 62.5±11.5; atorvastatin: 62.4±10.6 years (252 patients with serial IVUS) | 8–12 months | Pitavastatin 4 mg/day vs. atorvastatin 20 mg/day | Pitavastatin: 130.9±33.3 to 81.1±23.4 mg/dL; atorvastatin: 133.8±31.4 to 84.1±27.4 mg/dL (serial-IVUS population). | IVUS | Plaque volume change: −16.9±13.9% vs. −18.1±14.2%; between-group P=0.50. Pitavastatin met the prespecified noninferiority criterion. |
| Atorvastatin, Rosuvastatin | Nicholls SJ et al. (2011) Multinational^[3]^ | Patients with coronary artery disease; 1,039 with serial IVUS Age: 18–75 years (registered eligibility) | 104 weeks | Atorvastatin 80 mg/day vs. rosuvastatin 40 mg/day | On-treatment LDL-C: atorvastatin 70.2 vs. rosuvastatin 62.6 mg/dL. | IVUS | PAV decreased by 0.99 vs. 1.22 percentage points (P=0.17). TAV decreased by 4.42 vs. 6.39 mm³ (P=0.01), respectively. |
| Atorvastatin | Okazaki S et al. (2004) Japan^[4]^ | 70 ACS patients randomized (35 per group); 48 with serial IVUS Age: Atorvastatin: 61.3±10.1; control: 62.5±11.2 years (mean±SD; randomized population, n=35 per group). | 6 months | Atorvastatin 20 mg/day vs. control | Atorvastatin: 41.7% reduction; control: 0.7% increase. | IVUS | Plaque volume change: −13.1±12.8% with atorvastatin vs. +8.7±14.9% with control (P<0.0001). |
| Pravastatin | Tani S et al. (2005) Japan^[5]^ | 75 patients with stable CAD completed serial IVUS: pravastatin n=52, control n=23. Age: Pravastatin: 63±10; control: 62±13 years (mean±SD; completers, n=52 and n=23). | 6 months | Pravastatin initially 10 or 20 mg/day according to baseline LDL-C, reduced by 5 mg if LDL-C fell below 70 mg/dL, vs. no pravastatin; both groups received dietary counseling. | Pravastatin: 130±38 to 104±20; control: 123±28 to 120±30 mg/dL (mean±SD; baseline to 6 months). | Three-dimensional IVUS | Pravastatin group plaque volume decreased by 14.4% (within-group P<0.0001); MDA-LDL fell by 12.7%. These are within-group changes. |
| Atorvastatin, Pravastatin | Nissen SE et al. (2004) United States^[6]^ | 654 CAD patients randomized; 502 with serial IVUS Age: Atorvastatin: 55.8±9.8; pravastatin: 56.6±9.2 years (serial-IVUS population) | 18 months | Atorvastatin 80 mg/day vs. pravastatin 40 mg/day | Atorvastatin: 150.2±27.9 to 78.9±30.2 mg/dL; pravastatin: 150.2±25.9 to 110.4±25.8 mg/dL. | IVUS | Atorvastatin halted progression, whereas pravastatin showed progression. Median relative atheroma-volume change: −0.4% vs. +2.7%. |
| Pitavastatin, Atorvastatin | Toi T et al. (2009) Japan^[7]^ | 160 ACS patients after emergency PCI; 80 per group Age: 62.0±10.6 years (overall) | 2–3 weeks | Pitavastatin 2 mg/day vs. atorvastatin 10 mg/day | Pitavastatin: 114.7±36.7 to 74.8±20.6 mg/dL; atorvastatin: 122.0±40.7 to 85.3±28.0 mg/dL. | VH-IVUS | Plaque-volume and fibrofatty-volume indices decreased with pitavastatin. No significant corresponding changes were found with atorvastatin; exploratory early imaging findings. |
| Rosuvastatin, Atorvastatin | Hong YJ et al. (2011) Korea^[8]^ | 128 patients with intermediate coronary stenosis; rosuvastatin n=65, atorvastatin n=63 Age: Rosuvastatin: 59±10; atorvastatin: 58±10 years | 11 months | Rosuvastatin 20 mg/day vs. atorvastatin 40 mg/day | Rosuvastatin: 122±37 to 62±20 mg/dL; atorvastatin: 117±38 to 70±24 mg/dL. | IVUS | TAV change: −4.4±7.3 vs. −3.6±6.8 mm³ (P=0.50); PAV change: −0.73±2.05 vs. −0.19±2.00 percentage points (P=0.14). |
| Atorvastatin | Lee SW et al. (2012) Hong Kong, China^[9]^ | 40 statin-naive stable-angina patients randomized, 20 per group; 39 completed imaging Age: 10 mg: 65.05±9.99; 40 mg: 63.70±9.80 years | 6 months | Atorvastatin 10 mg/day vs. 40 mg/day | 10 mg: 122.39±39.54 to 68.53±26.80 mg/dL; 40 mg: 112.35±27.14 to 52.12±12.63 mg/dL. | VH-IVUS | Percentage plaque-volume change: +0.38±4.05 vs. −1.50±3.85 percentage points (P=0.014); higher dose limited relative necrotic-core expansion. |
| Atorvastatin, Pitavastatin, Pravastatin, Fluvastatin | Matsushita K et al. (2016) Japan^[10]^ | 118 ACS patients randomized; 102 with serial IVUS Age: 62.8±10.2 years (serial-IVUS population) | Approximately 10 months | Atorvastatin 20, pitavastatin 4, pravastatin 10, or fluvastatin 30 mg/day | Moderate-intensity groups: 138 to 75 mg/dL; low-intensity groups: 146 to 105 mg/dL. | IVUS | Plaque-volume changes: −11.1%, −8.1%, +0.4%, +3.1%, respectively (ANOVA P=0.007). Moderate-intensity regimens produced greater regression. |
| Pitavastatin, Pravastatin | Nozue T et al. (2012) Japan^[11]^ | 164 patients randomized; 119 with serial IVUS Age: Pitavastatin: 66±9; pravastatin: 67±11 years (mean±SD; serial-IVUS population, n=58 and n=61). | 8 months | Pitavastatin 4 mg/day vs. pravastatin 20 mg/day | Pitavastatin: 126±28 to 74±22; pravastatin: 137±35 to 95±23 mg/dL (mean±SD; baseline to 8 months). | VH-IVUS | Fibrofatty volume decreased and calcium volume increased in both groups. Changes in these components did not differ significantly between groups. |
| Rosuvastatin, Atorvastatin | Thondapu V et al. (2019) China^[12]^ | 80 patients in the two randomized treatment arms; 43 completed serial imaging Age: Rosuvastatin: 57.5; atorvastatin: 54.2 years (means) | 6 and 12 months | Rosuvastatin 10 mg/day vs. atorvastatin 20 mg/day | Baseline / 6 / 12 months, mg/dL: rosuvastatin 100±21 / 72±29 / 76±34; atorvastatin 115±28 / 76±28 / 80±32. | OCT and IVUS | Minimum FCT increased in both groups. At 12 months: 171.5±67.8 vs. 127.0±66.8 µm. TAV decreased within the rosuvastatin group (P=0.047), not within atorvastatin (P=0.07). |
| Atorvastatin | Yokoyama M et al. (2005) Japan^[13]^ | 50 patients undergoing PCI randomized, 25 per group Age: Atorvastatin: 62.1±10.2; control: 64.4±8.7 years (42 patients with follow-up: 20 vs. 22) | 6 months | Atorvastatin 10 mg/day vs. control | Atorvastatin: 133±13 to 87±29 mg/dL (P<0.0001). | IVUS radiofrequency analysis | Atorvastatin increased integrated backscatter (−53.8±4.5 to −51.2±4.9 dB) and reduced plaque volume; no corresponding change in control. |
| Atorvastatin, Rosuvastatin | Lee CW et al. (2012) Korea^[14]^ | 350 statin-naive patients randomized; 271 with serial IVUS Age: Atorvastatin: 57.6±7.6; rosuvastatin: 55.3±9.4 years (serial-IVUS population, n=143 and n=128). | 6 months | Atorvastatin 20 mg/day vs. rosuvastatin 10 mg/day | Atorvastatin: 110±31 to 56±18; rosuvastatin: 109±31 to 53±18 mg/dL (baseline to 6 months). | IVUS | Relative TAV change: −3.9±11.9% vs. −7.4±10.6% (P=0.018). PAV change: −0.3±4.2 vs. −1.1±3.5 percentage points (P=0.157). |
| Rosuvastatin | Egede R et al. (2013) Denmark^[15]^ | 87 statin-naive STEMI patients randomized; 44 low-dose and 43 high-dose Age: Low dose: 60.0±10.3; high dose: 62.0±9.9 years | 12 months | Rosuvastatin 5 mg/day (titrated if lipid targets unmet) vs. 40 mg/day | Low dose: 3.1±0.7 to 2.0±0.4 mmol/L; high dose: 3.1±1.0 to 1.6±0.7 mmol/L. | VH-IVUS | Necrotic-core volume decreased 7.6% vs. 14.2%; between-group P=0.38. A significant within-group reduction occurred only with high dose. |
| Atorvastatin | Guo S et al. (2012) China^[16]^ | 228 patients with stable coronary plaques; randomized to five groups Age: Placebo / 10 / 20 / 40 / 80 mg: 62.07±8.51 / 62.64±12.00 / 59.18±8.48 / 58.91±12.90 / 58.95±9.68 years | 3–6 months | Atorvastatin 10, 20, 40, or 80 mg/day vs. placebo | 80 mg group: 2.83±0.66 to 1.81±0.32 mmol/L; LDL-C fell in all atorvastatin groups. | Coronary angiography and VH-IVUS | Plaque volume decreased with 40 and 80 mg/day. Necrotic fraction increased with placebo and 10 mg/day but not with 20–80 mg/day. |
| Simvastatin, Rosuvastatin | Hong MK et al. (2009) Korea^[17]^ | 100 patients with non-significant coronary lesions; 50 per randomized arm Age: Simvastatin: 58±10; rosuvastatin: 59±9 years (50 patients per group). | 12 months | Simvastatin 20 mg/day vs. rosuvastatin 10 mg/day | Simvastatin: 119±30 to 78±20 mg/dL; rosuvastatin: 116±28 to 64±21 mg/dL (baseline to 12 months). | VH-IVUS | Pooled necrotic-core volume decreased from 15.7 to 13.7 mm³, and fibrofatty volume increased from 4.3 to 5.5 mm³. Component-volume changes did not differ significantly between regimens. |
| Atorvastatin, Pravastatin | Kawasaki M et al. (2005) Japan^[18]^ | 52 hyperlipidemic patients; pravastatin n=17, atorvastatin n=18, diet n=17 Age: Atorvastatin: 66±8.7; pravastatin: 67±7.8; dietary control: 66±6.4 years. | 6 months | Pravastatin 20 mg/day vs. atorvastatin 20 mg/day vs. dietary control | Atorvastatin: 155±22 to 95±15; pravastatin: 149±19 to 102±13; dietary control: 152±20 to 149±24 mg/dL (baseline to 6 months). | Three-dimensional IB-IVUS | Lipid fraction decreased from 25.5% to 21.9% with pravastatin and 26.5% to 19.9% with atorvastatin. Fibrous fraction increased; degree of stenosis was not reduced. |
| Rosuvastatin | Kini AS et al. (2013) United States^[19]^ | 87 patients with multivessel CAD and a non-target lesion with FFR≤0.80 Age: ≥18 years (registered eligibility) | 7 weeks | Rosuvastatin 40 mg/day vs. standard lipid-lowering care | Intensive therapy: 79.1±25.3 to 58.4±26.3; standard therapy: 82.8±26.9 to 81.9±27.9 mg/dL (baseline to approximately 7 weeks). | NIRS-IVUS; FFR for lesion assessment | Median maxLCBI4mm change: −149.1 vs. +2.4 (P=0.01), favoring intensive therapy. |
| Atorvastatin | Komukai K et al. (2014) Japan^[20]^ | 70 patients with unstable angina and untreated dyslipidemia randomized Age: 20–75 years (registered eligibility) | 12 months | Atorvastatin 20 mg/day vs. 5 mg/day | Follow-up LDL-C: 69 vs. 78 mg/dL (P=0.039). | OCT | FCT increased 69% vs. 17% (P<0.001), favoring 20 mg/day. |
| Rosuvastatin | Park SJ et al. (2016) Korea^[21]^ | 312 patients randomized 2:1; 225 with serial IVUS Age: Rosuvastatin 40 mg: 62.6±9.3; 10 mg: 61.8±8.9 years (mean±SD; serial-IVUS population, n=152 and n=73). | 12 months | Rosuvastatin 40 mg/day vs. 10 mg/day | Rosuvastatin 40 mg: 105.3±32.8 to 59.1±22.2; 10 mg: 109.3±40.9 to 78.8±27.8 mg/dL (mean±SD; baseline to 12 months). | Grayscale IVUS and VH-IVUS | Pooled necrotic-core fraction decreased from 21.3% to 18.0%; normalized TAV decreased from 202.9 to 188.5 mm³. No significant dose-group difference in plaque composition. |
| Atorvastatin | Schartl M et al. (2001) Germany^[22]^ | 131 CAD patients randomized; atorvastatin n=65, usual care n=66 Age: Atorvastatin: 60.7±8.9; usual care: 59.8±7.2 years (mean±SD; n=65 and n=66). | 12 months | Atorvastatin titrated to LDL-C<100 mg/dL vs. usual lipid-lowering care | Atorvastatin: 155 to 86 mg/dL; usual care: 166 to 140 mg/dL. | Three-dimensional intracoronary ultrasound | Plaque-volume increase: +1.2±30.4 vs. +9.6±28.1 mm³ (P=0.191). Relative increase in hyperechogenicity index: 42.2% vs. 10.1% (P=0.021). |
| Atorvastatin | Kadoglou NP et al. (2010) Greece^[23]^ | 140 patients with moderate carotid stenosis; 70 per randomized group Age: Moderate-dose atorvastatin: 64.76±7.31; aggressive-dose: 63.24±6.76 years (mean±SD; analyzed population, n=65 and n=66). | 12 months | Atorvastatin 10–20 mg/day (target LDL-C<100) vs. 80 mg/day (target LDL-C<70 mg/dL) | Atorvastatin 10–20 mg: 162.12±33.62 to 104.67±20.32; 80 mg: 167.06±39.89 to 76.60±17.55 mg/dL (mean±SD; baseline to 12 months). | Carotid ultrasound (gray-scale median) | Echogenicity increased more with 80 mg/day: GSM 66.39 to 100.4 vs. 64.4 to 85.39 (P=0.024). Stenosis degree did not change. |
| Pravastatin | Takagi T et al. (1997) Japan^[24]^ | 36 men after coronary angioplasty with total cholesterol 200–260 mg/dL randomized (18 per group); 25 analyzed (pravastatin n=13, control n=12). Age: Pravastatin: 56±10; control: 56±11 years (analyzed population, n=13 and n=12). | 3 years | Pravastatin 10 mg once daily plus dietary/lifestyle intervention vs. dietary/lifestyle intervention alone. | Pravastatin: 4.57±0.51 to 3.41±0.69; control: 4.24±0.48 to 3.85±0.58 mmol/L (mean±SD; baseline to 3 years). | IVUS | Plaque-area change: −7±23% with pravastatin vs. +41±33% with control (P<0.0005); plaque-index change: −7±16% vs. +27±17% (P<0.0005). |
| Rosuvastatin | Underhill HR et al. (2008) United States^[25]^ | 43 patients with LDL-C 100–249 mg/dL and 16–79% carotid stenosis randomized; 33 with serial MRI Age: Low-dose rosuvastatin: 66.5±1.8; high-dose: 64.4±2.9 years (mean±SE; serial-MRI population, n=13 and n=20). | 24 months | Rosuvastatin 5 mg vs. high-dose rosuvastatin (40/80 mg) | LDL-C decreased 38.2% with low dose and 59.9% with high dose. | 1.5-T carotid MRI | Plaque volume did not change significantly. Among patients with baseline lipid-rich necrotic core, pooled necrotic-core proportion decreased by 41.4% (P=0.005). |
| Atorvastatin | Lo J et al. (2015) United States^[26]^ | 40 people with HIV and subclinical coronary atherosclerosis; atorvastatin n=19, placebo n=21 Age: Atorvastatin: 52.2±3.8; placebo: 50.0±5.6 years | 12 months | Atorvastatin 20 mg/day, increased to 40 mg/day if tolerated, vs. placebo | Mean change: atorvastatin −1.00 vs. placebo +0.30 mmol/L (P<0.0001). | Coronary CTA and aortic FDG-PET | Aortic inflammation primary endpoint was not different (P=0.77). Noncalcified coronary plaque volume changed −19.4% vs. +20.4% (P=0.009). |
| Pravastatin, Atorvastatin | Cannon CP et al. (2004) Multinational^[27]^ | 4,162 patients hospitalized with ACS within the preceding 10 days Age: Pravastatin: 58.3±11.3; atorvastatin: 58.1±11.2 years. | 18–36 months; mean 24 months | Atorvastatin 80 mg/day vs. pravastatin 40 mg/day | Median achieved LDL-C: 62 vs. 95 mg/dL. | Not applicable: clinical-event trial | No direct plaque-imaging endpoint. Two-year composite cardiovascular event rate: 22.4% vs. 26.3% (P=0.005), favoring atorvastatin. |
| Pitavastatin | Nakamura T et al. (2008) Japan^[28]^ | 65 ACS patients with echolucent carotid plaques; pitavastatin n=33, placebo n=32 Age: Pitavastatin: 60±9; placebo: 59±9 years (mean±SD; ACS population, n=33 and n=32). | 1 month | Pitavastatin 4 mg/day vs. placebo, started within 3 days after ACS | Pitavastatin: 164 to 102; placebo: 156 to 152 mg/dL (means; baseline to 1 month). | Carotid ultrasound with integrated backscatter | Calibrated backscatter improved from −18.7±3.3 to −12.7±2.3 dB with pitavastatin vs. −19.0±3.5 to −16.9±3.2 dB with placebo; follow-up between-group P<0.01. |
| Pitavastatin | Lu MT et al. (2024) United States^[29]^ | REPRIEVE randomized mechanistic substudy: 804 enrolled; 611 with paired CTA Age: 51±6 years (paired-CTA population) | 24 months | Pitavastatin calcium 4 mg/day vs. placebo | Mean change: −28.5 vs. −0.8 mg/dL. | Coronary CTA | Noncalcified plaque volume change: −1.7 vs. +2.6 mm³; baseline-adjusted difference −4.3 mm³ (P=0.04). Progression risk ratio 0.67 (P=0.003). |
| Pitavastatin, Pravastatin | Watanabe T et al. (2015) Japan^[30]^ | 20 high-risk patients randomized; 10 per group Age: Pitavastatin: 68.4±5.1; pravastatin: 63.9±11.2 years | 6 months | Pitavastatin 2 mg/day vs. pravastatin 10 mg/day | pitavastatin 150±21 to 80±16 mg/dL; pravastatin 142±24 to 103±18 mg/dL. | FDG-PET/CT, transesophageal and transthoracic ultrasound | Pitavastatin produced greater improvement in arterial inflammation (TBR), intima-media thickness and corrected integrated backscatter. |
| Pravastatin | Watanabe K et al. (2005) Japan^[31]^ | 60 nonhypercholesterolemic CAD patients; 30 per group Age: Pravastatin: 69.9±8.8; diet: 69.1±9.6 years | 6 months | Pravastatin 10 mg/day, increased to 20 mg/day to target LDL-C <100 mg/dL (26 patients on 10 mg and 4 on 20 mg), vs. ATP-III dietary therapy. | Pravastatin: 131±14 to 99±14 mg/dL; diet: 126±22 to 122±16 mg/dL. | Carotid ultrasound with integrated backscatter | Plaque echogenicity improved with pravastatin (−18.5 to −15.9 dB), not diet (−18.2 to −18.9 dB); plaque IMT did not regress significantly. |
| Pitavastatin | Nishiguchi T et al. (2018) Japan^[32]^ | ACS patients with untreated dyslipidemia; 53 with serial OCT Age: Median (IQR), years: early statin 66 (63–71); late statin 66 (62–74). | 3 and 36 weeks | Pitavastatin 4 mg/day immediately vs. starting 3 weeks later | Median (IQR), mg/dL, baseline / 3 weeks / 36 weeks: early statin 117 (105–129) / 63 (58–78) / 67 (63–78); late statin 118 (109–135) / 119 (105–138) / 76 (56–91). | OCT | At 3 weeks, FCT change was +8.3% vs. −5.8% (P<0.001). FCT increased comparably in both groups by 36 weeks. |
| Eicosapentaenoic acid added to statins | Niki T et al. (2016) Japan^[33]^ | 95 statin-treated patients randomized; 59 with serial plaque analysis (EPA 29, control 30) Age: EPA: 68.1±10.1; control: 69.4±10.7 years (imaging subset) | 6 months | EPA 1,800 mg/day added to existing strong statin vs. statin alone | EPA: 97.7±20.6 to 91.4±20.6 mg/dL; control: 100.0±31.1 to 88.3±19.2 mg/dL. | IB-IVUS | EPA-group lipid volume decreased from 18.5 to 15.0 mm³ (P=0.007); fibrous volume increased from 22.9 to 25.6 mm³ (P=0.01). These are within-group comparisons. |
| Pitavastatin, Ezetimibe | Hibi K et al. (2018) Japan^[34]^ | 128 statin-naive ACS patients randomized; 103 with serial IVUS Age: 63.2±10.8 years | 10 months | Pitavastatin 2 mg plus ezetimibe 10 mg/day vs. pitavastatin 2 mg/day | Combination: 123 to 64 mg/dL; monotherapy: 126 to 87 mg/dL. | IVUS and IB-IVUS | Relative plaque-volume change: −5.1% vs. −6.2% (P=0.66); lipid plaque-volume change: +4.3% vs. −3.0% (P=0.37). No incremental imaging benefit. |
| Pravastatin | Pitt B et al. (1995) United States^[35]^ | 408 patients with coronary artery disease and LDL-C ≥130 and <190 mg/dL despite diet; randomized / Age: mean 57 years | 3 years | Pravastatin vs. placebo | Baseline mean LDL-C 164 mg/dL. Pravastatin reduced total cholesterol by 19%, LDL-C by 28% and triglycerides by 8%, and increased HDL-C by 7% (P≤0.001 vs. placebo for all lipid variables). | Quantitative coronary arteriography | Progression of atherosclerosis reduced by 40% for minimal vessel diameter (P=0.04), particularly in lesions <50% stenosis at baseline; fewer new lesions with pravastatin (P≤0.03). Myocardial infarction reduced (8 vs. 17; P≤0.05). |
| **Cholesterol absorption inhibitor** | | | | | | | |
| Ezetimibe, Rosuvastatin | Wang X et al. (2016) China^[36]^ | 106 patients with CAD and hyperlipidemia; combination n=55, monotherapy n=51 Age: Ezetimibe/rosuvastatin: 63±10; rosuvastatin: 65±12 years (98 analyzed patients) | 12 months | Ezetimibe 10 mg/day plus rosuvastatin 10 mg/day vs. rosuvastatin 10 mg/day | Ezetimibe/rosuvastatin: 3.62±1.18 to 1.37±0.83 mmol/L; rosuvastatin: 3.48±1.26 to 1.85±0.79 mmol/L (baseline to 12 months). | IVUS | At 12 months, plaque burden, cross-sectional area and necrotic fraction were lower with combination therapy (P<0.05). |
| Atorvastatin, Ezetimibe | Oh PC et al. (2021) South Korea^[37]^ | 41 stable-angina patients randomized; 37 with serial imaging Age: Atorvastatin 10 mg + ezetimibe 10 mg: 56.3±7.1; atorvastatin 40 mg: 56.7±8.4 years (mean±SD; serial-imaging population, n=18 and n=19). | 12 months | Atorvastatin 10 mg plus ezetimibe 10 mg/day vs. atorvastatin 40 mg/day | Combination: 107 to 61 mg/dL; atorvastatin 40 mg: 101 to 58 mg/dL. | NIRS-IVUS | PAV change: −2.9 vs. −3.2 percentage points; combination met the prespecified noninferiority margin. LCBI did not decrease significantly in either group. |
| Atorvastatin, Ezetimibe | Ueda Y et al. (2017) Japan^[38]^ | 131 stable-CAD patients with yellow plaques randomized Age: 69±10 years (108 analyzed patients) | 9 months | Atorvastatin 10–20 mg/day plus ezetimibe 10 mg/day vs. atorvastatin 10–20 mg/day | Combination: 103±28 to 63±18 mg/dL; monotherapy: 100±28 to 75±17 mg/dL. | Coronary angioscopy and IVUS | Yellow-plaque grade improved similarly in both groups. Plaque-volume percentage decreased within the combination group; this alone does not establish a between-group benefit. |
| Atorvastatin, Ezetimibe | Kovarnik T et al. (2012) Czech Republic^[39]^ | 89 stable-angina patients analyzed after randomization Age: Intensive: 63.5±9.3; standard: 65.1±10.6 years | 12 months | Atorvastatin 80 mg plus ezetimibe 10 mg/day vs. standard lipid-lowering care | Intensive therapy: 3.1±1.3 to 2.0±0.8 mmol/L; standard therapy: 2.7±0.8 to 2.6±0.8 mmol/L. Percentage changes: −28.6±33.8% vs. −1.9±29.8% (P=0.0002). | IVUS and VH-IVUS | PAV change: −0.4 vs. +1.4 percentage points (P=0.014). No significant between-group difference in plaque composition. |
| Rosuvastatin, Ezetimibe | Masuda J et al. (2015) Japan^[40]^ | 51 stable-CAD patients randomized; 40 with serial IVUS Age: Combination: 64.0±7.9; monotherapy: 70.2±7.6 years (imaging subset) | 6 months | Rosuvastatin 5 mg plus ezetimibe 10 mg/day vs. rosuvastatin 5 mg/day | LDL-C reduction: 55.8% vs. 36.8% (P=0.004). | IVUS | Relative plaque-volume change: −13.2% vs. −3.1% (P=0.050). Group-by-time interaction P=0.021. |
| Statin, Ezetimibe | Nakano Y et al. (2023) Japan^[41]^ | CuVIC randomized IVUS substudy: 79 patients; statin 39, combination 40 Age: Median (IQR): statin 69 (63–74); combination 65 (58.8–72.8) years | 6–8 months | Statin plus ezetimibe 10 mg/day vs. statin alone | Median LDL-C: statin 96 to 83.5 mg/dL; combination 81 to 62.5 mg/dL. | IVUS and IB-IVUS | Relative plaque-volume change: combination −6.14% vs. statin −1.18% (P=0.042). |
| Atorvastatin, Ezetimibe | Tsujita K et al. (2015) Japan^[42]^ | 246 patients undergoing PCI randomized; 202 with serial IVUS Age: 30–85 years (registered eligibility) | 9–12 months | Atorvastatin titrated to LDL-C<70 mg/dL, with or without ezetimibe 10 mg/day | Follow-up: combination 63.2±16.3 vs. atorvastatin alone 73.3±20.3 mg/dL. | IVUS | PAV change: −1.4 vs. −0.3 percentage points (P=0.001). Plaque regression occurred in 78% vs. 58% (P=0.004). |
| Ezetimibe, Atorvastatin | Hougaard M et al. (2017) Denmark^[43]^ | 87 first-time STEMI patients randomized 1:1 Age: Ezetimibe: 55.3±11.0; placebo: 57.2±9.1 years (n=43 and n=44; both groups received atorvastatin). | 12 months | Ezetimibe 10 mg/day vs. placebo, both with atorvastatin 80 mg/day | Ezetimibe + atorvastatin: 3.7±0.7 to 1.4±0.8; placebo + atorvastatin: 4.1±0.9 to 2.0±0.5 mmol/L (mean±SD; baseline to 12 months; n=39 and n=41). | IVUS | No significant between-group change in necrotic-core volume (P=0.35), TAV (P=0.56), or PAV (P=0.91). |
| Ezetimibe, Fluvastatin | Habara M et al. (2014) Japan^[44]^ | 63 angina patients enrolled; 57 completed serial OCT Age: Ezetimibe + fluvastatin: 69.8±7.8; fluvastatin alone: 68.8±7.8 years (enrolled population, n=32 and n=31). | 9 months | Fluvastatin 30 mg plus ezetimibe 10 mg/day vs. fluvastatin 30 mg/day | Change: −34.0±32.0 vs. −8.3±17.4 mg/dL (P<0.001). | OCT | FCT increased by 0.08±0.08 vs. 0.04±0.06 mm (P<0.001), favoring combination therapy. |
| Simvastatin, Ezetimibe | Rossebø AB et al. (2008) Seven European countries^[45]^ | 1,873 patients with asymptomatic mild-to-moderate aortic valve stenosis Age: 67.5 years (overall mean; registered results) | Median 52.2 months | Simvastatin 40 mg plus ezetimibe 10 mg/day vs. placebo | Baseline LDL-C: ezetimibe/simvastatin 3.62±0.93; placebo 3.59±0.91 mmol/L. Time-weighted mean changes during follow-up: −53.8±18.7% vs. −3.8±20.6% (not final-visit changes). | Echocardiography for valve disease; no direct plaque-imaging endpoint | Primary composite outcome not reduced (HR 0.96; P=0.59). Ischemic cardiovascular events were reduced (HR 0.78; P=0.02); no reduction in valve-related events. |
| Ezetimibe, Simvastatin | Davidson MH et al. (2002) United States^[46]^ | Patients with primary hypercholesterolemia; baseline LDL-C 145–250 mg/dL Age: Mean (range), years: placebo 58.8 (25–84); ezetimibe 10 mg 60.3 (35–84); pooled simvastatin 56.4 (25–87); ezetimibe plus pooled simvastatin 57.6 (27–83). | 12 weeks | Ten randomized arms: ezetimibe 10 mg; simvastatin 10/20/40/80 mg; their corresponding combinations; placebo | Combination therapy reduced LDL-C by 44–57%, depending on simvastatin dose; pooled incremental reduction 13.8 percentage points vs. simvastatin alone. | Not applicable: lipid-efficacy trial | No direct plaque-imaging outcome; assessed lipid efficacy and safety. |
| Simvastatin + Ezetimibe | Baigent C et al. (2011) Multinational^[47]^ | 9,270 CKD patients; 3,023 on dialysis, 6,247 not on dialysis Age: 62±12 years | Median 4.9 years | Simvastatin 20 mg plus ezetimibe 10 mg/day vs. placebo | Average between-group LDL-C difference: 0.85 mmol/L (SE 0.02). | Not applicable: clinical-event trial | Major atherosclerotic events: 11.3% vs. 13.4%; RR 0.83 (95% CI 0.74–0.94), P=0.0021. No direct plaque-imaging endpoint. |
| Ezetimibe, Simvastatin | Cannon CP et al. (2015) Multinational^[48]^ | 18,144 patients hospitalized for ACS within the preceding 10 days; randomized, double-blind / Age: 18–100 years (registered eligibility) | Median 6 years | Simvastatin 40 mg plus ezetimibe 10 mg/day vs. simvastatin 40 mg/day plus placebo | Median time-weighted average LDL-C: 53.7 vs. 69.5 mg/dL (P<0.001). | Not applicable: clinical-event trial | No direct plaque-imaging endpoint. Seven-year Kaplan–Meier rate for the primary composite endpoint: 32.7% vs. 34.7% (absolute risk difference 2.0 percentage points; HR 0.936; 95% CI 0.89–0.99; P=0.016). |
| Rosuvastatin, Ezetimibe | Kim BK et al. (2022) South Korea^[49]^ | 3,780 patients with ASCVD; randomised, open-label, non-inferiority trial / Age: 19–80 years (registered eligibility) | 3 years | Rosuvastatin 10 mg plus ezetimibe 10 mg/day vs. rosuvastatin 20 mg/day | Combination therapy achieved LDL-C targets more often with a lower statin dose; LDL-C lowering was comparable between groups. | Not applicable: clinical-event trial | No direct plaque-imaging endpoint. Moderate-intensity statin plus ezetimibe was non-inferior to high-intensity statin monotherapy for the 3-year composite of cardiovascular death, major cardiovascular events or non-fatal stroke (non-inferiority margin 2.0%). |
| **PCSK9 inhibitor** | | | | | | | |
| Evolocumab | Nicholls SJ et al. (2016) Multinational^[50]^ | 968 statin-treated CAD patients randomized; 846 with follow-up IVUS Age: 59.8±9.2 years | 76 weeks of treatment; IVUS at week 78 | Evolocumab 420 mg subcutaneously monthly vs. placebo, added to statins | Mean baseline LDL-C 92.5±27.2 mg/dL; time-weighted treatment LDL-C 36.6 vs. 93.0 mg/dL. | IVUS | PAV change: −0.95 vs. +0.05 percentage points; TAV change: −5.8 vs. −0.9 mm³ (both P<0.001). |
| Evolocumab | Nicholls SJ et al. (2022) Multinational^[51]^ | 161 NSTEMI patients treated after randomization; 135 with serial imaging Age: 60.5±9.6 years | 52 weeks | Evolocumab 420 mg monthly vs. placebo, on statin therapy | Overall baseline 141.3±33.1 mg/dL; achieved LDL-C 28.1 vs. 87.2 mg/dL. | OCT and IVUS | Minimum FCT change: +42.7 vs. +21.5 µm (P=0.015); PAV change: −2.29 vs. −0.61 percentage points (P=0.009). |
| Evolocumab | Nicholls SJ et al. (2018) Multinational^[52]^ | GLAGOV randomized substudy: 331 patients with evaluable radiofrequency imaging Age: 18–99 years (registered eligibility) | 76 weeks of treatment | Evolocumab 420 mg monthly vs. placebo, added to statins | Achieved LDL-C: 33.5 vs. 89.9 mg/dL (P<0.0001). | IVUS with radiofrequency plaque-composition analysis | PAV change: −1.2 vs. +0.17 percentage points. No significant between-group changes in calcium, fibrous, fibrofatty or necrotic components. |
| Alirocumab added to statins | Ako J et al. (2019) Japan^[53]^ | 206 Japanese ACS patients randomized; 182 in the modified intention-to-treat imaging set Age: Alirocumab: 61.8±10.2; standard care: 60.5±11.6 years (imaging set) | 36 weeks | Alirocumab 75 mg every 2 weeks, increased to 150 mg if needed, plus statins vs. standard care | LDL-C reduction: alirocumab 63.9% vs. standard care 13.4%. | IVUS | Normalized TAV change: −4.8% vs. −3.1% (P=0.23); PAV change: −1.4 vs. −1.3 percentage points (P=0.79). No significant incremental plaque regression. |
| Alirocumab added to rosuvastatin | Räber L et al. (2022) Switzerland, Austria, Denmark and the Netherlands^[54]^ | 300 acute-MI patients randomized; 265 with serial IVUS Age: 58.5±9.7 years | 52 weeks | Alirocumab 150 mg subcutaneously every 2 weeks vs. placebo, both with rosuvastatin 20 mg/day | Overall baseline LDL-C 152.4±33.8 mg/dL; follow-up alirocumab 23.6 vs. placebo 74.4 mg/dL. | IVUS, NIRS and OCT | PAV change −2.13 vs. −0.92 percentage points (P<0.001); maxLCBI4mm change −79.42 vs. −37.60 (P=0.006); minimum FCT change +62.67 vs. +33.19 µm (P=0.001). |
| Alirocumab | Gao F et al. (2021) China^[55]^ | 61 CAD patients in serial OCT analysis; alirocumab n=30, standard care n=31 Age: Alirocumab: 61.3±8.9; standard care: 61.3±9.9 years | 36 weeks | Alirocumab 75 mg every 2 weeks plus atorvastatin 20 mg/day or rosuvastatin 10 mg/day vs. standard care | Alirocumab: 3.04±0.78 to 1.32±0.39 mmol/L; standard care: 3.18±0.97 to 2.22±0.69 mmol/L. | OCT | Minimum FCT increase: 18.0 vs. 13.2 µm (P=0.029); maximum lipid-arc reduction: 15.1° vs. 8.4° (P=0.008). |
| Alirocumab, rosuvastatin | Sugizaki Y et al. (2020) Japan^[56,57]^ | 24 patients with LDL-C>70 mg/dL despite statins; 12 per group Age: Alirocumab: 69 (61–78); control: 72 (68–77) years; median (IQR). | 36 weeks | Alirocumab 75 mg every 2 weeks plus rosuvastatin 10 mg/day vs. rosuvastatin 10 mg/day | Median LDL-C: alirocumab 90 to 27 mg/dL; control 84 to 71 mg/dL. | OCT | Addition of alirocumab produced greater increases in minimum fibrous-cap thickness and greater reductions in lipid index and macrophage grade than rosuvastatin alone. |
| **ACAT inhibitors** | | | | | | | |
| Avasimibe | Tardif JC et al. (2004) Multinational^[58]^ | Patients with coronary atherosclerosis receiving background lipid-lowering therapy Age: Placebo: 58±10; avasimibe 50 mg: 58.6±10; 250 mg: 58±9; 750 mg: 58.7±9 years (mean±SD; n=154, 157, 164, 164). | Up to 24 months | Avasimibe 50, 250, or 750 mg/day vs. placebo | LDL-C increased 7.8%, 9.1%, and 10.9% with avasimibe vs. 1.7% with placebo. | IVUS and coronary angiography | No beneficial effect on plaque progression. TAV change: +7.7/+4.1/+4.8 mm³ vs. +0.7 mm³; PAV change: +0.7/+0.8/+1.0 vs. +0.4 percentage points. |
| **High-density lipoprotein mimetics** | | | | | | | |
| MDCO-216 | Nicholls SJ et al. (2018) Canada and Europe^[59]^ | 126 ACS patients randomized; 122 received study drug; 113 with serial IVUS Age: 61.8±10.4 years (treated population) | Day 36 imaging after five weekly infusions | MDCO-216 20 mg/kg intravenously weekly for 5 doses vs. placebo, with statins | MDCO-216: 93.8 to 70.5 mg/dL; placebo: 84.1 to 68.6 mg/dL. | IVUS | PAV change: −0.21 vs. −0.94 percentage points (P=0.07); TAV change: −6.4 vs. −7.9 mm³ (P=0.67). No incremental plaque regression. |
| **Apolipoprotein C-III inhibitor** | | | | | | | |
| Olezarsen | Marston NA et al. (2026) Multinational^[60]^ | 468 participants with largely moderate hypertriglyceridemia, ASCVD or high cardiovascular risk, and noncalcified plaque on baseline coronary CTA; prespecified imaging substudy of the randomized, placebo-controlled Essence-TIMI 73b trial / Age: median 63 years | 12 months (imaging); 6-month biomarker analysis | Olezarsen vs. placebo, on background lipid-lowering therapy | Placebo-adjusted reductions at 6 months: triglycerides −63.9%, remnant cholesterol −71.9%, apoB −16.0%; no difference in LDL-C. | Coronary CTA (noncalcified plaque volume) | Placebo-adjusted least-squares mean difference in 12-month noncalcified plaque volume: 2.98% (95% CI −3.4 to 9.3; P=0.36). No significant differences in low-attenuation, calcified or total plaque volume. Substantial triglyceride and remnant cholesterol lowering did not affect plaque. |
| **ATP citrate lyase inhibitor** | | | | | | | |
| Bempedoic acid | Nissen SE et al. (2023) Multinational^[61]^ | 13,970 statin-intolerant patients with, or at high risk for, cardiovascular disease; randomized, double-blind, placebo-controlled | Median 40.6 months | Bempedoic acid 180 mg/day vs. placebo, on background lipid-lowering therapy | Bempedoic acid lowered LDL-C by approximately 20% versus placebo. | Not applicable: clinical-event trial | No direct plaque-imaging endpoint. Four-component MACE (cardiovascular death, nonfatal myocardial infarction, nonfatal stroke, or coronary revascularization) occurred in 11.7% vs. 13.3% (HR 0.87; 95% CI 0.79–0.96; P=0.004). |
| **Oxidized LDL receptor antagonist** | | | | | | | |
| MEDI6570 (anti-LOX-1) | O’Donoghue ML et al. (2025) Multinational^[62]^ | Patients with residual inflammation after myocardial infarction; randomized, double-blind, dose-finding phase 2 trial | Per protocol (imaging follow-up) | MEDI6570 (antibody against lectin-like oxidized LDL receptor-1) vs. placebo | Not applicable: lipid levels were not the treatment target. | Coronary CTA (noncalcified plaque volume) | MEDI6570 significantly reduced soluble LOX-1 and IL-6 levels but did not reduce noncalcified coronary plaque volume in patients with residual inflammation after acute myocardial infarction. |
| **Antidiabetic drugs** | | | | | | | |
| **Biguanides** | | | | | | | |
| Metformin | UKPDS Group (1998) United Kingdom^[63]^ | 753 overweight patients with newly diagnosed type 2 diabetes; randomized controlled trial (UKPDS 34) | Median 10.7 years | Metformin vs. conventional treatment | Not applicable: glucose-lowering trial. | Not applicable: clinical-event trial | No direct plaque-imaging endpoint. Risk of macrovascular disease was 30% lower and myocardial infarction 39% lower with metformin than with conventional treatment; the reduction persisted through 10 years of post-trial follow-up. |
| **SGLT2 inhibitors** | | | | | | | |
| Tofogliflozin | Katakami N et al. (2022) Japan^[64]^ | Type 2 diabetes patients without apparent cardiovascular disease; post hoc analysis of the UTOPIA trial (multicentre, prospective, randomized, open-label, blinded-endpoint); tofogliflozin n=168, conventional treatment n=169 | 104 weeks | Tofogliflozin vs. conventional treatment | Not applicable: glucose-lowering trial. | Carotid ultrasound (gray-scale median, an index of ultrasonic tissue characteristics of the carotid wall) | Mean carotid gray-scale median did not change significantly in either group and did not differ between tofogliflozin and conventional treatment (mean difference −1.24 [95% CI −3.87 to 1.38]; P=0.35). No effect of SGLT2 inhibition on carotid wall tissue characteristics was detected. |
| **DPP-4 inhibitors** | | | | | | | |
| Sitagliptin | Kuramitsu S et al. (2017) Japan^[65]^ | 41 patients with diabetes and ACS; sitagliptin n=21, control n=20 Age: Sitagliptin: 63.8±9.5; control: 65.3±5.8 years (serial-IVUS population, n=21 and n=20). | 6 months | Sitagliptin 50–100 mg/day plus diet/exercise vs. diet/exercise | Sitagliptin: 112.4±34.2 to 79.0±19.8; control: 99.0±34.4 to 79.9±22.4 mg/dL (baseline to 6 months). | IVUS and IB-IVUS | Relative plaque-volume change: −4.0±8.5% vs. −1.4±8.8% (P=0.35). Lipid plaque-volume change: −7.1±21.5% vs. +15.6±41.8% (P=0.03). |
| Sitagliptin | Li B et al. (2020) China^[66]^ | 149 CAD patients with T2DM randomized; 88 completed serial 3D-QCA Age: Sitagliptin: 60.55±9.54; control: 59.65±10.30 years (mean±SD; randomized population, n=75 and n=74). | 18 months | Sitagliptin 100 mg/day vs. conventional therapy initially using acarbose 50 mg three times daily; metformin and/or insulin added as needed after 3 months. Both groups received atorvastatin 20 mg/day and aspirin 100 mg/day. | Sitagliptin: 3.01 (2.23–3.71) to 2.46±0.49; control: 2.86 (2.23–3.43) to 2.47±0.61 mmol/L. Baseline: median (IQR). final visit: mean±SD (18 months; n=44 per group). | Three-dimensional quantitative coronary angiography | PAV change: +1.69 vs. +5.12 percentage points (P=0.023). No significant difference in late lumen loss. |
| Vildagliptin | Yamamoto H et al. (2021) Japan^[67]^ | 20 CAD patients with impaired glucose tolerance; 10 per group; 47 lesions Age: Vildagliptin: 65.9±10.6; control: 68.3±8.1 years | 6 months | Vildagliptin 50 mg/day vs. no glucose-lowering medication; both received conventional management | Baseline: vildagliptin 84.0±14.3 vs. control 82.9±27.0 mg/dL. Group-specific follow-up LDL-C: unavailable. | OCT | Adjusted minimum FCT difference +42.7 µm (P=0.0022); lipid-arc difference −22.8°. |
| Sitagliptin | Nozue T et al. (2016) Japan^[68]^ | 28 T2DM patients randomized; 26 analyzed (sitagliptin 15, control 11) Age: Sitagliptin: 72±6; control: 69±10 years | 48 weeks | Sitagliptin 50 mg/day vs. conventional glucose-lowering treatment | Sitagliptin: 99±33 to 81±23 mg/dL; control: 106±25 to 94±20 mg/dL. | Grayscale IVUS and IB-IVUS | Plaque-volume change: −1.7±8.5% vs. −3.2±12.2%; no significant between-group difference. Lipid volume decreased within the sitagliptin group. |
| **Other antidiabetic drugs** | | | | | | | |
| Pioglitazone, Glimepiride | Nissen SE et al. (2008) North and South America^[69]^ | 543 CAD patients with T2DM randomized; 360 with serial IVUS Age: Pioglitazone: 60.0±9.4; glimepiride: 59.7±9.1 years (randomized population) | 18 months | Pioglitazone 15–45 mg/day vs. glimepiride 1–4 mg/day, titrated as tolerated | Baseline to mean on-treatment LDL-C: pioglitazone 93.5±30.7 to 95.6±28.9 mg/dL; glimepiride 94.4±32.9 to 96.1±30.4 mg/dL (360 completers). | IVUS | PAV change: pioglitazone −0.16 vs. glimepiride +0.73 percentage points (P=0.002). |
| Rosiglitazone | Gerstein HC et al. (2010) Multinational^[70]^ | 672 patients with T2DM and coronary atherosclerosis randomized Age: 30–80 years (eligibility) | 18 months | Rosiglitazone vs. glipizide; titrated regimens | Adjusted LDL-C change from baseline to month 18: rosiglitazone −1.237%; glipizide −11.600% (registered results; geometric mean estimates). | IVUS | Primary PAV difference −0.64 percentage points (95% CI −1.46 to 0.17; P=0.12). Secondary normalized TAV difference −5.1 mm³ (P=0.04). |
| **Anti-inflammatory drug category** | | | | | | | |
| Colchicine | Psaltis PJ et al. (2025) Australia^[71]^ | 64 NSTEMI patients randomized; 57 with serial OCT Age: Median 61.5 years | Median 17.8 months | Colchicine 0.5 mg/day vs. placebo, added to guideline-directed therapy | Colchicine: 3.0±1.2 to 1.6±0.5 mmol/L; placebo: 3.1±1.3 to 1.8±0.9 mmol/L (paired samples from 56 completers). | OCT | Primary minimum FCT change was not significantly different (P=0.18). Cap rupture: 3.6% vs. 27.6% (P=0.03). Longer-follow-up subgroup findings were post hoc. |
| Canakinumab | Ridker PM et al. (2017) Multinational^[72]^ | 10,061 patients with prior MI and hsCRP≥2 mg/L Age: ≥18 years (registered eligibility) | Median 3.7 years | Canakinumab 50, 150, or 300 mg subcutaneously every 3 months vs. placebo | Canakinumab did not reduce lipid levels from baseline. | Not applicable: clinical-event trial | No direct plaque-imaging endpoint. The 150-mg dose reduced recurrent cardiovascular events (HR 0.85; P=0.021), meeting the multiplicity-adjusted threshold. |
| Colchicine | Yu M et al. (2024) China^[73]^ | 128 ACS patients with lipid-rich plaques randomized; 104 completed (52 per group) Age: 58.0±9.8 years | 12 months | Colchicine 0.5 mg/day vs. placebo | Placebo: 81.1±30.9 to 61.8±23.2; colchicine: 92.6±42.5 to 57.9±23.2 mg/dL (baseline to 12 months; n=64 per group at baseline and n=52 per group at follow-up). | OCT | Minimum FCT increased 87.2 µm with colchicine vs. 51.9 µm with placebo; adjusted difference 34.2 µm (P=0.006). Mean lipid-arc reduction was greater with colchicine (P=0.004). |
| Colchicine | Tardif JC et al. (2019) Multinational^[74]^ | 4,745 patients recruited within 30 days after myocardial infarction; randomized, double-blind, placebo-controlled / Age: ≥18 years (registered eligibility) | Median 22.6 months | Colchicine 0.5 mg/day vs. placebo | Not applicable: no lipid endpoint reported. | Not applicable: clinical-event trial | No direct plaque-imaging endpoint. Primary composite endpoint: 5.5% vs. 7.1% (HR 0.77; 95% CI 0.61–0.96; P=0.02); stroke HR 0.26 (95% CI 0.10–0.70). Diarrhea 9.7% vs. 8.9%; pneumonia as a serious adverse event 0.9% vs. 0.4% (P=0.03). |
| Colchicine | Nidorf SM et al. (2020) Australia and the Netherlands^[75]^ | 5,522 patients with chronic coronary disease; randomized, controlled, double-blind / Age: ≥18 years (registered eligibility) | Median 28.6 months | Colchicine 0.5 mg/day vs. placebo | Not applicable: no lipid endpoint reported. | Not applicable: clinical-event trial | No direct plaque-imaging endpoint. Primary composite endpoint: 6.8% vs. 9.6% (HR 0.69; 95% CI 0.57–0.83; P<0.001); key secondary endpoint HR 0.72 (95% CI 0.57–0.92; P=0.007). Non-cardiovascular death was numerically higher with colchicine (HR 1.51; 95% CI 0.99–2.31). |
| Colchicine | Jolly SS et al. (2025) Multinational^[76]^ | 7,062 patients with myocardial infarction; randomized, double-blind, placebo-controlled / Age: ≥18 years (registered eligibility) | Median 3 years | Colchicine vs. placebo, started soon after myocardial infarction, on background guideline-directed therapy | C-reactive protein difference at 3 months (colchicine minus placebo), adjusted for baseline: −1.28 mg/L (95% CI −1.81 to −0.75). | Not applicable: clinical-event trial | No direct plaque-imaging endpoint. Primary composite endpoint: 9.1% vs. 9.3% (HR 0.99; 95% CI 0.85–1.16; P=0.93). Diarrhea 10.2% vs. 6.6% (P<0.001); serious infections did not differ. Marked CRP reduction without clinical benefit. |
| **Antihypertensive drugs** | | | | | | | |
| Aliskiren | Puri R et al. (2015) Multinational^[77]^ | AQUARIUS randomized-trial post hoc analysis: 115 diabetic and 343 nondiabetic patients Age: Diabetes: aliskiren 60.1±9.0, placebo 61.0±7.0; no diabetes: aliskiren 59.8±9.0, placebo 58.3±8.7 years (mean±SD). | 104 weeks | Aliskiren 300 mg/day vs. placebo | Diabetes: aliskiren 78 (70–97) to 82 (69–101), placebo 80 (60–95) to 82 (68–102); no diabetes: aliskiren 81 (70–104) to 96 (78–113), placebo 85 (70–109) to 91 (77–111) mg/dL (median [IQR]; baseline to on-treatment follow-up). | IVUS | Treatment effects differed by diabetes status. Nondiabetic patients showed greater regression; diabetic subgroup findings were inconclusive and hypothesis-generating. |
| Amlodipine, enalapril | Nissen SE et al. (2004) Multinational^[78]^ | 1,997 CAD patients randomized; 1,991 included in efficacy analyses; 274 completed the IVUS substudy Age: Amlodipine: 57.3±9.7; enalapril: 58.5±9.9; placebo: 57.2±9.5 years (efficacy population) | 24 months | Amlodipine 10 mg/day vs. enalapril 20 mg/day vs. placebo | Baseline LDL-C: amlodipine 104±32; enalapril 101±31; placebo 100±32 mg/dL. Group-specific follow-up LDL-C: unavailable. | IVUS substudy | Amlodipine reduced cardiovascular events vs. placebo (HR 0.69; P=0.003). IVUS trend toward less progression was not significant overall (P=0.12). |
| Olmesartan | Hirohata A et al. (2010) Japan^[79]^ | 247 patients with stable angina and CAD randomized Age: Control: 68.4±8.8; olmesartan: 67.8±8.7 years. | 14 months | Olmesartan 10–40 mg/day plus conventional therapy vs. conventional therapy | Baseline LDL-C: control 107.0±30.2; olmesartan 103.8±24.8 mg/dL. Group-specific follow-up LDL-C: unavailable. | IVUS | Relative TAV change: control +5.4% vs. olmesartan +0.6%; relative PAV change: +3.1% vs. −0.7% (both P<0.05). |
| Lacidipine, Atenolol | Zanchetti A et al. (2002) Multinational^[80]^ | 2,334 patients with hypertension; randomized, double-blind / Age: 45–75 years (registered eligibility) | 4 years | Lacidipine-based vs. atenolol-based antihypertensive therapy | Not applicable: antihypertensive comparison; no between-group lipid difference was reported. | B-mode carotid ultrasound (IMT and plaque number) | Four-year CBM(max) progression difference −0.0227 mm (intention-to-treat) and −0.0281 mm (completers), favoring lacidipine (P<0.0001); yearly IMT progression 0.0145 vs. 0.0087 mm/year (40% reduction; P=0.0073). Plaque progression was less common and regression more common with lacidipine. Clinic blood pressure reduction was identical; no significant difference in cardiovascular events. |
| Nifedipine | Luscher TF et al. (2009) Multinational^[81]^ | 454 patients with stable coronary artery disease undergoing PCI; randomized, double-blind, placebo-controlled | 18–24 months | Nifedipine GITS 30–60 mg/day vs. placebo, on top of statin therapy | LDL-C lower on nifedipine by 4.8 mg/dL (P=0.495); HDL-C higher by 3.6 mg/dL (P=0.026). | Intravascular ultrasound (plaque volume) and coronary endothelial function | Percentage change in coronary plaque volume: 1.0% with nifedipine vs. 1.9% with placebo (not significant). Nifedipine improved coronary endothelial function. |
| Nifedipine | Lichtlen PR et al. (1990) Multinational^[82]^ | 425 patients with mild coronary artery disease on arteriography; randomized to nifedipine or placebo; 348 underwent repeat arteriography | 3 years | Nifedipine 80 mg/day vs. placebo | Not applicable: angiographic progression trial. | Quantitative coronary arteriography | No significant difference in progression or regression of existing lesions. The number of new lesions per patient was significantly lower with nifedipine (0.59 vs. 0.82 lesions per patient; 28% reduction). |
| Valsartan | Yano H et al. (2012) Japan^[83]^ | 116 patients with acute myocardial infarction randomly assigned to valsartan plus captopril or captopril alone | Follow-up IVUS after 6–12 months (per protocol) | Valsartan plus captopril vs. captopril alone | Not reported as the study focus. | Intravascular ultrasound (non-culprit intermediate lesions) | Nominal change in percent atheroma volume: −1.9% (combination) vs. −0.68% (ACEI alone); P=0.06. Percent change in lumen volume +4.3% vs. −0.3% (P=0.03), indicating coronary artery enlargement rather than plaque regression. |
| Perindopril | Rodriguez-Granillo GA et al. (2007) Multinational^[84]^ | 118 matched evaluable IVUS examinations (711 matched 5-mm segments) from a double-blind, multicentre trial substudy | Median 3.0 years (IQR 1.9–4.1) | Perindopril 8 mg/day vs. placebo | Not applicable: substudy of a hypertension/heart failure trial. | Intravascular ultrasound (plaque and vessel cross-sectional area) | No significant difference in change in plaque CSA (P=0.27). Vessel CSA differed (P=0.04) with more frequent constrictive remodelling on perindopril (34% vs. 25%; P=0.01). |
| Ramipril | MacMahon S et al. (2000) Multinational^[85]^ | 617 patients with coronary or other occlusive arterial disease; randomized, placebo-controlled; carotid atherosclerosis assessed by B-mode ultrasound | 4 years (assessments at baseline, 2 and 4 years) | Ramipril 5–10 mg/day vs. placebo | Not reported as a treatment target; blood pressure reduced by 6/4 mm Hg vs. placebo (P<0.001). | B-mode carotid ultrasound (wall thickness and carotid plaque) | No difference between groups in common carotid wall thickness (P=0.58) or in carotid plaque (P=0.93). Left ventricular mass index decreased by 3.8 g/m² with ramipril (P=0.04). |
| Azelnidipine, Amlodipine | Kojima T et al. (2011) Japan^[86]^ | 199 hypertensive patients scheduled for coronary intervention; 115 with evaluable serial IVUS; prospective, randomized, open-label | 48 weeks | Azelnidipine 16 mg/day vs. amlodipine 5 mg/day | Lipid profiles comparable between groups (LDL-C 97 mg/dL). | Intravascular ultrasound (coronary plaque volume) | Percent change in plaque volume: −4.67% (azelnidipine) vs. −4.85% (amlodipine); azelnidipine was non-inferior (upper 95% CI of the difference 0.18%, below the 6.525% margin). |
| **Other drugs** | | | | | | | |
| Icosapent ethyl | Bhatt DL et al. (2019) Multinational^[87]^ | 8,179 patients with established cardiovascular disease or diabetes with additional risk factors, receiving statin therapy, with fasting triglycerides 135–499 mg/dL; randomized, double-blind, placebo-controlled / Age: ≥45 years (registered eligibility) | Median 4.9 years | Icosapent ethyl 2 g twice daily (4 g/day) vs. placebo | Eligible LDL-C 41–100 mg/dL; no significant between-group LDL-C difference. | Not applicable: clinical-event trial | No direct plaque-imaging endpoint. Primary composite endpoint: 17.2% vs. 22.0% (HR 0.75; 95% CI 0.68–0.83; P<0.001); key secondary endpoint 11.2% vs. 14.8% (HR 0.74). Hospitalization for atrial fibrillation or flutter 3.1% vs. 2.1% (P=0.004). |
| EPA and DHA | Alfaddagh A et al. (2017) United States^[88]^ | 285 statin-treated patients with stable CAD randomized Age: 63.0±7.7 years | 30 months | EPA 1.86 g plus DHA 1.5 g/day as omega-3 ethyl esters vs. no omega-3 | Analyzed baseline LDL-C: omega-3 78.5±27.0, control 77.5±27.5 mg/dL; changes did not differ significantly (P=0.76). | Coronary CTA | Primary noncalcified plaque-volume endpoint was not significantly different (intention-to-treat P=0.14). Fibrous-plaque progression was lower in the omega-3 group. |
| Icosapent ethyl | Budoff MJ et al. (2020) United States^[89]^ | 80 statin-treated patients with CAD and elevated triglycerides randomized; 68 with serial CT Age: Analyzed population: 57.4±8.7 years | 18 months; interim imaging at 9 months | Icosapent ethyl 4 g/day vs. mineral-oil placebo | No significant treatment-group difference in LDL-C change. | Coronary CTA (multidetector CT) | Low-attenuation plaque volume changed −17% with icosapent ethyl vs. +109% with placebo (P=0.0061). |
| Torcetrapib | Nissen SE et al. (2007) Multinational^[90]^ | 1,188 patients with coronary disease undergoing intravascular ultrasonography; 910 with paired IVUS; randomly assigned after an atorvastatin run-in / Age: 18–75 years (registered eligibility) | 24 months | Atorvastatin plus torcetrapib 60 mg/day vs. atorvastatin monotherapy | Torcetrapib produced an approximate 61% relative increase in HDL-C and a 20% relative decrease in LDL-C (LDL-C/HDL-C ratio <1.0). | Intravascular ultrasound (IVUS) | Percent atheroma volume increased by 0.12% with torcetrapib-atorvastatin vs. 0.19% with atorvastatin alone (P=0.72); normalized atheroma volume showed a small favorable effect (P=0.02); no significant difference in the most diseased segment. Systolic blood pressure increased by 4.6 mm Hg with torcetrapib. |
| Dalcetrapib | Fayad ZA et al. (2011) Canada, United States^[91]^ | 130 patients with or at high risk of CAD; dalcetrapib 64, placebo 66 Age: Dalcetrapib: 62.6±8.2; placebo: 64.6±7.8 years | 24 months for MRI; 6 months for primary PET/CT | Dalcetrapib 600 mg/day vs. placebo | No significant between-group LDL-C reduction; stable background lipid-lowering therapy. | Carotid/aortic MRI and FDG-PET/CT | Total vessel-area difference −4.01 mm² (90% CI −7.23 to −0.80; nominal P=0.04). Primary index-vessel inflammatory TBR did not differ. |
| apabetalone / RVX-208 | Nicholls SJ et al. (2016) Multinational^[92]^ | 323 patients with angiographic CAD and low HDL-C; randomized 3:1 Age: ≥18 years (registered eligibility) | 26 weeks | RVX-208 (apabetalone) 200 mg/day vs. placebo | LDL-C decreased 15.8% vs. 17.9% (P=0.55). | IVUS | PAV change: −0.40 vs. −0.30 percentage points (P=0.81); TAV change: −4.2 vs. −3.8 mm³ (P=0.86). No incremental regression. |
| Omega-3 polyunsaturated fatty acids added to statins | Ahn J et al. (2016) South Korea^[93]^ | 74 statin-treated CAD patients after PCI; omega-3 n=38, placebo n=36 Age: 18–80 years (eligibility) | 12 months | Omega-3 PUFA 3 g/day vs. placebo, added to statins | Omega-3: 127.00±29.42 to 86.81±51.77 mg/dL; placebo: 113.80±35.62 to 80.77±21.87 mg/dL. | IVUS | No significant between-group difference in atheroma-volume-index change (P=0.768) or relative PAV change (P=0.526). |
| SHR-1918 | Xie X et al. (2025) China^[94]^ | 333 patients with suboptimally controlled hyperlipidemia; randomized 4:1 within dose cohorts Age: 18–75 years (registered eligibility) | Initial treatment 16 weeks; extension regimens of 36 or 40 weeks | SHR-1918 150/300/600 mg every 4 weeks or 600 mg every 8 weeks vs. placebo | Placebo-adjusted LDL-C reductions: 21.7%, 27.3%, 29.9% (Q4W doses) and 22.5% (600 mg Q8W). | Not applicable: lipid-efficacy trial | No direct plaque-imaging endpoint; assessed lipid efficacy and safety. |

Abbreviations. ****ACAT****, acyl-coenzyme A:cholesterol acyltransferase; ****ACEI****, angiotensin-converting enzyme inhibitor; ****ACS****, acute coronary syndrome; ****ANOVA****, analysis of variance; ****apoB****, apolipoprotein B; ****ASCVD****, atherosclerotic cardiovascular disease; ****ATP-III****, Adult Treatment Panel III; ****B-mode****, brightness-mode; ****CAD****, coronary artery disease; ****CBM(max)****, mean of the maximum intima-media thicknesses of the far walls of the common carotid arteries and bifurcations; ****CI****, confidence interval; ****CKD****, chronic kidney disease; ****CRP****, C-reactive protein; ****CSA****, cross-sectional area; ****CT****, computed tomography; ****CTA****, computed tomography angiography; ****dB****, decibel; ****DHA****, docosahexaenoic acid; ****DPP-4****, dipeptidyl peptidase-4; ****EPA****, eicosapentaenoic acid; ****FCT****, fibrous cap thickness; ****FDG-PET/CT****, fluorodeoxyglucose positron emission tomography/computed tomography; ****FFR****, fractional flow reserve; ****GITS****, gastrointestinal therapeutic system; ****GSM****, gray-scale median; ****HDL-C****, high-density lipoprotein cholesterol; ****HIV****, human immunodeficiency virus; ****HR****, hazard ratio; ****hsCRP****, high-sensitivity C-reactive protein; ****IB-IVUS****, integrated backscatter intravascular ultrasound; ****IL-6****, interleukin-6; ****IMT****, intima-media thickness; ****IQR****, interquartile range; ****IVUS****, intravascular ultrasound; ****LCBI****, lipid-core burden index; ****LDL****, low-density lipoprotein; ****LDL-C****, low-density lipoprotein cholesterol; ****LOX-1****, lectin-like oxidized low-density lipoprotein receptor-1; ****MACE****, major adverse cardiovascular event; ****maxLCBI4mm****, maximum lipid-core burden index within a 4-mm segment; ****MDA-LDL****, malondialdehyde-modified low-density lipoprotein; ****MI****, myocardial infarction; ****MRI****, magnetic resonance imaging; ****NIRS****, near-infrared spectroscopy; ****NIRS-IVUS****, combined near-infrared spectroscopy and intravascular ultrasound; ****NSTEMI****, non-ST-segment elevation myocardial infarction; ****OCT****, optical coherence tomography; ****PAV****, percent atheroma volume; ****PCI****, percutaneous coronary intervention; ****PCSK9****, proprotein convertase subtilisin/kexin type 9; ****PET/CT****, positron emission tomography/computed tomography; ****PUFA****, polyunsaturated fatty acid; ****Q4W****, every 4 weeks; ****Q8W****, every 8 weeks; ****QCA****, quantitative coronary angiography; ****RR****, risk ratio; ****SD****, standard deviation; ****SE****, standard error; ****SGLT2****, sodium-glucose cotransporter 2; ****STEMI****, ST-segment elevation myocardial infarction; ****T2DM****, type 2 diabetes mellitus; ****TAV****, total atheroma volume; ****TBR****, target-to-background ratio; ****VH-IVUS****, virtual histology intravascular ultrasound.

# References

1.	Zhang X, Wang H, Liu S, et al. Intensive-dose atorvastatin regimen halts progression of atherosclerotic plaques in new-onset unstable angina with borderline vulnerable plaque lesions. J Cardiovasc Pharmacol Ther. 2013;18(2):119-125.

2.	Hiro T, Kimura T, Morimoto T, et al. Effect of intensive statin therapy on regression of coronary atherosclerosis in patients with acute coronary syndrome: a multicenter randomized trial evaluated by volumetric intravascular ultrasound using pitavastatin versus atorvastatin (JAPAN-ACS [Japan assessment of pitavastatin and atorvastatin in acute coronary syndrome] study). J Am Coll Cardiol. 2009;54(4):293-302.

3.	Nicholls SJ, Ballantyne CM, Barter PJ, et al. Effect of two intensive statin regimens on progression of coronary disease. N Engl J Med. 2011;365(22):2078-2087.

4.	Okazaki S, Yokoyama T, Miyauchi K, et al. Early statin treatment in patients with acute coronary syndrome: demonstration of the beneficial effect on atherosclerotic lesions by serial volumetric intravascular ultrasound analysis during half a year after coronary event: the ESTABLISH Study. Circulation. 2004;110(9):1061-1068.

5.	Tani S, Watanabe I, Anazawa T, et al. Effect of pravastatin on malondialdehyde-modified low-density lipoprotein levels and coronary plaque regression as determined by three-dimensional intravascular ultrasound. Am J Cardiol. 2005;96(8):1089-1094.

6.	Nissen SE, Tuzcu EM, Schoenhagen P, et al. Effect of intensive compared with moderate lipid-lowering therapy on progression of coronary atherosclerosis: a randomized controlled trial. JAMA. 2004;291(9):1071-1080.

7.	Toi T, Taguchi I, Yoneda S, et al. Early effect of lipid-lowering therapy with pitavastatin on regression of coronary atherosclerotic plaque. Comparison with atorvastatin. Circ J. 2009;73(8):1466-1472.

8.	Hong YJ, Jeong MH, Hachinohe D, et al. Comparison of effects of rosuvastatin and atorvastatin on plaque regression in Korean patients with untreated intermediate coronary stenosis. Circ J. 2011;75(2):398-406.

9.	Lee SWL, Hau WKT, Kong SL, et al. Virtual histology findings and effects of varying doses of atorvastatin on coronary plaque volume and composition in statin-naive patients: the VENUS study. Circ J. 2012;76(11):2662-2672.

10.	Matsushita K, Hibi K, Komura N, et al. Effects of 4 Statins on Regression of Coronary Plaque in Acute Coronary Syndrome. Circ J. 2016;80(7):1634-1643.

11.	Nozue T, Yamamoto S, Tohyama S, et al. Statin treatment for coronary artery plaque composition based on intravascular ultrasound radiofrequency data analysis. Am Heart J. 2012;163(2):191-199.e1.

12.	Thondapu V, Kurihara O, Yonetsu T, et al. Comparison of Rosuvastatin Versus Atorvastatin for Coronary Plaque Stabilization. Am J Cardiol. 2019;123(10):1565-1571.

13.	Yokoyama M, Komiyama N, Courtney BK, et al. Plasma low-density lipoprotein reduction and structural effects on coronary atherosclerotic plaques by atorvastatin as clinically assessed with intravascular ultrasound radio-frequency signal analysis: a randomized prospective study. Am Heart J. 2005;150(2):287.

14.	Lee CW, Kang SJ, Ahn JM, et al. Comparison of effects of atorvastatin (20 mg) versus rosuvastatin (10 mg) therapy on mild coronary atherosclerotic plaques (from the ARTMAP trial). Am J Cardiol. 2012;109(12):1700-1704.

15.	Egede R, Jensen LO, Hansen HS, Hansen KN, Junker A, Thayssen P. Influence of high-dose lipid lowering treatment compared to low-dose lipid lowering treatment on plaque composition assessed by intravascular ultrasound virtual histology in patients with ST-segment elevation acute myocardial infarction: the VIRHISTAMI trial. EuroIntervention. 2013;8(10):1182-1189.

16.	Guo S, Wang R, Yang Z, Li K, Wang Q. Effects of atorvastatin on serum lipids, serum inflammation and plaque morphology in patients with stable atherosclerotic plaques. Exp Ther Med. 2012;4(6):1069-1074.

17.	Hong MK, Park DW, Lee CW, et al. Effects of statin treatments on coronary plaques assessed by volumetric virtual histology intravascular ultrasound analysis. JACC Cardiovasc Interv. 2009;2(7):679-688.

18.	Kawasaki M, Sano K, Okubo M, et al. Volumetric quantitative analysis of tissue characteristics of coronary plaques after statin therapy using three-dimensional integrated backscatter intravascular ultrasound. J Am Coll Cardiol. 2005;45(12):1946-1953.

19.	Kini AS, Baber U, Kovacic JC, et al. Changes in plaque lipid content after short-term intensive versus standard statin therapy: the YELLOW trial (reduction in yellow plaque by aggressive lipid-lowering therapy). J Am Coll Cardiol. 2013;62(1):21-29.

20.	Komukai K, Kubo T, Kitabata H, et al. Effect of atorvastatin therapy on fibrous cap thickness in coronary atherosclerotic plaque as assessed by optical coherence tomography: the EASY-FIT study. J Am Coll Cardiol. 2014;64(21):2207-2217.

21.	Park SJ, Kang SJ, Ahn JM, et al. Effect of Statin Treatment on Modifying Plaque Composition: A Double-Blind, Randomized Study. J Am Coll Cardiol. 2016;67(15):1772-1783.

22.	Schartl M, Bocksch W, Koschyk DH, et al. Use of intravascular ultrasound to compare effects of different strategies of lipid-lowering therapy on plaque volume and composition in patients with coronary artery disease. Circulation. 2001;104(4):387-392.

23.	Kadoglou NPE, Sailer N, Moumtzouoglou A, Kapelouzou A, Gerasimidis T, Liapis CD. Aggressive lipid-lowering is more effective than moderate lipid-lowering treatment in carotid plaque stabilization. J Vasc Surg. 2010;51(1):114-121.

24.	Takagi T, Yoshida K, Akasaka T, Hozumi T, Morioka S, Yoshikawa J. Intravascular ultrasound analysis of reduction in progression of coronary narrowing by treatment with pravastatin. Am J Cardiol. 1997;79(12):1673-1676.

25.	Underhill HR, Yuan C, Zhao XQ, et al. Effect of rosuvastatin therapy on carotid plaque morphology and composition in moderately hypercholesterolemic patients: a high-resolution magnetic resonance imaging trial. Am Heart J. 2008;155(3):584.e1-584.e8.

26.	Lo J, Lu MT, Ihenachor EJ, et al. Effects of statin therapy on coronary artery plaque volume and high-risk plaque morphology in HIV-infected patients with subclinical atherosclerosis: a randomised, double-blind, placebo-controlled trial. Lancet HIV. 2015;2(2):e52-e63.

27.	Cannon CP, Braunwald E, McCabe CH, et al. Intensive versus moderate lipid lowering with statins after acute coronary syndromes. N Engl J Med. 2004;350(15):1495-1504.

28.	Nakamura T, Obata JE, Kitta Y, et al. Rapid stabilization of vulnerable carotid plaque within 1 month of pitavastatin treatment in patients with acute coronary syndrome. J Cardiovasc Pharmacol. 2008;51(4):365-371.

29.	Lu MT, Ribaudo H, Foldyna B, et al. Effects of Pitavastatin on Coronary Artery Disease and Inflammatory Biomarkers in HIV: Mechanistic Substudy of the REPRIEVE Randomized Clinical Trial. JAMA Cardiol. 2024;9(4):323-334.

30.	Watanabe T, Kawasaki M, Tanaka R, et al. Anti-inflammatory and morphologic effects of pitavastatin on carotid arteries and thoracic aorta evaluated by integrated backscatter trans-esophageal ultrasound and PET/CT: a prospective randomized comparative study with pravastatin (EPICENTRE study). Cardiovasc Ultrasound. 2015;13:17.

31.	Watanabe K, Sugiyama S, Kugiyama K, et al. Stabilization of carotid atheroma assessed by quantitative ultrasound analysis in nonhypercholesterolemic patients with coronary artery disease. J Am Coll Cardiol. 2005;46(11):2022-2030.

32.	Nishiguchi T, Kubo T, Tanimoto T, et al. Effect of Early Pitavastatin Therapy on Coronary Fibrous-Cap Thickness Assessed by Optical Coherence Tomography in Patients With Acute Coronary Syndrome: The ESCORT Study. JACC Cardiovasc Imaging. 2018;11(6):829-838.

33.	Niki T, Wakatsuki T, Yamaguchi K, et al. Effects of the Addition of Eicosapentaenoic Acid to Strong Statin Therapy on Inflammatory Cytokines and Coronary Plaque Components Assessed by Integrated Backscatter Intravascular Ultrasound. Circ J. 2016;80(2):450-460.

34.	Hibi K, Sonoda S, Kawasaki M, et al. Effects of Ezetimibe-Statin Combination Therapy on Coronary Atherosclerosis in Acute Coronary Syndrome. Circ J. 2018;82(3):757-766.

35.	Pitt B, Mancini GB, Ellis SG, Rosman HS, Park JS, McGovern ME. Pravastatin limitation of atherosclerosis in the coronary arteries (PLAC I): reduction in atherosclerosis progression and clinical events. PLAC I investigation. J Am Coll Cardiol. 1995;26(5):1133-1139.

36.	Wang X, Zhao X, Li L, Yao H, Jiang Y, Zhang J. Effects of Combination of Ezetimibe and Rosuvastatin on Coronary Artery Plaque in Patients with Coronary Heart Disease. Heart Lung Circ. 2016;25(5):459-465.

37.	Oh PC, Jang AY, Ha K, et al. Effect of Atorvastatin (10 mg) and Ezetimibe (10 mg) Combination Compared to Atorvastatin (40 mg) Alone on Coronary Atherosclerosis. Am J Cardiol. 2021;154:22-28.

38.	Ueda Y, Hiro T, Hirayama A, et al. Effect of Ezetimibe on Stabilization and Regression of Intracoronary Plaque　- The ZIPANGU Study. Circ J. 2017;81(11):1611-1619.

39.	Kovarnik T, Mintz GS, Skalicka H, et al. Virtual histology evaluation of atherosclerosis regression during atorvastatin and ezetimibe administration: HEAVEN study. Circ J. 2012;76(1):176-183.

40.	Masuda J, Tanigawa T, Yamada T, et al. Effect of combination therapy of ezetimibe and rosuvastatin on regression of coronary atherosclerosis in patients with coronary artery disease. Int Heart J. 2015;56(3):278-285.

41.	Nakano Y, Yamamoto M, Matoba T, et al. Association between Serum Oxysterols and Coronary Plaque Regression during Lipid-Lowering Therapy with Statin and Ezetimibe: Insights from the CuVIC Trial. J Atheroscler Thromb. 2023;30(8):907-918.

42.	Tsujita K, Sugiyama S, Sumida H, et al. Impact of Dual Lipid-Lowering Strategy With Ezetimibe and Atorvastatin on Coronary Plaque Regression in Patients With Percutaneous Coronary Intervention: The Multicenter Randomized Controlled PRECISE-IVUS Trial. J Am Coll Cardiol. 2015;66(5):495-507.

43.	Hougaard M, Hansen HS, Thayssen P, et al. Influence of ezetimibe in addition to high-dose atorvastatin therapy on plaque composition in patients with ST-segment elevation myocardial infarction assessed by serial: Intravascular ultrasound with iMap: the OCTIVUS trial. Cardiovasc Revasc Med. 2017;18(2):110-117.

44.	Habara M, Nasu K, Terashima M, et al. Impact on optical coherence tomographic coronary findings of fluvastatin alone versus fluvastatin + ezetimibe. Am J Cardiol. 2014;113(4):580-587.

45.	Rossebø AB, Pedersen TR, Boman K, et al. Intensive lipid lowering with simvastatin and ezetimibe in aortic stenosis. N Engl J Med. 2008;359(13):1343-1356.

46.	Davidson MH, McGarry T, Bettis R, et al. Ezetimibe coadministered with simvastatin in patients with primary hypercholesterolemia. J Am Coll Cardiol. 2002;40(12):2125-2134.

47.	Baigent C, Landray MJ, Reith C, et al. The effects of lowering LDL cholesterol with simvastatin plus ezetimibe in patients with chronic kidney disease (Study of Heart and Renal Protection): a randomised placebo-controlled trial. Lancet. 2011;377(9784):2181-2192.

48.	Cannon CP, Blazing MA, Giugliano RP, et al. Ezetimibe added to statin therapy after acute coronary syndromes. N Engl J Med. 2015;372(25):2387-2397.

49.	Kim BK, Hong SJ, Lee YJ, et al. Long-term efficacy and safety of moderate-intensity statin with ezetimibe combination therapy versus high-intensity statin monotherapy in patients with atherosclerotic cardiovascular disease (RACING): a randomised, open-label, non-inferiority trial. Lancet. 2022;400(10349):380-390.

50.	Nicholls SJ, Puri R, Anderson T, et al. Effect of Evolocumab on Progression of Coronary Disease in Statin-Treated Patients: The GLAGOV Randomized Clinical Trial. JAMA. 2016;316(22):2373-2384.

51.	Nicholls SJ, Kataoka Y, Nissen SE, et al. Effect of Evolocumab on Coronary Plaque Phenotype and Burden in Statin-Treated Patients Following Myocardial Infarction. JACC Cardiovasc Imaging. 2022;15(7):1308-1321.

52.	Nicholls SJ, Puri R, Anderson T, et al. Effect of Evolocumab on Coronary Plaque Composition. J Am Coll Cardiol. 2018;72(17):2012-2021.

53.	Ako J, Hibi K, Tsujita K, et al. Effect of Alirocumab on Coronary Atheroma Volume in Japanese Patients With Acute Coronary Syndrome　- The ODYSSEY J-IVUS Trial. Circ J. 2019;83(10):2025-2033.

54.	Räber L, Ueki Y, Otsuka T, et al. Effect of Alirocumab Added to High-Intensity Statin Therapy on Coronary Atherosclerosis in Patients With Acute Myocardial Infarction: The PACMAN-AMI Randomized Clinical Trial. JAMA. 2022;327(18):1771-1781.

55.	Gao F, Wang ZJ, Ma XT, Shen H, Yang LX, Zhou YJ. Effect of alirocumab on coronary plaque in patients with coronary artery disease assessed by optical coherence tomography. Lipids Health Dis. 2021;20(1):106.

56.	Sugizaki Y, Otake H, Kawamori H, et al. Adding Alirocumab to Rosuvastatin Helps Reduce the Vulnerability of Thin-Cap Fibroatheroma: An ALTAIR Trial Report. JACC Cardiovasc Imaging. 2020;13(6):1452-1454.

57.	Yamamoto T, Sugizaki Y, Kawamori H, et al. Enhanced Plaque Stabilization Effects of Alirocumab　- Insights From Artificial Intelligence-Aided Optical Coherence Tomography Analysis of the Alirocumab for Thin-Cap Fibroatheroma in Patients With Coronary Artery Disease Estimated by Optical Coherence Tomography (ALTAIR) Study. Circ J. 2024;88(11):1809-1818.

58.	Tardif JC, Grégoire J, L’Allier PL, et al. Effects of the acyl coenzyme A:cholesterol acyltransferase inhibitor avasimibe on human atherosclerotic lesions. Circulation. 2004;110(21):3372-3377.

59.	Nicholls SJ, Puri R, Ballantyne CM, et al. Effect of Infusion of High-Density Lipoprotein Mimetic Containing Recombinant Apolipoprotein A-I Milano on Coronary Disease in Patients With an Acute Coronary Syndrome in the MILANO-PILOT Trial: A Randomized Clinical Trial. JAMA Cardiol. 2018;3(9):806-814.

60.	Marston NA, Bergmark BA, Prohaska TA, et al. Effect of APOC3 inhibition with olezarsen on coronary atherosclerosis: Essence-TIMI 73b imaging study. Circulation. 2026;153(20):1539-1548.

61.	Nissen SE, Lincoff AM, Brennan D, et al. Bempedoic acid and cardiovascular outcomes in statin-intolerant patients. N Engl J Med. 2023;388(15):1353-1364.

62.	O’Donoghue ML, Morrow DA, Vavere AL, et al. Antibody-mediated LOX-1 inhibition in patients with residual inflammation after myocardial infarction: a randomized phase 2 trial. Nat Med. 2025;31(10):3553-3559.

63.	UK Prospective Diabetes Study (UKPDS) Group. Effect of intensive blood-glucose control with metformin on complications in overweight patients with type 2 diabetes (UKPDS 34). Lancet. 1998;352(9131):854-865.

64.	Katakami N, Mita T, Maeda N, et al. Evaluation of the effect of tofogliflozin on the tissue characteristics of the carotid wall-a sub-analysis of the UTOPIA trial. Cardiovasc Diabetol. 2022;21(1):19.

65.	Kuramitsu S, Miyauchi K, Yokoi H, et al. Effect of sitagliptin on plaque changes in coronary artery following acute coronary syndrome in diabetic patients: The ESPECIAL-ACS study. J Cardiol. 2017;69(1):369-376.

66.	Li B, Luo YR, Tian F, et al. Sitagliptin attenuates the progression of coronary atherosclerosis in patients with coronary disease and type 2 diabetes. Atherosclerosis. 2020;300:10-18.

67.	Yamamoto H, Konishi A, Shinke T, et al. The impact of vildagliptin on the daily glucose profile and coronary plaque stability in impaired glucose tolerance patients with coronary artery disease: VOGUE-A multicenter randomized controlled trial. BMC Cardiovasc Disord. 2021;21(1):92.

68.	Nozue T, Fukui K, Koyama Y, et al. Effects of sitagliptin on coronary atherosclerosis in patients with type 2 diabetes-A serial integrated backscatter-intravascular ultrasound study. Am J Cardiovasc Dis. 2016;6(4):153-162.

69.	Nissen SE, Nicholls SJ, Wolski K, et al. Comparison of pioglitazone vs. glimepiride on progression of coronary atherosclerosis in patients with type 2 diabetes: the PERISCOPE randomized controlled trial. JAMA. 2008;299(13):1561-1573.

70.	Gerstein HC, Ratner RE, Cannon CP, et al. Effect of rosiglitazone on progression of coronary atherosclerosis in patients with type 2 diabetes mellitus and coronary artery disease: the assessment on the prevention of progression by rosiglitazone on atherosclerosis in diabetes patients with cardiovascular history trial. Circulation. 2010;121(10):1176-1187.

71.	Psaltis PJ, Nguyen MT, Singh K, et al. Optical coherence tomography assessment of the impact of colchicine on non-culprit coronary plaque composition after myocardial infarction. Cardiovasc Res. 2025;121(3):468-478.

72.	Ridker PM, Everett BM, Thuren T, et al. Antiinflammatory Therapy with Canakinumab for Atherosclerotic Disease. N Engl J Med. 2017;377(12):1119-1131.

73.	Yu M, Yang Y, Dong SL, et al. Effect of Colchicine on Coronary Plaque Stability in Acute Coronary Syndrome as Assessed by Optical Coherence Tomography: The COLOCT Randomized Clinical Trial. Circulation. 2024;150(13):981-993.

74.	Tardif JC, Kouz S, Waters DD, et al. Efficacy and safety of low-dose colchicine after myocardial infarction. N Engl J Med. 2019;381(26):2497-2505.

75.	Nidorf SM, Fiolet ATL, Mosterd A, et al. Colchicine in patients with chronic coronary disease. N Engl J Med. 2020;383(19):1838-1847.

76.	Jolly SS, d’Entremont MA, Lee SF, et al. Colchicine in acute myocardial infarction. N Engl J Med. 2025;392(7):633-642.

77.	Puri R, Nissen SE, Menon V, et al. Effects of aliskiren in diabetic and non-diabetic patients with coronary artery disease: Insights from AQUARIUS. Atherosclerosis. 2015;243(2):553-559.

78.	Nissen SE, Tuzcu EM, Libby P, et al. Effect of antihypertensive agents on cardiovascular events in patients with coronary disease and normal blood pressure: the CAMELOT study: a randomized controlled trial. JAMA. 2004;292(18):2217-2225.

79.	Hirohata A, Yamamoto K, Miyoshi T, et al. Impact of olmesartan on progression of coronary atherosclerosis a serial volumetric intravascular ultrasound analysis from the OLIVUS (impact of OLmesarten on progression of coronary atherosclerosis: evaluation by intravascular ultrasound) trial. J Am Coll Cardiol. 2010;55(10):976-982.

80.	Zanchetti A, Bond MG, Hennig M, et al. Calcium antagonist lacidipine slows down progression of asymptomatic carotid atherosclerosis: principal results of the European Lacidipine Study on Atherosclerosis (ELSA), a randomized, double-blind, long-term trial. Circulation. 2002;106(19):2422-2427.

81.	Luscher TF, Pieper M, Tendera M, et al. A randomized placebo-controlled study on the effect of nifedipine on coronary endothelial function and plaque formation in patients with coronary artery disease: the ENCORE II study. Eur Heart J. 2009;30(13):1590-1597.

82.	Lichtlen PR, Hugenholtz PG, Rafflenbeul W, Hecker H, Jost S, Deckers JW. Retardation of angiographic progression of coronary artery disease by nifedipine. Results of the International Nifedipine Trial on Antiatherosclerotic Therapy (INTACT). Lancet. 1990;335(8698):1109-1113.

83.	Yano H, Hibi K, Nozawa N, et al. Effects of valsartan, an angiotensin II receptor blocker, on coronary atherosclerosis in patients with acute myocardial infarction who receive an angiotensin-converting enzyme inhibitor. Circ J. 2012;76(6):1442-1451.

84.	Rodriguez-Granillo GA, de Winter S, Bruining N, et al. Effect of perindopril on coronary remodelling: insights from a multicentre, randomized study. Eur Heart J. 2007;28(19):2326-2331.

85.	MacMahon S, Sharpe N, Gamble G, et al. Randomized, placebo-controlled trial of the angiotensin-converting enzyme inhibitor, ramipril, in patients with coronary or other occlusive arterial disease. PART-2 Collaborative Research Group. Prevention of Atherosclerosis with Ramipril. J Am Coll Cardiol. 2000;36(2):438-443.

86.	Kojima T, Miyauchi K, Yokoyama T, et al. Azelnidipine and amlodipine anti-coronary atherosclerosis trial in hypertensive patients undergoing coronary intervention by serial volumetric intravascular ultrasound analysis in Juntendo University (ALPS-J). Circ J. 2011;75(5):1071-1079.

87.	Bhatt DL, Steg PG, Miller M, et al. Cardiovascular risk reduction with icosapent ethyl for hypertriglyceridemia. N Engl J Med. 2019;380(1):11-22.

88.	Alfaddagh A, Elajami TK, Ashfaque H, Saleh M, Bistrian BR, Welty FK. Effect of Eicosapentaenoic and Docosahexaenoic Acids Added to Statin Therapy on Coronary Artery Plaque in Patients With Coronary Artery Disease: A Randomized Clinical Trial. J Am Heart Assoc. 2017;6(12):e006981.

89.	Budoff MJ, Bhatt DL, Kinninger A, et al. Effect of icosapent ethyl on progression of coronary atherosclerosis in patients with elevated triglycerides on statin therapy: final results of the EVAPORATE trial. Eur Heart J. 2020;41(40):3925-3932.

90.	Nissen SE, Tardif JC, Nicholls SJ, et al. Effect of torcetrapib on the progression of coronary atherosclerosis. N Engl J Med. 2007;356(13):1304-1316.

91.	Fayad ZA, Mani V, Woodward M, et al. Safety and efficacy of dalcetrapib on atherosclerotic disease using novel non-invasive multimodality imaging (dal-PLAQUE): a randomised clinical trial. Lancet. 2011;378(9802):1547-1559.

92.	Nicholls SJ, Puri R, Wolski K, et al. Effect of the BET Protein Inhibitor, RVX-208, on Progression of Coronary Atherosclerosis: Results of the Phase 2b, Randomized, Double-Blind, Multicenter, ASSURE Trial. Am J Cardiovasc Drugs. 2016;16(1):55-65.

93.	Ahn J, Park SK, Park TS, et al. Effect of n-3 Polyunsaturated Fatty Acids on Regression of Coronary Atherosclerosis in Statin Treated Patients Undergoing Percutaneous Coronary Intervention. Korean Circ J. 2016;46(4):481-489.

94.	Xie X, Shi X, Zhang Y, et al. Angiopoietin-Like 3 Antibody Therapy in Patients With Suboptimally Controlled Hyperlipidemia: A Phase 2 Study. J Am Coll Cardiol. 2025;85(19):1821-1835.
